# The persistent multidomain health effects of the early-life exposome

**DOI:** 10.1126/sciadv.aec2089

**Published:** 2026-11-13

**Authors:** Augusto Anguita Ruiz, Ines Amine, Chongliang Luo, Alicia Guillien, María Arteaga, Adrià Setó-Llorens, Nikos Stratakis, Lea Maitre, Nuria Güil-Oumrait, Marina Ruiz-Rivera, Parisa Montazeri, Jose Urquiza, Mark Nieuwenhuijsen, Cathrine Thomsen, Line Småstuen Haug, Berit Granum, Sandra Andrusaityte, Regina Grazuleviciene, Audrius Dedele, Wenlun Yuan, Barbara Heude, Rosemary McEachan, Marina Vafeiadi, Leda Chatzi, John Wright, Tiffany C. Yang, Claire Philippat, Juan R. Gonzalez, Rémy Slama, Martine Vrijheid, Xavier Basagaña, Valérie Siroux

**Affiliations:** ^1^ Barcelona Institute for Global Health (ISGlobal), Barcelona, Spain.; ^2^ CIBER de Fisiopatología de la Obesidad y Nutrición (CIBEROBN), Instituto de Salud Carlos III, Madrid, Spain.; ^3^ Department of Biochemistry and Physiology, School of Pharmacy and Food Sciences, Universitat de Barcelona (UB), Barcelona, Spain.; ^4^ University Grenoble Alpes, Inserm U 1209, CNRS UMR 5309, Team of Environmental Epidemiology Applied to Development and Respiratory Health, Institute for Advanced Biosciences, Grenoble, France.; ^5^ Division of Public Health Sciences, Washington University School of Medicine in St. Louis, 600 S Taylor Ave., St. Louis, MO, USA.; ^6^ Department of Computer Science and Artificial Intelligence, University of Granada, Periodista Daniel Saucedo Aranda, Granada, Spain.; ^7^ Instituto de Investigación Biosanitaria ibs.GRANADA, Av. de Madrid, 15, Granada, Spain.; ^8^ Centro de Investigación Biomédica en Red de Epidemiología y Salud Pública (CIBERESP), Madrid, Spain.; ^9^ Pompeu Fabra University, Barcelona, Spain.; ^10^ Department of Food Safety, Norwegian Institute of Public Health (NIPH), Oslo, Norway.; ^11^ Department of Environmental Sciences, Vytautas Magnus University, Kaunas, Lithuania.; ^12^ Université Paris Cité and Université Sorbonne Paris Nord, Inserm, INRAE, Center for Research in Epidemiology and StatisticS (CRESS), Paris, France.; ^13^ Bradford Institute for Health Research, Bradford Teaching Hospitals NHS Foundation Trust, Bradford, UK.; ^14^ Department of Social Medicine, School of Medicine, University of Crete, Heraklion, Crete, Greece.; ^15^ Inserm, ENS-PSL, CNRS, IBENS (U1024), Paris, France.

## Abstract

The environments experienced early in life can shape health across multiple body systems, yet most research on exposome health effects has focused on isolated exposure windows and health outcomes, limiting understanding of their combined and enduring effects. Leveraging the Human Early Life Exposome (HELIX) European cohort—the largest exposome dataset from the general pediatric population—we studied 586 mother-child pairs with nearly 200 prenatal and childhood exposures, including air pollutants, chemicals, lifestyle (diet, physical activity, and toxic habits), and neighborhood outdoors factors. Associations between these exposures and 11 outcomes across mental health, cardiometabolic, respiratory, and allergic domains, assessed in both childhood (6 to 11 years) and adolescence (12 to 18 years) were sought using an outcome-wide exposure-wide analysis. We identified a set of “master regulator” exposures, such as maternal smoking, indoor air pollution, endocrine-disrupting chemicals, and dietary and urban factors, that influenced multiple health domains, with effects persisting into adolescence. By providing an atlas of exposome-health associations across developmental stages—available as an open-access interactive resource—this work pinpoints critical early-life risks and offers a platform to guide prevention and policy.

## INTRODUCTION

The theoretical framework of the developmental origins of health and disease (DOHaD) underscores the pivotal role of prenatal and early-life environmental exposures in shaping an individual’s long-term health (*1*). According to this theory, certain exposures have a more profound impact on health when they occur during specific vulnerability time windows. Life course epidemiology has been instrumental in investigating these associations and, more recently, embraced the exposome concept leading to a more comprehensive approach in the search for the root causes of diseases (*2*–*4*). The exposome concept acknowledges that individuals are constantly exposed to numerous environmental hazards, which cumulatively impact their health (*3*–*6*). This holistic framework integrates the external exposome—comprising chemical and physical stressors such as chemical pollutants, built environments, and climate and biological components such as microbial patterns—and the personal exposome, which includes lifestyle factors such as diet, sleep, physical activity, or toxic habits (*7*).

Some prenatal and early-life environmental exposures, such as tobacco, have been recognized to exert detrimental effects not on a single but on multiple health outcomes simultaneously (*8*–*10*). However, most exposome research conducted to date has traditionally focused on the study of different health outcomes separately, under what is known as the single-outcome approach (*11*, *12*). Although single-outcome analysis is a valid approach for assessing exposome-health associations, it may not provide a comprehensive enough picture of environmental health effects, especially for exposures that are presumed to have multiple health impacts. An outcome-wide analysis, in contrast, simultaneously investigates the effect of an exposure on multiple health outcomes (*13*–*16*). Recently, advanced machine learning methods have been proposed to extend the outcome-wide analysis to the exposome concept, and guidelines for these analyses have been provided (*17*, *18*). Outcome-wide exposome analysis offers methodological advantages over traditional single-outcome exposome approaches by avoiding multiple testing, the selective reporting of findings and by taking advantage of all the data and correlations between variables to make better inferences. Outcome-wide exposome approaches target exposures that simultaneously affect multiple health domains and may thus be of higher priority to public health authorities and environmental regulators.

European exposome research initiatives, such as the population-based Human Early Life Exposome (HELIX) project (*19*), which generated one of the largest exposome cohort datasets of the general pediatric population, have produced extensive evidence on the impact of prenatal and early life exposures on various health domains, including neurodevelopment and cognition, respiratory health, obesity, blood pressure, allergy, and mental health disorders, using a single-outcome approach (*10*, *20*–*26*). Although valuable in understanding environmental influences on health risk, these studies typically measured outcomes at only one point in childhood, making it difficult to determine whether effects persist into adolescence and thereby limiting causal interpretation.

Here, we conducted a prospective outcome-wide exposome study including a broad spectrum of early life environmental exposures and multiple health outcomes, spanning various health domains, assessed at multiple stages of development (i.e., childhood and adolescence) (*27*). Our study design therefore establishes a clear temporal sequence between measured exposures and health outcomes (pregnancy exposure–childhood outcomes and childhood exposure–adolescent outcomes). Furthermore, repeated measurements of outcomes across the first two decades of early life help assess the persistence of the detrimental health effects caused by early-life environmental exposures.

## RESULTS

### Study population and assessed health domains

An overview of the methodological workflow adopted in this study is illustrated in Fig. 1. We used data from the HELIX project (*19*, *27*)_,_ which gathers six established and ongoing longitudinal population-based birth cohorts: Born in Bradford (BiB, UK), Étude des Déterminants pré et postnatals du développement et de la santé de l’Enfant (EDEN, France), Infancia y Medio Ambiente (INMA, Spain), Kaunas Cohort (KANC, Lithuania), The Norwegian Mother, Father and Child Cohort Study (MoBa, Norway) (*28*), and Mother-Child Cohort (RHEA, Greece). Our study included 586 mother-child pairs with available data on environmental exposures assessed at pregnancy and childhood (6 to 11 years) and on clinical and preclinical health outcomes at childhood (6 to 11 years) and adolescence (12 to 18 years) (Table 1). Mean (SD) maternal age and prepregnancy body mass index (BMI) were 30.8 (4.8) years and 25 (4.9) kg/m^2^. Half of the mothers were highly educated (53.3%) and 46.1% were nulliparous. Mean (SD) age of the children, among which 46.8% were girls, was 7.8 (1.6) years at the childhood stage and 15 (1.5) years at the adolescent follow-up. We investigated 11 preclinical and clinical health outcomes belonging to four health outcome areas that are known to be linked to noncommunicable disease risk in later life and that represent prevalent health endpoints in European children: (i) mental health, (ii) the cardiometabolic system, (iii) the respiratory system, and (iv) the immune system (table S11). As shown in Table 2, most of the health domains showed better health parameters in adolescence compared to childhood follow-up (Table 2). For instance, attention deficit hyperactivity disorder (ADHD), externalizing problems, elevated systolic blood pressure, asthma medication, wheezing, and dermatitis had lower prevalence in adolescence as compared to childhood. Descriptive statistics by-cohort of recruitment can be found in table S15. In fig. S3, we show the correlation matrix for health outcomes at different time points. As expected, health outcomes correlated across time points (ρ ∼ 0.5) and correlations were stronger (although still moderate) within health domains (ρ ∼ 0.6).

**Fig. 1.**
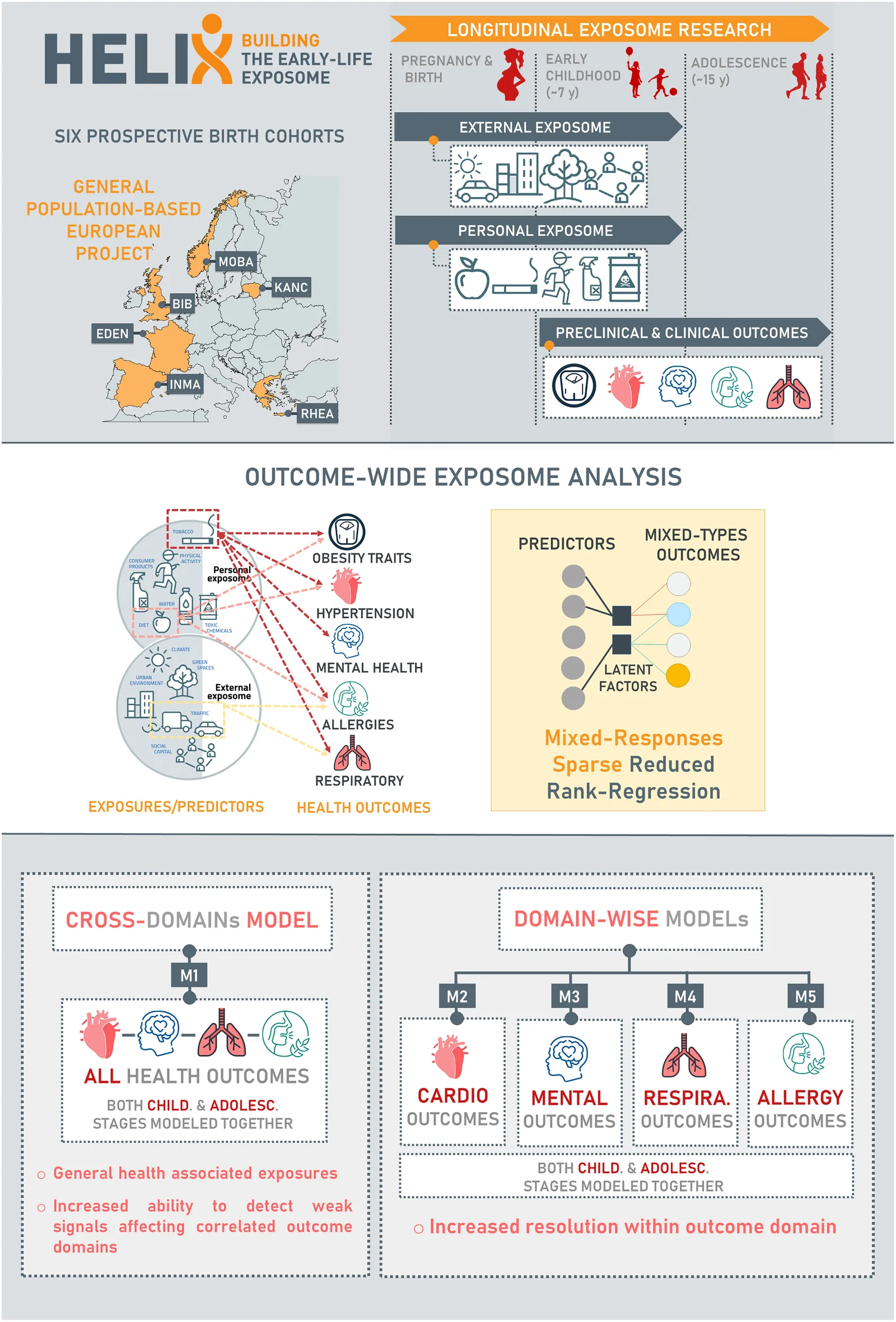
Analytic workflow of the study. Mixed-responses sparse reduced-rank generalized linear regression (msRRR) was applied separately to pregnancy and childhood exposures. Outcomes from childhood and adolescence stages were always modeled together. The msRRR was applied following two distinct approaches. In the cross-domain approach, all outcomes were modeled simultaneously (regardless of the health domain they belong to). In the domain-wise approaches, a different msRRR model was applied to each subgroup of outcomes (e.g., cardiometabolic, mental health, respiratory, and allergy). Although the cross-domain model allows the identification of environmental exposures affecting different health domains simultaneously, domain-wise models permit deepening into associations within each health family, highlighting exposures affecting multiple traits within the same domain. Models were run separately for pregnancy and childhood exposures. In total, 10 separate msRRR models were constructed. y, years.

**Table 1.** General descriptive of the HELIX study population. Abbreviations: BMI, body mass index; BiB, Born in Bradford; EDEN, Étude des Déterminants pré et postnatals du développement et de la santé de l’Enfant; INMA, Infancia y Medio Ambiente; IOTF, International Obesity Task Force; KANC, Kaunas Cohort; MoBa, The Norwegian Mother, Father and Child Cohort Study; RHEA, Mother-Child Cohort in Crete.

	Mean (SD) or *N* (%)
	***N* = 586**
Child’s age (years)	
Childhood	7.85 (1.6)
Adolescence	15.0 (1.5)
Child’s height (m)	
Childhood	1.28 (0.1)
Adolescence	1.66 (0.09)
Child’s sex	
Male	312 (53.2%)
Female	274 (46.8%)
Cohort	
RHEA	77 (13.1%)
BIB	116 (19.8%)
EDEN	76 (13.0%)
INMA	156 (26.6%)
KANC	111 (18.9%)
MOBA	50 (8.5%)
Is the family native from the country of recruitment?	
Any	68 (11.6%)
One parent	41 (7.0%)
Both parents	477 (81.4%)
Maternal education	
Low (primary school)	81 (13.8%)
Middle (secondary school)	193 (32.9%)
High (university degree or higher)	312 (53.3%)
Maternal prepregnancy BMI (kg/m^2^)	25.1 (4.9)
Maternal age at pregnancy (years)	30.8 (4.8)
Parity	
Nulliparous	270 (46.1%)
Primiparous	207 (35.3%)
Multiparous	109 (18.6%)
Breastfeeding duration (months)	4.97 (1.14)
Season of conception	
First trimester of the year	173 (29.5%)
Second trimester of the year	99 (16.9%)
Third trimester of the year	136 (23.2%)
Forth trimester of the year	178 (30.4%)
Paternal education	
Low (primary school)	117 (20.0%)
Middle (secondary school)	229 (39.1%)
High (university degree or higher)	240 (41.0%)
Child ethnicity	
Asian	66 (11.3%)
Caucasian	507 (86.5%)
Others	13 (2.2%)
Nursery attendance < 2 years old (yes)	356 (60.8%)
Birth weight, in grams	3344 (469)

**Table 2.** Descriptive of the multiple health domains in the study population. *P* values were obtained from a paired *t* test analysis for continuous outcomes and from McNemar’s test for binary outcomes. Abbreviations: ADHD, attention deficit hyperactivity disorders; BMI, body mass index; CBCL, Child Behavior Checklist; DSM-IV, Diagnostic and Statistical Manual of Mental Disorders; DBP, diastolic blood pressure; SBP, systolic blood pressure.

	Childhood	Adolescence	*P* value
	***N* = 586**	***N* = 586**	
Cardiometabolic health outcomes	Mean (SD)	Mean (SD)	
DBP z-score	0.03 (0.86)	0.16 (0.85)	0.009
“Missing”	0 (0.00%)	28 (4.7%)	
SBP z-score	0.23 (1.03)	0.00 (1.01)	<0.001
“Missing”	0 (0.00%)	28 (4.7%)	
BMI z-score	0.36 (1.17)	0.42 (1.23)	0.443
“Missing”	0 (0.00%)	2 (0.3%)	
Mental health outcomes	Mean (SD)	Mean (SD)	
Externalizing problems – Scale (CBCL)	6.52 (6.4)	5.76 (6.4)	0.044
“Missing”	1 (0.2%)	7 (1.2%)	
Internalizing problems – Scale (CBCL)	6.65 (5.8)	7.69 (7.08)	0.006
“Missing”	1 (0.2%)	7 (1.2%)	
ADHD index (DSM-IV)	3.05 (2.9)	2.46 (2.7)	<0.001
“Missing”	1 (0.2%)	7 (1.2%)	
Respiratory health outcomes	*N* (%)	*N* (%)	
Asthma (ever):			0.001
No	530 (90.4%)	500 (85.3%)	
Yes	56 (9.6%)	75 (12.8%)	
“Missing”	0 (0.0%)	11 (1.9%)	
Asthma medication in the last 12 months:			<0.001
No	488 (83.3%)	521 (88.9%)	
Yes	98 (16.7%)	51 (8.7%)	
“Missing”	0 (0.0%)	14 (2.4%)	
Wheezing in the last 12 months:			<0.001
No	505 (86.2%)	535 (91.3%)	
Yes	81 (13.8%)	44 (7.5%)	
“Missing”	0 (0.00%)	7 (1.2%)	
Allergy traits	*N* (%)	*N* (%)	
Dermatitis in the last 12 months:			<0.001
No	444 (75.8%)	463 (79.0%)	
Yes	142 (24.2%)	111 (18.9%)	
“Missing”	0 (0.0%)	12 (2.05%)	
Rhinitis in the last 12 months:			0.012
No	444 (75.8%)	423 (72.2%)	
Yes	142 (24.2%)	156 (26.6%)	
“Missing”	0 (0.0%)	7 (1.2%)	

### Outcome-wide analysis of pregnancy exposures

To investigate how prenatal environments shape child and adolescent health, we applied an outcome-wide exposome analysis. Unlike standard exposome-wide approaches that test each exposure against one outcome at a time, the outcome-wide framework simultaneously evaluates the impact of multiple exposures across multiple health domains under the same model, helping to identify “master regulators” with broad effects. Using data from 69 prenatal exposures mapping 16 families, we modeled their associations with 11 outcomes spanning cardiometabolic, mental, respiratory, and allergic health in childhood and adolescence (Fig. 1 and table S1). Two complementary strategies were applied: a cross-domain model including all outcomes together and domain-specific models focusing on each health system separately. Although the cross-domain model allows the identification of environmental exposures affecting different health domains simultaneously, domain-wise models permit deepening into associations within each health family, highlighting exposures affecting multiple traits within the same domain (Fig. 1).

Pregnancy exposures explained a modest but consistent fraction of variance in outcomes, with respiratory and mental health outcomes being the best represented by pregnancy exposures (*R*^2^ up to 5.2 and 6.6% in domain-wise models, respectively, reaching percentages of >10% for specific outcomes), followed by cardiometabolic outcomes (*R*^2^ ≈ 1.5%), whereas allergy outcomes showed no detectable associations (fig. S13 and table S16). All models performed consistently in training and test sets, showing no evidence of overfitting (table S17). Figure 2 presents the individual regression coefficients for each outcome–exposure pair, providing a detailed map of associations. Many associations identified at childhood (6 to 11 years) persisted into adolescence (12 to 18 years), with effects in the same direction. For example, prenatal exposure to maternal smoking was consistently associated with higher offspring BMI *z*-scores and externalizing behaviors in both follow-ups, as well as increased ADHD symptoms in adolescence (Fig. 2B). Likewise, high maternal meat intake was linked to all adverse mental health outcomes and to asthma in children across both periods, as well as to higher prevalence of wheezing and dermatitis in childhood.

**Fig. 2.**
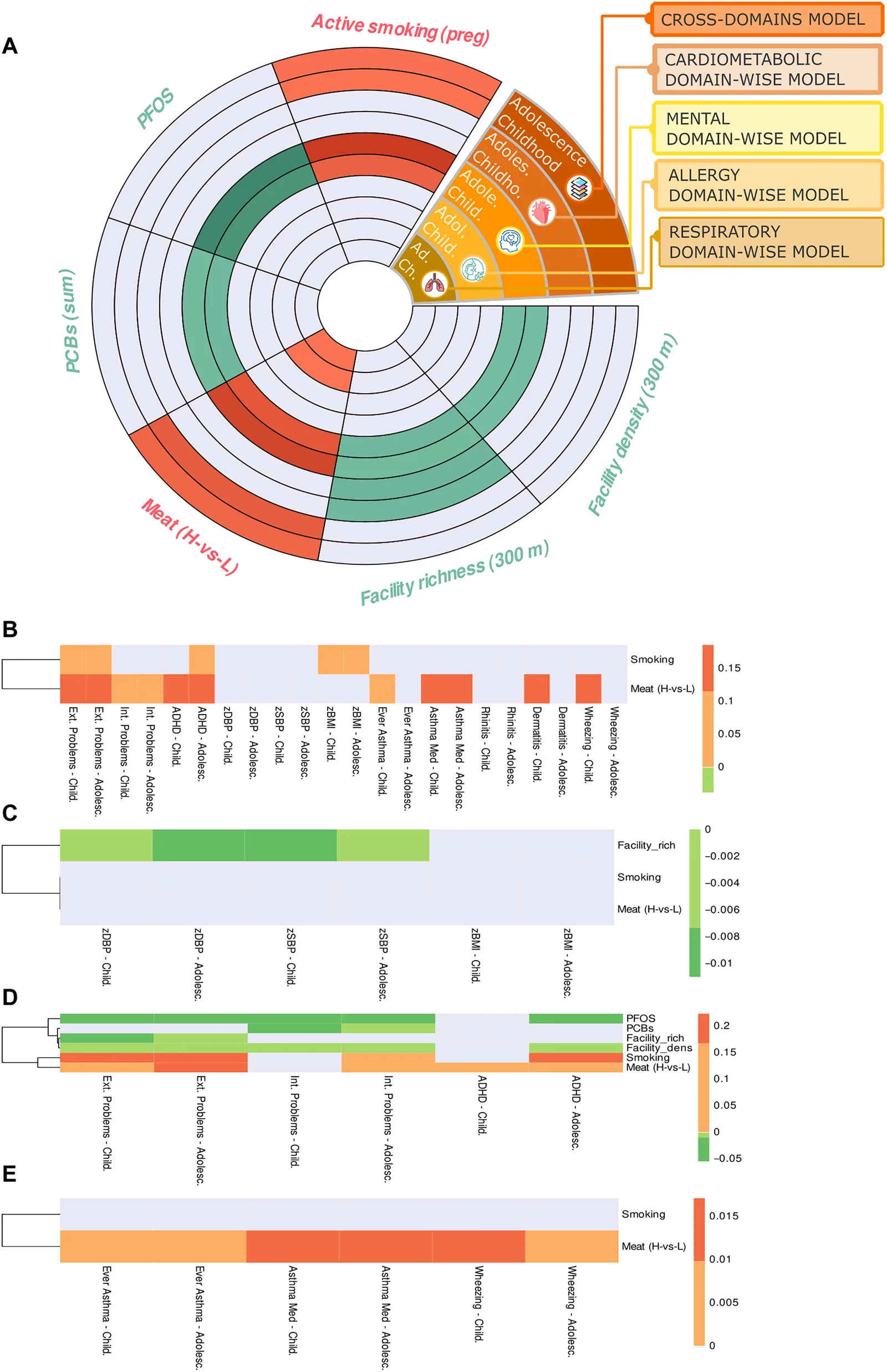
Outcome-wide associations for pregnancy exposures. To allow the comparison between exposures and outcomes, standardized beta coefficients are presented. We calculated the inverse of health outcomes in which higher values refer to a healthier status, so all beta coefficients can be interpreted in the same way (red: increased risk; green: decreased risk; gray: nonsignificant after bootstrapping). (**A**) Summary of all associations encountered from all outcome-wide approaches for pregnancy exposures (see Materials and Methods for details on approaches). In this circle heatmap, red or green cell colors indicate that a certain exposure presented at least one outcome association within the approach. Individual exposure-outcome associations can be inspected in detail in (B) to (E) for each msRRR approach. (**B**) msRRR cross-domain approach (overall health). (**C**) msRRR domain-wise cardiometabolic. (**D**) msRRR domain-wise mental health. (**E**) msRRR domain-wise respiratory health. No associations were identified for domain-wise allergy health.

Domain-specific models yielded complementary insights. In total, one, six, and one exposures were associated with cardiometabolic, mental, and respiratory domains, respectively. These models revealed additional associations not captured in the cross-domain approach (Fig. 2, C to E). Notably, two urban environmental factors (facility density and richness in residential area during pregnancy) and two persistent chemicals (levels of PCBs and PFOS in pregnant mothers’ blood) were simultaneously associated with improved offspring outcomes within cardiometabolic and mental domains, with effects observed consistently in both childhood and adolescence stages.

To assess the robustness of these associations to cohort composition, we conducted a leave-one-cohort-out (LOCO) sensitivity analysis, systematically refitting all outcome-wide models across six iterations, each excluding one of the six HELIX cohorts, and applying an additional bootstrap resampling step within each iteration to derive sign-stability thresholds. We considered an exposure-outcome association robust when it was selected in most sensitivity analysis iterations (>4- of 6-fold) with a consistent effect direction, and when it demonstrated cross-regional generalizability, that is, when it survived the sequential exclusion of cohorts from both the South-Mediterranean (INMA and RHEA) and North-Western European (MoBa, KANC, BiB, and EDEN) areas. This second criterion is motivated by the fact that these two macroregions represent distinct socioeconomic structures, dietary styles, and urban configurations in Europe that collectively might drive the distribution of most early-life exposures, such that associations surviving only within one regional block may reflect specific cultural or regional phenomena rather than general effects (fig. S16). Under this approach, the large majority of exposures identified as “master regulators” in the main outcome-wide analysis were replicated across all six iterations and across both European macroregions, supporting their generalizability beyond any single countries and geographic context. Notably, some exceptions were identified for domain-wise models; residential facility density and richness during pregnancy with cardiometabolic and mental health outcomes and meet consumption during pregnancy and respiratory health, which resulted to be sensible to the removal of specific cohorts and regions. Therefore, these findings may reflect cohort- or region-specific phenomena.

### Outcome-wide analysis of childhood exposures

We next applied the outcome-wide framework to the 129 exposures measured in childhood (ages 6 to 11) and spanning 19 exposure domains, prospectively linking them to the 11 health outcomes assessed both at the same age and later in adolescence. Table S17 summarizes the performance of the childhood models, whereas explained variances for each outcome are shown in fig. S14 and table S18. Childhood exposures explained a larger fraction of outcome variance than prenatal exposures, particularly for mental and cardiometabolic domains (*R*^2^ up to 14.8 and 15.5% in domain-wise models, respectively, reaching for some specific outcomes >20%), whereas respiratory and allergy outcomes were more weakly explained (*R*^2^ ≈ 1 to 4%).

The cross-domain analysis identified 16 exposures associated with multiple health domains (Fig. 3, A and B). Most of these were related to dietary habits (12 of 16, 75%), although indoor air pollutants and heavy metals also emerged. For instance, higher indoor PM_2.5_ and elevated blood copper levels were associated with adverse cardiometabolic and mental health profiles. Similarly, maternal perception of child stress was linked to worse mental health outcomes in both childhood and adolescence and to higher BMI and diastolic blood pressure at childhood. In contrast, moderate consumption of diverse food groups (fish, caffeinated drinks, fast food, sweets, among others) and high intake of cereals and yogurt were associated with healthier respiratory and allergy outcomes.

**Fig. 3.**
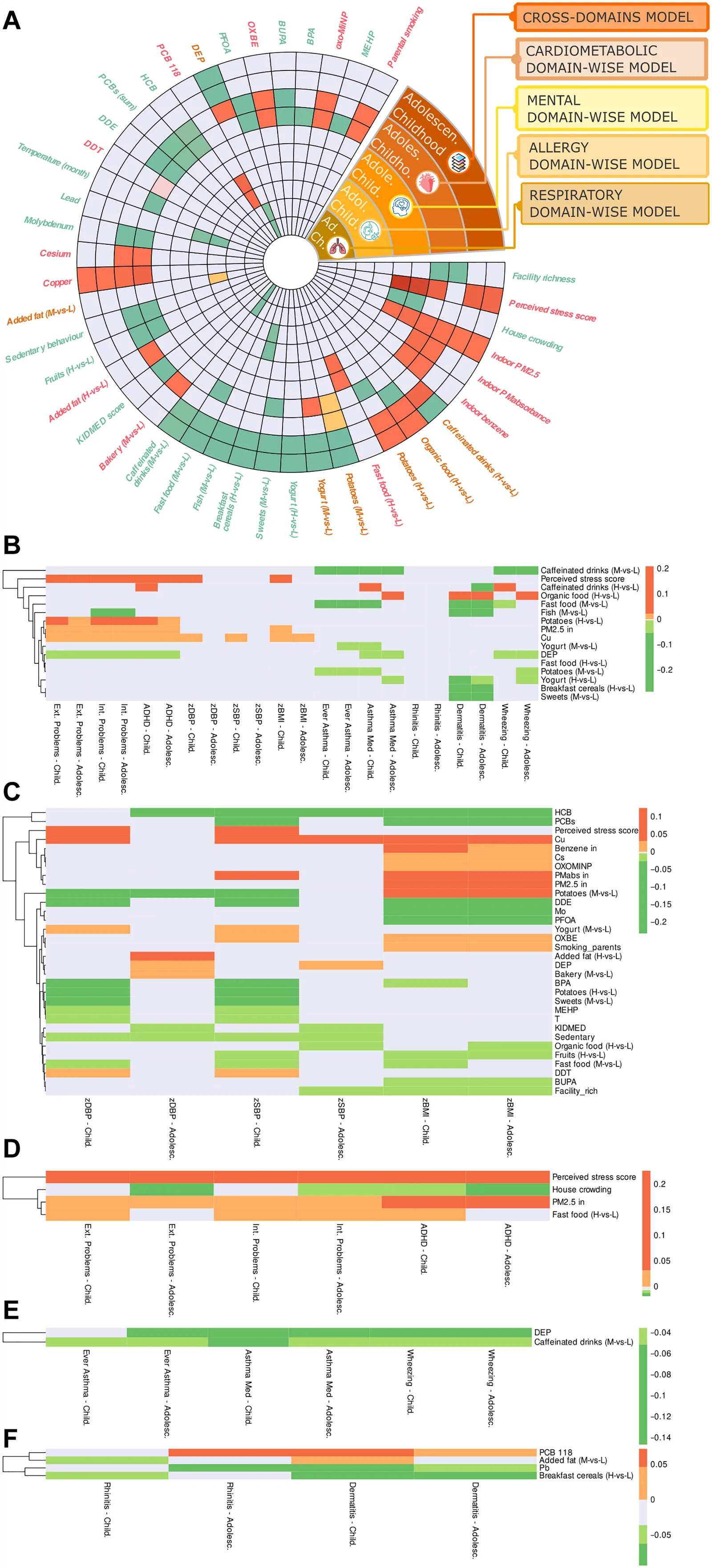
Outcome-wide associations for childhood exposures. To allow the comparison between exposures and outcomes, standardized beta coefficients are presented. We calculated the inverse of health outcomes in which higher values refer to a healthier status, so all beta coefficients can be interpreted in the same way (red: increased risk; green: decreased risk; gray: nonsignificant after bootstrapping). (**A**) Summary of all associations encountered from all outcome-wide approaches for childhood exposures (please see Materials and Methods for more details on approaches). In this circle heatmap, red or green colors for cells indicate that a certain exposure presented at least one outcome association within the approach. Individual exposure-outcome associations can be inspected in detail in (B) to (F) for each msRRR approach. (**B**) msRRR cross-domain approach (overall health). (**C**) msRRR domain-wise cardiometabolic. (**D**) msRRR domain-wise mental health. (**E**) msRRR domain-wise respiratory health. (**F**) msRRR domain-wise allergy.

Domain-specific models provided greater resolution, selecting 32, 4, 2, and 4 exposures for cardiometabolic, mental, respiratory, and allergy outcomes, respectively (Fig. 3, C to F). Within the cardiometabolic domain, passive smoking, several phthalates and phenols (e.g., oxo-MiNP and OXBE), and indoor pollutants such as benzene and particulate matter were consistently associated with higher BMI or blood pressure across both life stages. Pesticides and pesticide-derived metabolites (DDT and DEP) also predicted higher blood pressure in childhood and adolescence. By contrast, adherence to a Mediterranean diet (higher KIDMED scores and greater fruit intake) and living in areas with more facilities were protective, being associated with lower BMI and blood pressure. As in pregnancy, some persistent chemicals (HCB, PCBs, PFOA, and DDE) showed paradoxical associations with lower BMI and blood pressure. The other domain-wise models were consisted with findings from the cross-domain approach while adding specific signals. For instance, high fast-food intake was associated with worse mental health, and PCB-118 exposure was associated with poorer allergy outcomes (Fig. 3, D to F). Higher household crowding—a socioeconomic marker—was associated with better mental health outcomes.

LOCO sensitivity analysis for childhood exposure models broadly confirmed the robustness of most of the associations described above. Across both the cross-domain and domain-wise models, most reported exposure-outcome pairs were selected in more than four of the six cohort iterations with a consistent effect direction and cross-regional generalizability (South-Mediterranean and North-Western Europe), supporting their status as European-wide signals rather than cohort-driven artifacts (fig. S18). This included the multidomain associations of perceived stress, indoor PM_2.5_, blood copper, and most dietary exposures identified in the cross-domain model, as well as the protective cardiometabolic signatures of Mediterranean diet adherence, fruit intake, and residential facility access. Similarly, the paradoxical inverse associations of persistent organic pollutants (POPs; HCB, PCBs, PFOA, and DDE) with BMI and blood pressure remained consistently selected. Notable exceptions were the associations of phthalates and phenols, specifically oxo-MiNP and OXBE, with cardiometabolic outcomes, and those of high fast-food intake with mental health, neither of which met the prespecified sensitivity criteria. These associations may reflect cohort- or region-specific exposure associations rather than general effects and should therefore be interpreted with caution.

## DISCUSSION

The exposome concept is increasingly recognized for its vast potential in studies involving multiple health outcomes with complex and unknown causes. It offers a valuable framework for identifying critical windows of exposure and environmental risk factors, which can inform policy interventions. Here, using data from the HELIX population, we conducted a prospective outcome-wide exposome study spanning pregnancy through adolescence and across multiple health domains. By creating an atlas of exposome-health associations in children and adolescents (https://outcomewide.isglobal.org/), we provide an open resource to guide prevention, policy, and future research. Six prenatal and 42 childhood exposures showed evidence of multioutcome influence, with effects persisting from childhood into adolescence. LOCO sensitivity analysis, coupled with a cross-regional robustness filter distinguishing South-Mediterranean from North-Western European cohorts, demonstrated that the large majority of reported associations are stable to cohort composition and generalizable across distinct European socioenvironmental contexts. These findings highlight the potential of early-life exposures to shape enduring health trajectories.

The four health domains covered in our study share important common biological pathways, most notably systemic inflammation. This shared biological underpinning likely explains why these conditions often co-occur in patterns of comorbidity; for instance, children with obesity are not only more prone to asthma but also face a higher risk of mental health issues due to both psychosocial stigma and underlying inflammatory cross-talk (*29*). At the domain level, mental and cardiometabolic outcomes were most strongly explained by early-life exposures (with several individual traits exceeding an *R*^2^ of 25%), whereas respiratory outcomes showed weaker associations and allergy outcomes were scarcely related to the exposome. These differences are consistent with prior single-outcome studies in HELIX and other European cohorts, which have repeatedly shown robust environmental contributions to obesity and behavioral traits, but more heterogeneous findings for allergic endpoints (*10*, *20*–*26*). Most associations in our study originated from childhood exposures, although respiratory outcomes were more strongly explained by the prenatal exposome, suggesting that critical exposure windows might differ by health domain (*2*). From a mechanistic perspective, this disparity is revealing. The high variance explained in the cardiometabolic and mental domains suggests that the chemical and lifestyle factors we measured, such as air pollution, chemical, and diet, are potent triggers of what has been termed “immune-health convergence” (*7*). These exposures act as chronic stimuli that prime the systemic inflammation that drives metabolic dysfunction and neurobehavioral traits. Conversely, our more heterogeneous findings for respiratory and allergic endpoints may indicate that these specific domains are more sensitive to the ‘biological’ dimension of the exposome (e.g., microbial diversity and pathogen exposure), which was not captured in our study.

Among prenatal exposures, maternal smoking was associated with higher BMI and externalizing behaviors at childhood and adolescence, extending into ADHD symptoms at adolescence. These associations reinforce existing epidemiological and experimental evidence linking tobacco exposure with both metabolic and neurobehavioral risks (*20*, *24*, *30*–*32*). Maternal meat intake during pregnancy was associated with adverse respiratory and mental health outcomes, in line with some (*33*, *34*) but not all previous studies (*35*–*39*), and may reflect immunological programming pathways (*40*, *41*). Urban features, particularly residential facility density and richness during pregnancy, were related to lower blood pressure and better mental health, consistent with research highlighting built environments as determinants of physical activity and social interactions (*20*, *22*, *24*). However, these associations showed limited cross-regional robustness in sensitivity analyses, suggesting they may partly reflect geographically specific built environment contrasts rather than universal causal effects. Regarding our findings for prenatal PCBs exposure, the current state of evidence remains insufficient to draw conclusions about obesity outcomes (*20*, *42*, *43*). A recent study has shown inconsistent, or null results, with some evidence for sex-specific effects that we may have overlooked due to the limited statistical power in our study (*42*). Furthermore, these findings may suggest a mode of action that operates at lower doses, in line with the nonmonotonic relationships observed between endocrine-disrupting chemicals (EDCs) and hormonal activity.

During childhood, exposures showed broader impacts. Indoor PM_2.5_ and blood copper were associated with adverse cardiometabolic and mental outcomes at childhood and adolescence, echoing studies linking pollution and metals with systemic inflammation and organ dysfunction (*44*–*49*). We observed that passive smoking exposure, exposure to phthalates and phenols (such as oxo-MiNP and OXBE), and some indoor pollutants (such as benzene or particulate matter) were associated with higher BMI and blood pressure *z*-scores in both childhood and adolescence. In contrast, higher adherence to a Mediterranean diet and greater fruit intake were linked to healthier cardiometabolic profiles, reinforcing dietary quality as a modifiable protective factor (*22*, *42*, *50*). As previously discussed, diet, especially during early life, may influence health traits through its effects on lipid metabolism and immune axis maturation. This process aligns with the concept of “immune-health convergence,” where early-life nutritional quality serves as a fundamental primer for the developing immune system. Furthermore, there is evidence on how children’s dietary habits indirectly reflect maternal dietary patterns during pregnancy (*51*), so it might be possible that our reported association for child’s dietary habits actually partly reflects previous dietary exposures from the immunologically relevant period of pregnancy, in which diet was not fully characterized in our study. During these critical windows, dietary constituents likely operate in synergy with other chemical stressors and the assembly of the early-life microbiome to modulate the inflammatory tone (*7*), potentially explaining the persistent multidomain effects observed as children transition into adolescence. Associations with phthalates and phenols, as well as with fast-food intake in the mental health domain, showed lower replication across cohort in sensitivity analyses and may reflect exposure contrasts that are specific to particular study populations. As in pregnancy, we also found inverse unexpected associations for some persistent chemical compounds in children’s samples (mainly DDE and PCBs). These chemicals, known as POPs, are highly lipophilic (stored in fat tissue), and the amount of body fat and growth trajectories of children are expected to affect their toxicokinetics in blood. This suggests that serum or plasma may not be the most appropriate biological matrix for measuring these compounds and that fat tissue samples would provide more accurate assessments (*52*). Because these chemical were measured in children’s blood in our study, residual confounding or reverse causation in the POPs-health associations may be present, and these results should be interpreted with caution (*52*).

This study has several strengths. Our outcome-wide framework goes beyond running multiple single-outcome exposome-wide association study (ExWAS) in parallel. By applying a mixed-responses sparse reduced-rank regression (msRRR) (*17*, *18*), we jointly modeled correlated outcomes, reduced dimensionality through latent factors, and mitigated risks of multicollinearity and multiple testing. This approach enabled identification of exposures with cross-domain influence that might be overlooked in univariate approaches. Moreover, outcome-wide approaches such as ours hold substantial public health value as exposures that impact multiple health domains are important contributors to the overall burden of disease. Our study includes a comprehensive assessment of a large range of environmental exposures in two critical developmental periods (pregnancy and childhood) under an exposome vision that comprises many different domains. It involves a prospective follow-up design with health outcomes assessed at two different periods (childhood and adolescence), which leaves a clear temporal sequence between exposure and health outcomes (pregnancy exposures versus childhood outcomes and childhood exposures versus adolescent outcomes).

Our study also has some limitations. First, although the HELIX cohort is one of the most comprehensive early life exposome cohorts and includes populations in six countries, our study had a moderate sample size and highly selective population, which may limit the generalizability of our findings. The moderate sample size also prevented us from performing stratification analyses, for example, to explore possible sex-specific effects. Second, the fact that nonlinear effects or interactions were not considered in this study (also due to lack of statistical power and the number of evaluated exposures) hinders the possibility of detecting potential U-shaped relationships (typically observed for chemicals). Furthermore, although the msRRR framework allows for the identification of latent components that account for the aggregated effect of multiple coexposures, we acknowledge the absence of formal exposome mixture modeling. Such models could further characterize the simultaneous and potentially synergistic effects of multiple exposures. However, the application of traditional mixture methods to high-dimensional, outcome-wide longitudinal data remains limited, and future studies should aim to integrate these approaches as the methodology matures. Third, as evidenced by the unexpected direction of some of our associations, it is important to consider that our study may have been affected by residual confounding (*53*). Specifically, the lack of explicit mediation, moderation, or modification effects in our analyses may partly explain some associations resulting in nonhypothesized directions, such as the inverse relationships observed for certain POPs. As recently demonstrated in massive exposomic scans (*54*), inferred environmental relationships are highly sensitive to unmodeled interactions and confounding control, frequently leading to reversed associations, which highlights the need for future research that explicitly explore complex interactive and mediation pathways to fully disentangle true causal effects from residual confounding, distinct toxicokinetics, or reverse causality. Fourth, some of our analyses were exposed to potential measurement errors, particularly those related to highly variable nonpersistent chemicals measured in one or a few urine samples, that may have led to over- and underestimated health effect associations (*55*, *56*). Still, all chemical analyses were performed at the same laboratory at both follow-ups reducing external sources of batch effects and variability. Also, our characterization of the exposome focused primarily on chemical, physical, and lifestyle factors, leaving out the biological dimension (e.g., bacteria, viruses, and fungi), which may limit our understanding of the full interplay between the environment and health trajectories. Last, regarding health outcomes, although we covered a comprehensive range of health domains, there were some clinical outcomes within some of the domains without available information in our population (mainly cognitive function and metabolic health scores).

Despite these limitations, our observations complement existing global efforts to document exposure-phenotype relationships. For example, they resonate with a recently published landmark atlas of exposome-phenome associations from Patel *et al.* (*54*), which systematically mapped 619 exposure indicators against 305 quantitative phenotypes across ten independent waves of the US NHANES. Several key findings from this work converge with our results; notably, replicable signals were most concentrated in cardiometabolic and anthropometric phenotypes, whereas smoking and dietary biomarkers emerged as the exposure categories most broadly implicated across the phenome. Patel *et al.* (*54*) also documented a dense correlational architecture among exposures, a complexity that complicates causal attribution and identification of outcome-wide association phenomena, which reinforces the methodological value of approaches such as ours. Critically, the authors also explicitly identify the cross-sectional nature of NHANES as a fundamental limitation, calling for longitudinal designs with repeated exposure profiling to delineate critical windows of susceptibility, distinguish chronic accumulation from acute effects, and strengthen causal inference. Our study directly addresses this need serving as an excellent complement to such large-scale exposome mapping efforts. Moreover, by offering open-source atlas of associations, we both converge on a shared vision of the exposome as a replicable, and clinically actionable architecture of environmental risk.

In conclusion, our study points to exposures with potential multisystem impact—such as tobacco smoke, indoor air pollution, dietary quality, and built environment features—that are amenable to intervention and policy action. The atlas (https://outcomewide.isglobal.org/) provides an open platform for hypothesis generation, replication in larger and more diverse populations, and integration with mechanistic studies to strengthen causal inference. Future work should expand exposome-health mapping to additional developmental stages and health outcomes, refine exposure assessment, and test whether modifying identified exposures can shift trajectories of child and adolescent health.

## MATERIALS AND METHODS

### Study population

This study used data from the HELIX cohort, collected during HELIX and ATHLETE (Advancing Tools for Human Early Lifecourse Exposome Research and Translation) projects. The HELIX cohort gathers six established and ongoing longitudinal population-based birth cohorts: Born in Bradford (BiB, UK), Étude des Déterminants pré et postnatals du développement et de la santé de l’Enfant (EDEN, France), Infancia y Medio Ambiente (INMA, Spain), Kaunas Cohort (KANC, Lithuania), The Norwegian Mother, Father and Child Cohort Study (MoBa, Norway) (*28*), and Mother-Child Cohort (RHEA, Greece). Biological sample collection, questionnaire administration, health examinations, and exposure characterization were performed according to identical predefined standardized protocols across cohorts and conducted by trained staff. The full HELIX protocols and databases are described elsewhere (*19*, *27*) and fully detailed in the Supplementary Materials (section S1). From the total 1301 HELIX subjects, the present study included 586 mother-child pairs with available data on environmental exposures assessed during pregnancy and childhood (6 to 11 years) and on clinical and preclinical health outcomes during childhood (6 to 11 years) and adolescence (12 to 18 years) (Fig. 1).

This study was conducted in accordance with the Declaration of Helsinki. All participating families provided written informed consent. Ethical approval for the original cohort studies and the HELIX subcohort examinations was obtained from the following institutional ethics committees: the Bradford Research Ethics Committee (BiB, approval no. 14/YH/0013); the Comité de Protection des Personnes (CPP) Ouest III (EDEN, protocol no. 14.01.02) and the Commission Nationale de l’Informatique et des Libertés (EDEN, request no. 914630); the Comité Ético de Investigación Clínica del Parc de Salut Mar, Barcelona (INMA, approval no. 2005/2106/l); the Lithuania Bioethics Committee (KANC, approval no. 6B-14-38); the Regional Committees for Medical and Health Research Ethics (MoBa, REK reference no. 2013/1628); and the Ethics Committee of the General University Hospital of Heraklion, Crete (RHEA, protocol no. 14694/5-11-2013).

### Exposome assessment

Environmental exposures were assessed in both pregnancy and childhood stages (Fig. 1). A total of 25 families of exposures were assessed in each mother-child pair, with 69 pregnancy and 129 childhood exposures (total of 198 exposures), fully detailed in table S1. Tobacco smoking was assessed based on urine concentrations of cotinine, and via questionnaires for active and secondhand smoking. Exposure to chemical pollutants [organochlorine compounds (OCs), polybrominated diphenyl ethers (PBDEs), perfluoroalkyl substances (PFAS), metals and elements, phthalate metabolites, phenols, and organophosphate (OP) pesticide metabolites] was measured in maternal biological samples collected during pregnancy and in samples collected from the children in the childhood follow-up (*20*, *57*). Outdoor exposures were evaluated using geospatial methods and remote sensing at home and school addresses, encompassing outdoor air pollution, natural spaces, meteorological conditions, built environment measures, and road traffic, during pregnancy and childhood. Lifestyle factors collected via questionnaires included dietary habits during pregnancy and childhood, physical activity, sleep patterns, and pet ownership. Socioeconomic factors during childhood were assessed using the Perceived Stress Scale-4 and the Family Affluence Score, among others (*58*). A complete report on environmental exposure assessment can be found in the Supplementary Materials.

### Health outcomes

We investigated 11 preclinical and clinical health outcomes belonging to four health outcome areas that are known to be linked to noncommunicable disease risk in later life and that represent prevalent health endpoints in European children. Health outcomes were assessed following the same protocols both in the childhood and the adolescence follow-ups, leading to 11 outcome variables at two time points (Fig. 1 and table S11), allowing the examination of exposome-health associations longitudinally. Assessed health domains included the following: (i) mental health (three variables), using neuropsychological and neurobehavioral assessments to derive internalizing and externalizing symptoms scores, namely, the child behavior checklist (CBCL) and the Conner’s Attention Deficit Hyperactivity Disorder (ADHD) index; (ii) the cardiometabolic system (three variables), involving (a) age-and-sex–standardized BMI *z*-scores (*z*-BMI) using the international World Health Organization (WHO) reference curves and (b) blood pressure (systolic and diastolic), assessed following common, standardized protocols, and age- and sex-specific percentiles based on the updated National High Blood Pressure Education Program (NHBPEP) criteria (*59*); (iii) the respiratory system (three variables), including respiratory symptoms (wheeze) and clinical outcomes (doctor diagnosed asthma and use of asthma medication); and (iv) the immune system (two variables), including immunological or allergy-related outcomes (eczema and rhinitis). A detailed report on all assessed health outcomes (protocols, rate of missing values, etc.) is presented in the Supplementary Materials (section S3).

### Covariates

We considered as potential confounders all factors occurring temporally before the measured exposures that might affect the exposures, and at least one of the outcomes of interest. Potential confounders were identified according to previous studies in HELIX for each of the evaluated health outcomes (*10*, *20*–*26*). These included the following: cohort, parental country of birth (both parents native and none or one parent native), maternal education (categorized as low, medium, or high), self-reported prepregnancy maternal BMI (kg/m^2^), maternal age at pregnancy (years), breastfeeding duration (months), parity before cohort pregnancy, the season of conception, paternal education (categorized as low, medium, or high), child’s height, sex and age at outcome assessment follow-ups, nursery attendance in the first 2 years of life, and child’s ethnicity (Caucasian, Asian, and other).

### Statistical analysis

A detailed report on all performed statistical analyses is presented in the Supplementary Materials (section S4). Exposures and covariates were transformed to reach normality, when normality could not be achieved with a transformation, the variable was categorized (table S1). Missing values for all exposures and covariates were imputed using the method of chained equations (*60*), obtaining 20 imputed datasets as described in detail elsewhere (*61*). We followed a two-tiered analysis strategy. First, the dimensionality of the exposome dataset was reduced following recommendations and lessons learned from previous simulation studies (*11*, *12*, *62*, *63*). The ExWAS approach was used for this prefiltering step given its ability to select markers with a high sensitivity (i.e., a low number of false negatives), and Rubin’s rules were used to combine results from the 20 imputed datasets. We used multiple linear regression models for continuous outcome variables and multiple logistic regression models for binary outcomes, adjusted for defined confounders. Exposures showing association with at least two childhood outcomes, or two adolescent outcomes were considered for outcome-wide analysis (fig. S8). The final dimension of exposure datasets for outcome-wide analysis was *P* = 17 for pregnancy exposures and *P* = 56 for childhood exposures (tables S12 and S13).

For outcome-wide analysis, the reduced rank regression (RRR) technique was chosen given its ability to handle the majority of hurdles encountered when working with multiple health domains in the context of the exposome (high dimensionality, multicollinearity, missing values, and mixed-type outcomes) (*17*, *18*). RRR and its multiple variants are a group of dimensionality reduction techniques that assume that all the outcomes and exposures are associated through a shared low-dimensional subspace of latent factors. In RRR, exposome-health associations for each outcome and exposure are individually quantified in the form of regression coefficients (beta estimates, β). Specifically, we applied an extension of RRR called mixed-responses sparse RRR (msRRR), which can accommodate both continuous and binary outcomes and perform feature selection (all beta coefficients for an exposure will be forced to be 0 if the exposure does not have an effect on multiple outcomes simultaneously) [further details can be found in the Supplementary Materials (section S4.4)]. All msRRR analyses were applied separately with the set of pregnancy exposures and the set of childhood exposures, whereas health outcomes from childhood and adolescence were always included under the same model.

The general strategy used with msRRR for outcome-wide analysis is presented in Fig. 1 and fig. S12. Two main approaches were conducted (cross-domains and domain-wise). In the cross-domain approach, all outcomes of interest were modeled together (regardless of the health area they belong to) by including them into the same msRRR model. In domain-wise approaches, a different msRRR model was applied to each group of outcomes (e.g., cardiometabolic, mental health, respiratory, and allergy). Cross-domains and domain-wise approaches offer complementary visions on the relationship between early life exposures and multiple health domains. Although the cross-domain model allows the identification of environmental exposures affecting different health domains simultaneously, domain-wise models permit deepening into associations within each health family, highlighting exposures affecting multiple traits within the same domain. This gave a total of 10 separate msRRR models (Fig. 1). The optimization of model hyperparameters (number of latent factors and penalty term) was done through fivefold cross-validation (CV). The whole population was partitioned into a training set including 80% of participants for model training (hyperparameter optimization through 5-CV) and a test set with the remaining 20% for model evaluation according to predictive performance metrics (fig. S12). All population splits were created ensuring the equidistribution of cohorts and binary outcomes across partitions. For interpretation, new models using identified optimal hyperparameters in the whole population sample were run. In addition, each final model was fitted 500 times in bootstrapped samples with replacement to derive a sign-stability *P* value for each exposure-outcome association, allowing us to evaluate the statistical robustness of each association. More details on the adopted outcome-wide analysis strategy can be found in the Supplementary Materials (sections S4.4 and S4.5).

Last, we performed an extensive LOCO sensitivity analysis, systematically retraining all outcome-wide models by iteratively excluding each of the six participating cohorts. This rigorous computational approach, integrated with bootstrap resampling to derive empirical stability *P* values, ensures that our findings represent robust biological signals rather than artifacts of population-specific heterogeneity. Associations were validated through a dual-layer criterion: directional consistency across most LOCO iterations (>4 of 6-fold) and cross-regional generalizability. We strongly encourage a detailed review of section S4 for more details.

## Supplementary Materials

### This PDF file includes:

Sections S1 to S5 Tables S1 to S18 Figs. S1 to S19 References

## References

[1] P. D. Gluckman, M. A. Hanson, “The developmental origins of health and disease” in *Early Life Origins of Health and Disease* (Springer, 2006), vol. 573, pp. 1–7.

[2] C. Wagner, C. Carmeli, J. Jackisch, M. Kivimäki, B. W. A. van der Linden, S. Cullati, A. Chiolero, Life course epidemiology and public health. Lancet Public Health 9, e261–e269 (2024). 10.1016/S2468-2667(24)00018-5 38553145

[3] R. Vermeulen, E. L. Schymanski, A. L. Barabási, G. W. Miller, The exposome and health: Where chemistry meets biology. Science 367, 392–396 (2020). 10.1126/science.aay3164 PMC7227413 31974245

[4] G. W. Miller, Banbury Exposomics Consortium, Integrating exposomics into biomedicine. Science 388, 356–358 (2025). 10.1126/science.adr0544 PMC12930644 40273259

[5] M. Fang, L. Hu, D. Chen, Y. Guo, J. Liu, C. Lan, J. Gong, B. Wang, Exposome in human health: Utopia or wonderland? Innovation 2, 100172 (2021). 10.1016/j.xinn.2021.100172 PMC8554628 34746906

[6] J. N. Weiss, A. Karma, W. R. MacLellan, M. Deng, C. D. Rau, C. M. Rees, J. Wang, N. Wisniewski, E. Eskin, S. Horvath, Z. Qu, Y. Wang, A. J. Lusis, “Good enough solutions” and the genetics of complex diseases. Circ. Res. 111, 493–504 (2012). 10.1161/CIRCRESAHA.112.269084 PMC3428228 22859671

[7] S. L. Prescott, Early-life environmental determinants of allergic diseases and the wider pandemic of inflammatory noncommunicable diseases. J. Allergy Clin. Immunol. 131, 23–30 (2013). 10.1016/j.jaci.2012.11.019 23265694

[8] GBD 2019 Diseases and Injuries Collaborators, Global burden of 369 diseases and injuries in 204 countries and territories, 1990–2019: A systematic analysis for the Global Burden of Disease Study 2019. Lancet 396, 1204–1222 (2020). 10.1016/S0140-6736(20)30925-9 PMC7567026 33069326

[9] G. Banderali, A. Martelli, M. Landi, F. Moretti, F. Betti, G. Radaelli, C. Lassandro, E. Verduci, Short and long term health effects of parental tobacco smoking during pregnancy and lactation: A descriptive review. J. Transl. Med. 13, 327 (2015). 10.1186/s12967-015-0690-y PMC4608184 26472248

[10] L. Agier, X. Basagaña, L. Maitre, B. Granum, P. K. Bird, M. Casas, B. Oftedal, J. Wright, S. Andrusaityte, M. de Castro, E. Cequier, L. Chatzi, D. Donaire-Gonzalez, R. Grazuleviciene, L. S. Haug, A. K. Sakhi, V. Leventakou, R. McEachan, M. Nieuwenhuijsen, I. Petraviciene, O. Robinson, T. Roumeliotaki, J. Sunyer, I. Tamayo-Uria, C. Thomsen, J. Urquiza, A. Valentin, R. Slama, M. Vrijheid, V. Siroux, Early-life exposome and lung function in children in Europe: An analysis of data from the longitudinal, population-based HELIX cohort. Lancet Planet. Health 3, e81–e92 (2019). 10.1016/S2542-5196(19)30010-5 30737192

[11] S. Santos, L. Maitre, C. Warembourg, L. Agier, L. Richiardi, X. Basagaña, M. Vrijheid, Applying the exposome concept in birth cohort research: A review of statistical approaches. Eur. J. Epidemiol. 35, 193–204 (2020). 10.1007/s10654-020-00625-4 PMC7154018 32221742

[12] C. Warembourg, A. Anguita-Ruiz, V. Siroux, R. Slama, M. Vrijheid, L. Richiardi, X. Basagaña, Statistical approaches to study exposome-health associations in the context of repeated exposure data: A simulation study. Environ. Sci. Technol. 57, 16232–16243 (2023). 10.1021/acs.est.3c04805 PMC10621661 37844068

[13] T. J. VanderWeele, M. B. Mathur, Y. Chen, Outcome-wide longitudinal designs for causal inference: A new template for empirical studies. Statist. Sci. 35, 437–466 (2020).

[14] T. J. Vanderweele, Outcome-wide Epidemiology. Epidemiology 28, 399–402 (2017). 10.1097/EDE.0000000000000641 PMC5378648 28166102

[15] Z. Cao, C. Xu, P. Zhang, Y. Wang, Associations of sedentary time and physical activity with adverse health conditions: Outcome-wide analyses using isotemporal substitution model. EClinicalMedicine 48, 101424 (2022). 10.1016/j.eclinm.2022.101424 PMC9065298 35516443

[16] I. Amine, A. Guillien, C. Philippat, A. Anguita-Ruiz, M. Casas, M. de Castro, A. Dedele, J. Garcia-Aymerich, B. Granum, R. Grazuleviciene, B. Heude, L. S. Haug, J. Julvez, M. López-Vicente, L. Maitre, R. McEachan, M. Nieuwenhuijsen, N. Stratakis, M. Vafeiadi, J. Wright, T. Yang, W. L. Yuan, X. Basagaña, R. Slama, M. Vrijheid, V. Siroux, Environmental exposures in early-life and general health in childhood. Research Square [Preprint] (2023). 10.21203/rs.3.rs-2640215/v1. PMC10360263 37480033

[17] A. Anguita-Ruiz, I. Amine, N. Stratakis, L. Maitre, J. Julvez, J. Urquiza, C. Luo, M. Nieuwenhuijsen, C. Thomsen, R. Grazuleviciene, B. Heude, R. McEachan, M. Vafeiadi, L. Chatzi, J. Wright, T. C. Yang, R. Slama, V. Siroux, M. Vrijheid, X. Basagaña, Beyond the single-outcome approach: A comparison of outcome-wide analysis methods for exposome research. Environ. Int. 182, 108344 (2023). 10.1016/j.envint.2023.108344 PMC12376846 38016387

[18] C. Luo, J. Liang, G. Li, F. Wang, C. Zhang, D. K. Dey, K. Chen, Leveraging mixed and incomplete outcomes via reduced-rank modeling. J. Multivar. Anal. 167, 378–394 (2018).

[19] L. Maitre, J. De Bont, M. Casas, O. Robinson, G. M. Aasvang, L. Agier, S. Andrušaitytė, F. Ballester, X. Basagaña, E. Borràs, C. Brochot, M. Bustamante, A. Carracedo, M. De Castro, A. Dedele, D. Donaire-Gonzalez, X. Estivill, J. Evandt, S. Fossati, L. Giorgis-Allemand, J. R. Gonzalez, B. Granum, R. Grazuleviciene, K. B. Gützkow, L. S. Haug, C. Hernandez-Ferrer, B. Heude, J. Ibarluzea, J. Julvez, M. Karachaliou, H. C. Keun, N. H. Krog, C. H. E. Lau, V. Leventakou, S. Lyon-Caen, C. Manzano, D. Mason, R. McEachan, H. M. Meltzer, I. Petraviciene, J. Quentin, T. Roumeliotaki, E. Sabido, P. J. Saulnier, A. P. Siskos, V. Siroux, J. Sunyer, I. Tamayo, J. Urquiza, M. Vafeiadi, D. Van Gent, M. Vives-Usano, D. Waiblinger, C. Warembourg, L. Chatzi, M. Coen, P. Van Den Hazel, M. J. Nieuwenhuijsen, R. Slama, C. Thomsen, J. Wright, M. Vrijheid, Human Early Life Exposome (HELIX) study: A European population-based exposome cohort. BMJ Open 8, e021311 (2018). 10.1136/bmjopen-2017-021311 PMC6144482 30206078

[20] M. Vrijheid, S. Fossati, L. Maitre, S. Márquez, T. Roumeliotaki, L. Agier, S. Andrusaityte, S. Cadiou, M. Casas, M. de Castro, A. Dedele, D. Donaire-Gonzalez, R. Grazuleviciene, L. S. Haug, R. McEachan, H. M. Meltzer, E. Papadopouplou, O. Robinson, A. K. Sakhi, V. Siroux, J. Sunyer, P. E. Schwarze, I. Tamayo-Uria, J. Urquiza, M. Vafeiadi, A. Valentin, C. Warembourg, J. Wright, M. J. Nieuwenhuijsen, C. Thomsen, X. Basagaña, R. Slama, L. Chatzi, Early-life environmental exposures and childhood obesity: An exposome-wide approach. Environ. Health Perspect. 128, 67009 (2020). 10.1289/EHP5975 PMC7313401 32579081

[21] J. B. Guimbaud, A. P. Siskos, A. K. Sakhi, B. Heude, E. Sabidó, E. Borràs, H. Keun, J. Wright, J. Julvez, J. Urquiza, K. B. Gützkow, L. Chatzi, M. Casas, M. Bustamante, M. Nieuwenhuijsen, M. Vrijheid, M. López-Vicente, M. de Castro Pascual, N. Stratakis, O. Robinson, R. Grazuleviciene, R. Slama, S. Alemany, X. Basagaña, M. Plantevit, R. Cazabet, L. Maitre, Machine learning-based health environmental-clinical risk scores in European children. Commun. Med. 4, 98 (2024). 10.1038/s43856-024-00513-y PMC11116423 38783062

[22] C. Warembourg, L. Maitre, I. Tamayo-Uria, S. Fossati, T. Roumeliotaki, G. M. Aasvang, S. Andrusaityte, M. Casas, E. Cequier, L. Chatzi, A. Dedele, J. R. Gonzalez, R. Gražulevičienė, L. S. Haug, C. Hernandez-Ferrer, B. Heude, M. Karachaliou, N. H. Krog, R. McEachan, M. Nieuwenhuijsen, I. Petraviciene, J. Quentin, O. Robinson, A. K. Sakhi, R. Slama, C. Thomsen, J. Urquiza, M. Vafeiadi, J. West, J. Wright, M. Vrijheid, X. Basagaña, Early-life environmental exposures and blood pressure in children. J. Am. Coll. Cardiol. 74, 1317–1328 (2019). 10.1016/j.jacc.2019.06.069 PMC8713646 31488269

[23] B. Granum, B. Oftedal, L. Agier, V. Siroux, P. Bird, M. Casas, C. Warembourg, J. Wright, L. Chatzi, M. de Castro, D. Donaire, R. Grazuleviciene, L. Småstuen Haug, L. Maitre, O. Robinson, I. Tamayo-Uria, J. Urquiza, M. Nieuwenhuijsen, R. Slama, C. Thomsen, M. Vrijheid, Multiple environmental exposures in early-life and allergy-related outcomes in childhood. Environ. Int. 144, 106038 (2020). 10.1016/j.envint.2020.106038 PMC8768577 32854059

[24] L. Maitre, J. Julvez, M. López-Vicente, C. Warembourg, I. Tamayo-Uria, C. Philippat, K. B. Gützkow, M. Guxens, S. Andrusaityte, X. Basagaña, M. Casas, M. de Castro, L. Chatzi, J. Evandt, J. R. Gonzalez, R. Gražulevičienė, L. Smastuen Haug, B. Heude, C. Hernandez-Ferrer, M. Kampouri, D. Manson, S. Marquez, R. McEachan, M. Nieuwenhuijsen, O. Robinson, R. Slama, C. Thomsen, J. Urquiza, M. Vafeidi, J. Wright, M. Vrijheid, Early-life environmental exposure determinants of child behavior in Europe: A longitudinal, population-based study. Environ. Int. 153, 106523 (2021). 10.1016/j.envint.2021.106523 PMC8140407 33773142

[25] J. Julvez, M. López-Vicente, C. Warembourg, L. Maitre, C. Philippat, K. B. Gützkow, M. Guxens, J. Evandt, S. Andrusaityte, M. Burgaleta, M. Casas, L. Chatzi, M. de Castro, D. Donaire-González, R. Gražulevičienė, C. Hernandez-Ferrer, B. Heude, R. Mceachan, M. Mon-Williams, M. Nieuwenhuijsen, O. Robinson, A. K. Sakhi, N. Sebastian-Galles, R. Slama, J. Sunyer, I. Tamayo-Uria, C. Thomsen, J. Urquiza, M. Vafeiadi, J. Wright, X. Basagaña, M. Vrijheid, Early life multiple exposures and child cognitive function: A multi-centric birth cohort study in six European countries. Environ. Pollut. 284, 117404 (2021). 10.1016/j.envpol.2021.117404 PMC8287594 34077897

[26] N. Stratakis, A. Anguita-Ruiz, L. Fabbri, L. Maitre, J. R. González, S. Andrusaityte, X. Basagaña, E. Borràs, H. C. Keun, L. Chatzi, D. V. Conti, J. Goodrich, R. Grazuleviciene, L. S. Haug, B. Heude, W. L. Yuan, R. McEachan, M. Nieuwenhuijsen, E. Sabidó, R. Slama, C. Thomsen, J. Urquiza, T. Roumeliotaki, M. Vafeiadi, J. Wright, M. Bustamante, M. Vrijheid, Multi-omics architecture of childhood obesity and metabolic dysfunction uncovers biological pathways and prenatal determinants. Nat. Commun. 16, 654 (2025). 10.1038/s41467-025-56013-7 PMC11732992 39809770

[27] M. Vrijheid, X. Basagaña, J. R. Gonzalez, V. W. V. Jaddoe, G. Jensen, H. C. Keun, R. R. C. McEachan, J. Porcel, V. Siroux, M. A. Swertz, C. Thomsen, G. M. Aasvang, S. Andrušaitytė, K. Angeli, D. Avraam, F. Ballester, P. Burton, M. Bustamante, M. Casas, L. Chatzi, C. Chevrier, N. Cingotti, D. Conti, A. Crépet, P. Dadvand, L. Duijts, E. van Enckevort, A. Esplugues, S. Fossati, R. Garlantezec, M. D. G. Roig, R. Grazuleviciene, K. B. Gützkow, M. Guxens, S. Haakma, E. V. S. Hessel, L. Hoyles, E. Hyde, J. Klanova, J. D. van Klaveren, A. Kortenkamp, L. Le Brusquet, I. Leenen, A. Lertxundi, N. Lertxundi, C. Lionis, S. Llop, M. J. Lopez-Espinosa, S. Lyon-Caen, L. Maitre, D. Mason, S. Mathy, E. Mazarico, T. Nawrot, M. Nieuwenhuijsen, R. Ortiz, M. Pedersen, J. Perelló, M. Pérez-Cruz, C. Philippat, P. Piler, C. Pizzi, J. Quentin, L. Richiardi, A. Rodriguez, T. Roumeliotaki, J. M. S. Capote, L. Santiago, S. Santos, A. P. Siskos, K. Strandberg-Larsen, N. Stratakis, J. Sunyer, A. Tenenhaus, M. Vafeiadi, R. C. Wilson, J. Wright, T. Yang, R. Slama, Advancing tools for human early lifecourse exposome research and translation (ATHLETE): Project overview. Environ. Epidemiol. 5, e166 (2021). 10.1097/EE9.0000000000000166 PMC8683140 34934888

[28] P. Magnus, C. Birke, K. Vejrup, A. Haugan, E. Alsaker, A. K. Daltveit, M. Handal, M. Haugen, G. Høiseth, G. P. Knudsen, L. Paltiel, P. Schreuder, K. Tambs, L. Vold, C. Stoltenberg, Cohort profile update: The Norwegian mother and child cohort study (MoBa). Int. J. Epidemiol. 45, 382–388 (2016). 10.1093/ije/dyw029 27063603

[29] M. A. Gomez-Llorente, A. Martínez-Cañavate, N. Chueca, M. A. de la Cruz Rico, R. Romero, A. Anguita-Ruiz, C. M. Aguilera, M. Gil-Campos, M. D. Mesa, B. Khakimov, J. A. Morillo, Á. Gil, J. Camacho, C. Gomez-Llorente, A multi-omics approach reveals new signatures in obese allergic asthmatic children. Biomedicine 8, 359 (2020). 10.3390/biomedicines8090359 PMC7555790 32961859

[30] M. Behl, D. Rao, K. Aagaard, T. L. Davidson, E. D. Levin, T. A. Slotkin, S. Srinivasan, D. Wallinga, M. F. White, V. R. Walker, K. A. Thayer, A. C. Holloway, Evaluation of the association between maternal smoking, childhood obesity, and metabolic disorders: A national toxicology program workshop review. Environ. Health Perspect. 121, 170–180 (2013). 10.1289/ehp.1205404 PMC3569686 23232494

[31] E. Oken, E. B. Levitan, M. W. Gillman, Maternal smoking during pregnancy and child overweight: Systematic review and meta-analysis. Int. J. Obes. 32, 201–210 (2007). 10.1038/sj.ijo.0803760 PMC2586944 18278059

[32] F. Rice, K. Langley, C. Woodford, G. Davey Smith, A. Thapar, Identifying the contribution of prenatal risk factors to offspring development and psychopathology: What designs to use and a critique of literature on maternal smoking and stress in pregnancy. Dev. Psychopathol. 30, 1107–1128 (2018). 10.1017/S0954579418000421 30068414

[33] N. Baïz, J. Just, J. Chastang, A. Forhan, B. De Lauzon-Guillain, A. M. Magnier, I. Annesi-Maesano, Maternal diet before and during pregnancy and risk of asthma and allergic rhinitis in children. Allergy Asthma Clin. Immunol. 15, (2019). 10.1186/s13223-019-0353-2 PMC6589169 31285746

[34] L. Chatzi, R. Garcia, T. Roumeliotaki, M. Basterrechea, H. Begiristain, C. Iñiguez, J. Vioque, M. Kogevinas, J. Sunyer, INMA studay group, RHEA study group, Mediterranean diet adherence during pregnancy and risk of wheeze and eczema in the first year of life: INMA (Spain) and RHEA (Greece) mother–child cohort studies. Br. J. Nutr. 110, 2058–2068 (2013). 10.1017/S0007114513001426 23680284

[35] M. Erkkola, B. I. Nwaru, M. Kaila, C. Kronberg-Kippilä, J. Ilonen, O. Simell, R. Veijola, M. Knip, S. M. Virtanen, Risk of asthma and allergic outcomes in the offspring in relation to maternal food consumption during pregnancy: A Finnish birth cohort study. Pediatr. Allergy Immunol. 23, 186–194 (2012). 10.1111/j.1399-3038.2012.01272.x 22432883

[36] K. N. Chong, E. H. Chean, R. A. Zaki, S. H. M. Taib, H. A. Majid, A. K. Ng, K. P. Eg, S. Y. Hng, C. S. Ju, N. Razali, A. M. Nathan, J. A. de Bruyne, Maternal diet during pregnancy and infant respiratory morbidity: A prospective study. Asia Pac. J. Clin. Nutr. 30, 632–642 (2021). 10.6133/apjcn.202112_30(4).0010 34967192

[37] N. Alvarez Zallo, I. Aguinaga-Ontoso, I. Alvarez-Alvarez, B. Marin-Fernandez, F. Guillén-Grima, C. Azcona-San Julián, Influence of the Mediterranean diet during pregnancy in the development of wheezing and eczema in infants in Pamplona, Spain. Allergol. Immunopathol. 46, 9–14 (2018). 10.1016/j.aller.2017.02.009 28629669

[38] L. Chatzi, M. Torrent, I. Romieu, R. Garcia-Esteban, C. Ferrer, J. Vioque, M. Kogevinas, J. Sunyer, Mediterranean diet in pregnancy is protective for wheeze and atopy in childhood. Thorax 63, 507–513 (2008). 10.1136/thx.2007.081745 18198206

[39] J. A. Castro-Rodriguez, M. Ramirez-Hernandez, O. Padilla, R. M. Pacheco-Gonzalez, V. Pérez-Fernández, L. Garcia-Marcos, Effect of foods and Mediterranean diet during pregnancy and first years of life on wheezing, rhinitis and dermatitis in preschoolers. Allergol. Immunopathol. 44, 400–409 (2016). 10.1016/j.aller.2015.12.002 27087566

[40] S. L. Prescott, C. Macaubas, B. J. Holt, T. B. Smallacombe, R. Loh, P. D. Sly, P. G. Holt, Transplacental priming of the human immune system to environmental allergens: Universal skewing of initial T cell responses toward the Th2 cytokine profile. J. Immunol. 160, 4730–4737 (1998). 9590218

[41] A. O’Neil, C. Itsiopoulos, H. Skouteris, R. S. Opie, S. McPhie, B. Hill, F. N. Jacka, Preventing mental health problems in offspring by targeting dietary intake of pregnant women. BMC Med. 12, 208 (2014). 10.1186/s12916-014-0208-0 PMC4231189 25394602

[42] N. Güil-Oumrait, N. Stratakis, L. Maitre, A. Anguita-Ruiz, J. Urquiza, L. Fabbri, X. Basagaña, B. Heude, L. S. Haug, A. K. Sakhi, N. Iszatt, H. C. Keun, J. Wright, L. Chatzi, M. Vafeiadi, M. Bustamante, R. Grazuleviciene, S. Andrušaitytė, R. Slama, R. McEachan, M. Casas, M. Vrijheid, Prenatal exposure to chemical mixtures and metabolic syndrome risk in children. JAMA Netw. Open 7, e2412040 (2024). 10.1001/jamanetworkopen.2024.12040 PMC11117089 38780942

[43] N. Stratakis, S. Rock, M. A. La Merrill, M. Saez, O. Robinson, D. Fecht, M. Vrijheid, D. Valvi, D. V. Conti, R. McConnell, V. L. Chatzi, Prenatal exposure to persistent organic pollutants and childhood obesity: A systematic review and meta-analysis of human studies. Obes. Rev. 23, e13383 (2022). 10.1111/obr.13383 PMC9512275 34766696

[44] M. Jerrett, R. McConnell, J. Wolch, R. Chang, C. Lam, G. Dunton, F. Gilliland, F. Lurmann, T. Islam, K. Berhane, Traffic-related air pollution and obesity formation in children: A longitudinal, multilevel analysis. Environ. Health 13, 49 (2014). 10.1186/1476-069X-13-49 PMC4106205 24913018

[45] R. McConnell, E. Shen, F. D. Gilliland, M. Jerrett, J. Wolch, C. C. Chang, F. Lurmann, K. Berhane, A longitudinal cohort study of body mass index and childhood exposure to secondhand tobacco smoke and air pollution: The Southern California Children’s Health Study. Environ. Health Perspect. 123, 360–366 (2015). 10.1289/ehp.1307031 PMC4384197 25389275

[46] J. S. Kim, T. L. Alderete, Z. Chen, F. Lurmann, E. Rappaport, R. Habre, K. Berhane, F. D. Gilliland, Longitudinal associations of in utero and early life near-roadway air pollution with trajectories of childhood body mass index. Environ. Health 17, 64 (2018). 10.1186/s12940-018-0409-7 PMC6137930 30213262

[47] J. L. Bolton, S. H. Smith, N. C. Huff, M. I. Gilmour, W. M. Foster, R. L. Auten, S. D. Bilbo, Prenatal air pollution exposure induces neuroinflammation and predisposes offspring to weight gain in adulthood in a sex-specific manner. FASEB J. 26, 4743–4754 (2012). 10.1096/fj.12-210989 22815382

[48] X. Xu, Z. Yavar, M. Verdin, Z. Ying, G. Mihai, T. Kampfrath, A. Wang, M. Zhong, M. Lippmann, L. C. Chen, S. Rajagopalan, Q. Sun, Effect of early particulate air pollution exposure on obesity in mice: Role of p47phox. Arterioscler. Thromb. Vasc. Biol. 30, 2518–2527 (2010). 10.1161/ATVBAHA.110.215350 PMC3065931 20864666

[49] Z. Xu, W. Wang, Q. Liu, Z. Li, L. Lei, L. Ren, F. Deng, X. Guo, S. Wu, Association between gaseous air pollutants and biomarkers of systemic inflammation: A systematic review and meta-analysis. Environ. Pollut. 292, 118336 (2022). 10.1016/j.envpol.2021.118336 34634403

[50] C. R. Davis, J. M. Hodgson, R. Woodman, J. Bryan, C. Wilson, K. J. Murphy, A Mediterranean diet lowers blood pressure and improves endothelial function: Results from the MedLey randomized intervention trial. Am. J. Clin. Nutr. 105, 1305–1313 (2017). 10.3945/ajcn.116.146803 28424187

[51] A. A. Bjerregaard, T. I. Halldorsson, I. Tetens, S. F. Olsen, Mother’s dietary quality during pregnancy and offspring’s dietary quality in adolescence: Follow-up from a national birth cohort study of 19,582 mother-offspring pairs. PLOS Med. 16, e1002911 (2019). 10.1371/journal.pmed.1002911 PMC6742222 31513597

[52] M. La Merrill, C. Emond, M. J. Kim, J. P. Antignac, B. Le Bizec, K. Clément, L. S. Birnbaum, R. Barouki, Toxicological function of adipose tissue: Focus on persistent organic pollutants. Environ. Health Perspect. 121, 162–169 (2013). 10.1289/ehp.1205485 PMC3569688 23221922

[53] Z. Fewell, G. Davey Smith, J. A. C. Sterne, The impact of residual and unmeasured confounding in epidemiologic studies: A simulation study. Am. J. Epidemiol. 166, 646–655 (2007). 10.1093/aje/kwm165 17615092

[54] C. J. Patel, J. P. A. Ioannidis, A. K. Manrai, An atlas of exposome–phenome associations in health and disease risk. Nat. Med. 32, 1501–1510 (2026). 10.1038/s41591-026-04266-0 PMC13099396 41851348

[55] F. Perrier, L. Giorgis-Allemand, R. Slama, C. Philippat, Within-subject pooling of biological samples to reduce exposure misclassification in biomarker-based studies. Epidemiology 27, 378–388 (2016). 10.1097/EDE.0000000000000460 PMC4820663 27035688

[56] M. Casas, X. Basagaña, A. K. Sakhi, L. S. Haug, C. Philippat, B. Granum, C. B. Manzano-Salgado, C. Brochot, F. Zeman, J. de Bont, S. Andrusaityte, L. Chatzi, D. Donaire-Gonzalez, L. Giorgis-Allemand, J. R. Gonzalez, E. Gracia-Lavedan, R. Grazuleviciene, M. Kampouri, S. Lyon-Caen, P. Pañella, I. Petraviciene, O. Robinson, J. Urquiza, M. Vafeiadi, C. Vernet, D. Waiblinger, J. Wright, C. Thomsen, R. Slama, M. Vrijheid, Variability of urinary concentrations of non-persistent chemicals in pregnant women and school-aged children. Environ. Int. 121, 561–573 (2018). 10.1016/j.envint.2018.09.046 30300814

[57] L. S. Haug, A. K. Sakhi, E. Cequier, M. Casas, L. Maitre, X. Basagana, S. Andrusaityte, G. Chalkiadaki, L. Chatzi, M. Coen, J. de Bont, A. Dedele, J. Ferrand, R. Grazuleviciene, J. R. Gonzalez, K. B. Gutzkow, H. Keun, R. McEachan, H. M. Meltzer, I. Petraviciene, O. Robinson, P. J. Saulnier, R. Slama, J. Sunyer, J. Urquiza, M. Vafeiadi, J. Wright, M. Vrijheid, C. Thomsen, In-utero and childhood chemical exposome in six European mother-child cohorts. Environ. Int. 121, 751–763 (2018). 10.1016/j.envint.2018.09.056 30326459

[58] W. Boyce, T. Torsheim, C. Currie, A. Zambon, The family affluence scale as a measure of national wealth: Validation of an adolescent self-report measure. Soc. Indic. Res. 78, 473–487 (2006).

[59] J. T. Flynn, D. C. Kaelber, C. M. Baker-Smith, D. Blowey, A. E. Carroll, S. R. Daniels, S. D. De Ferranti, J. M. Dionne, B. Falkner, S. K. Flinn, S. S. Gidding, C. Goodwin, M. G. Leu, M. E. Powers, C. Rea, J. Samuels, M. Simase, V. V. Thaker, E. M. Urbina, Subcommittee on Screening and Management of High Blood Pressure in Children, Clinical practice guideline for screening and management of high blood pressure in children and adolescents. Pediatrics 140, e20171904 (2017). 10.1542/peds.2017-1904 28827377

[60] I. R. White, P. Royston, A. M. Wood, Multiple imputation using chained equations: Issues and guidance for practice. Stat. Med. 30, 377–399 (2011). 10.1002/sim.4067 21225900

[61] I. Tamayo-Uria, L. Maitre, C. Thomsen, M. J. Nieuwenhuijsen, L. Chatzi, V. Siroux, G. M. Aasvang, L. Agier, S. Andrusaityte, M. Casas, M. de Castro, A. Dedele, L. S. Haug, B. Heude, R. Grazuleviciene, K. B. Gutzkow, N. H. Krog, D. Mason, R. R. C. McEachan, H. M. Meltzer, I. Petraviciene, O. Robinson, T. Roumeliotaki, A. K. Sakhi, J. Urquiza, M. Vafeiadi, D. Waiblinger, C. Warembourg, J. Wright, R. Slama, M. Vrijheid, X. Basagaña, The early-life exposome: Description and patterns in six European countries. Environ. Int. 123, 189–200 (2019). 10.1016/j.envint.2018.11.067 PMC9946269 30530161

[62] J. Barrera-Gómez, L. Agier, L. Portengen, M. Chadeau-Hyam, L. Giorgis-Allemand, V. Siroux, O. Robinson, J. Vlaanderen, J. R. González, M. Nieuwenhuijsen, P. Vineis, M. Vrijheid, R. Vermeulen, R. Slama, X. Basagaña, A systematic comparison of statistical methods to detect interactions in exposome-health associations. Environ. Health 16, 74 (2017). 10.1186/s12940-017-0277-6 PMC5513197 28709428

[63] L. Agier, L. Portengen, M. Chadeau-Hyam, X. Basagaña, L. Giorgis-Allemand, V. Siroux, O. Robinson, J. Vlaanderen, J. R. González, M. J. Nieuwenhuijsen, P. Vineis, M. Vrijheid, R. Slama, R. Vermeulen, A systematic comparison of linear regression–based statistical methods to assess exposome-health associations. Environ. Health Perspect. 124, 1848–1856 (2016). 10.1289/EHP172 PMC5132632 27219331

[64] R. Beelen, G. Hoek, E. Pebesma, D. Vienneau, K. de Hoogh, D. J. Briggs, Mapping of background air pollution at a fine spatial scale across the European Union. Sci. Total Environ. 407, 1852–1867 (2009). 10.1016/j.scitotenv.2008.11.048 19152957

[65] J. Cyrys, M. Eeftens, J. Heinrich, C. Ampe, A. Armengaud, R. Beelen, T. Bellander, T. Beregszaszi, M. Birk, G. Cesaroni, M. Cirach, K. de Hoogh, A. De Nazelle, F. de Vocht, C. Declercq, A. Dedele, K. Dimakopoulou, K. Eriksen, C. Galassi, R. Graulevičiene, G. Grivas, O. Gruzieva, A. H. Gustafsson, B. Hoffmann, M. Iakovides, A. Ineichen, U. Krämer, T. Lanki, P. Lozano, C. Madsen, K. Meliefste, L. Modig, A. Mölter, G. Mosler, M. Nieuwenhuijsen, M. Nonnemacher, M. Oldenwening, A. Peters, S. Pontet, N. Probst-Hensch, U. Quass, O. Raaschou-Nielsen, A. Ranzi, D. Sugiri, E. G. Stephanou, P. Taimisto, M. Y. Tsai, É. Vaskövi, S. Villani, M. Wang, B. Brunekreef, G. Hoek, Variation of NO_2_ and NO_x_ concentrations between and within 36 European study areas: Results from the ESCAPE study. Atmos. Environ. 62, 374–390 (2012).

[66] M. Eeftens, R. Beelen, K. De Hoogh, T. Bellander, G. Cesaroni, M. Cirach, C. Declercq, A. Dedele, E. Dons, A. De Nazelle, K. Dimakopoulou, K. Eriksen, G. Falq, P. Fischer, C. Galassi, R. Gražulevičiene, J. Heinrich, B. Hoffmann, M. Jerrett, D. Keidel, M. Korek, T. Lanki, S. Lindley, C. Madsen, A. Mölter, G. Nádor, M. Nieuwenhuijsen, M. Nonnemacher, X. Pedeli, O. Raaschou-Nielsen, E. Patelarou, U. Quass, A. Ranzi, C. Schindler, M. Stempfelet, E. Stephanou, D. Sugiri, M. Y. Tsai, T. Yli-Tuomi, M. J. Varró, D. Vienneau, S. Von Klot, K. Wolf, B. Brunekreef, G. Hoek, Development of land use regression models for PM_2.5_, PM_2.5_ absorbance, PM_10_ and PM_coarse_ in 20 European study areas; Results of the ESCAPE Project. Environ. Sci. Technol. 46, 11195–11205 (2012). 10.1021/es301948k 22963366

[67] M. Eeftens, M. Y. Tsai, C. Ampe, B. Anwander, R. Beelen, T. Bellander, G. Cesaroni, M. Cirach, J. Cyrys, K. de Hoogh, A. De Nazelle, F. de Vocht, C. Declercq, A. Dedele, K. Eriksen, C. Galassi, R. Gražulevičiene, G. Grivas, J. Heinrich, B. Hoffmann, M. Iakovides, A. Ineichen, K. Katsouyanni, M. Korek, U. Krämer, T. Kuhlbusch, T. Lanki, C. Madsen, K. Meliefste, A. Mölter, G. Mosler, M. Nieuwenhuijsen, M. Oldenwening, A. Pennanen, N. Probst-Hensch, U. Quass, O. Raaschou-Nielsen, A. Ranzi, E. Stephanou, D. Sugiri, O. Udvardy, É. Vaskövi, G. Weinmayr, B. Brunekreef, G. Hoek, Spatial variation of PM_2.5_, PM_10_, PM_2.5_ absorbance and PM_coarse_ concentrations between and within 20 European study areas and the relationship with NO_2_—Results of the ESCAPE project. Atmos. Environ. 62, 303–317 (2012).

[68] R. Beelen, G. Hoek, D. Vienneau, M. Eeftens, K. Dimakopoulou, X. Pedeli, M. Y. Tsai, N. Künzli, T. Schikowski, A. Marcon, K. T. Eriksen, O. Raaschou-Nielsen, E. Stephanou, E. Patelarou, T. Lanki, T. Yli-Tuomi, C. Declercq, G. Falq, M. Stempfelet, M. Birk, J. Cyrys, S. von Klot, G. Nádor, M. J. Varró, A. Dedele, R. Gražulevičiene, A. Mölter, S. Lindley, C. Madsen, G. Cesaroni, A. Ranzi, C. Badaloni, B. Hoffmann, M. Nonnemacher, U. Krämer, T. Kuhlbusch, M. Cirach, A. de Nazelle, M. Nieuwenhuijsen, T. Bellander, M. Korek, D. Olsson, M. Strömgren, E. Dons, M. Jerrett, P. Fischer, M. Wang, B. Brunekreef, K. de Hoogh, Development of NO_2_ and NO_x_ land use regression models for estimating air pollution exposure in 36 study areas in Europe—The ESCAPE project. Atmos. Environ. 72, 10–23 (2013).

[69] A. Schembari, K. De Hoogh, M. Pedersen, P. Dadvand, D. Martinez, G. Hoek, E. S. Petherick, J. Wright, M. J. Nieuwenhuijsen, Ambient air pollution and newborn size and adiposity at birth: Differences by maternal ethnicity (the Born in Bradford study cohort). Environ. Health Perspect. 123, 1208–1215 (2015). 10.1289/ehp.1408675 PMC4629735 25978617

[70] M. Wang, R. Beelen, T. Bellander, M. Birk, G. Cesaroni, M. Cirach, J. Cyrys, K. de Hoogh, C. Declercq, K. Dimakopoulou, M. Eeftens, K. T. Eriksen, F. Forastiere, C. Galassi, G. Grivas, J. Heinrich, B. Hoffmann, A. Ineichen, M. Korek, T. Lanki, S. Lindley, L. Modig, A. Mölter, P. Nafstad, M. J. Nieuwenhuijsen, W. Nystad, D. Olsson, O. Raaschou-Nielsen, M. Ragettli, A. Ranzi, M. Stempfelet, D. Sugiri, M. Y. Tsai, O. Udvardy, M. J. Varró, D. Vienneau, G. Weinmayr, K. Wolf, T. Yli-Tuomi, G. Hoek, B. Brunekreef, Performance of multi-city land use regression models for nitrogen dioxide and fine particles. Environ. Health Perspect. 122, 843–849 (2014). 10.1289/ehp.1307271 PMC4123024 24787034

[71] A. Rahmalia, L. Giorgis-Allemand, J. Lepeule, C. Philippat, J. Galineau, A. Hulin, M. A. Charles, R. Slama, EDEN Mother-Child Cohort Study group, Pregnancy exposure to atmospheric pollutants and placental weight: An approach relying on a dispersion model. Environ. Int. 48, 47–55 (2012). 10.1016/j.envint.2012.06.013 22836169

[72] M. J. Nieuwenhuijsen, H. Kruize, C. Gidlow, S. Andrusaityte, J. M. Antó, X. Basagaña, M. Cirach, P. Dadvand, A. Danileviciute, D. Donaire-Gonzalez, J. Garcia, M. Jerrett, M. Jones, J. Julvez, E. Van Kempen, I. Van Kamp, J. Maas, E. Seto, G. Smith, M. Triguero, W. Wendel-Vos, J. Wright, J. Zufferey, P. J. Van Den Hazel, R. Lawrence, R. Grazuleviciene, Positive health effects of the natural outdoor environment in typical populations in different regions in Europe (PHENOTYPE): A study programme protocol. BMJ Open 4, e004951 (2014). 10.1136/bmjopen-2014-004951 PMC3996820 24740979

[73] J. Herring, Measuring Vegetation (NDVI & EVI): Feature Articles (NASA Earth Observatory, 2000).

[74] A. Smargiassi, M. S. Goldberg, C. Plante, M. Fournier, Y. Baudouin, T. Kosatsky, Variation of daily warm season mortality as a function of micro-urban heat islands. J. Epidemiol. Community Health 63, 659–664 (2009). 10.1136/jech.2008.078147 PMC2701553 19366997

[75] D. T. Duncan, J. Aldstadt, J. Whalen, S. J. Melly, S. L. Gortmaker, Validation of walk score for estimating neighborhood walkability: An analysis of four US metropolitan areas. Int. J. Environ. Res. Public Health 8, 4160–4179 (2011). 10.3390/ijerph8114160 PMC3228564 22163200

[76] L. D. Frank, J. F. Sallis, T. L. Conway, J. E. Chapman, B. E. Saelens, W. Bachman, Many pathways from land use to health: Associations between neighborhood walkability and active transportation, body mass index, and air quality. J. Am. Plann. Assoc. 72, 75–87 (2006).

[77] E. Van Nunen, R. Vermeulen, M. Y. Tsai, N. Probst-Hensch, A. Ineichen, M. Davey, M. Imboden, R. Ducret-Stich, A. Naccarati, D. Raffaele, A. Ranzi, C. Ivaldi, C. Galassi, M. Nieuwenhuijsen, A. Curto, D. Donaire-Gonzalez, M. Cirach, L. Chatzi, M. Kampouri, J. Vlaanderen, K. Meliefste, D. Buijtenhuijs, B. Brunekreef, D. Morley, P. Vineis, J. Gulliver, G. Hoek, Land use regression models for ultrafine particles in six European areas. Environ. Sci. Technol. 51, 3336–3345 (2017). 10.1021/acs.est.6b05920 PMC5362744 28244744

[78] C. H. Jeong, E. D. Wagner, V. R. Siebert, S. Anduri, S. D. Richardson, E. J. Daiber, A. B. McKague, M. Kogevinas, C. M. Villanueva, E. H. Goslan, W. Luo, L. M. Isabelle, J. F. Pankow, R. Grazuleviciene, S. Cordier, S. C. Edwards, E. Righi, M. J. Nieuwenhuijsen, M. J. Plewa, Occurrence and toxicity of disinfection byproducts in European drinking waters in relation with the HIWATE epidemiology study. Environ. Sci. Technol. 46, 12120–12128 (2012). 10.1021/es3024226 PMC4790091 22958121

[79] R. B. Smith, S. C. Edwards, N. Best, J. Wright, M. J. Nieuwenhuijsen, M. B. Toledano, Birth weight, ethnicity, and exposure to trihalomethanes and haloacetic acids in drinking water during pregnancy in the Born in Bradford cohort. Environ. Health Perspect. 124, 681–689 (2016). 10.1289/ehp.1409480 PMC4858386 26340797

[80] C. M. Villanueva, E. Gracia-Lavedán, J. Ibarluzea, L. S. Marina, F. Ballester, S. Llop, A. Tardón, M. F. Fernández, C. Freire, F. Goñi, X. Basagaña, M. Kogevinas, J. O. Grimalt, J. Sunyer, INMA (Infancia y Medio Ambiente) Project, Exposure to trihalomethanes through different water uses and birth weight, small for gestational age, and preterm delivery in Spain. Environ. Health Perspect. 119, 1824–1830 (2011). 10.1289/ehp.1002425 PMC3261969 21810554

[81] A. Danileviciute, R. Grazuleviciene, J. Vencloviene, A. Paulauskas, M. J. Nieuwenhuijsen, Exposure to drinking water trihalomethanes and their association with low birth weight and small for gestational age in genetically susceptible women. Int. J. Environ. Res. Public Health 9, 4470–4485 (2012). 10.3390/ijerph9124470 PMC3546772 23222181

[82] I. H. Caspersen, H. E. Kvalem, M. Haugen, A. L. Brantsæter, H. M. Meltzer, J. Alexander, C. Thomsen, M. Frøshaug, N. M. B. Bremnes, S. L. Broadwell, B. Granum, M. Kogevinas, H. K. Knutsen, Determinants of plasma PCB, brominated flame retardants, and organochlorine pesticides in pregnant women and 3 year old children in The Norwegian Mother and Child Cohort Study. Environ. Res. 146, 136–144 (2016). 10.1016/j.envres.2015.12.020 26749444

[83] F. Goñi, R. López, A. Etxeandia, E. Millán, P. Amiano, High throughput method for the determination of organochlorine pesticides and polychlorinated biphenyls in human serum. J. Chromatogr. B Anal. Technol. Biomed. Life Sci. 852, 15–21 (2007). 10.1016/j.jchromb.2006.12.049 17267299

[84] C. B. Manzano-Salgado, M. Casas, M. J. Lopez-Espinosa, F. Ballester, M. Basterrechea, J. O. Grimalt, A. M. Jiménez, T. Kraus, T. Schettgen, J. Sunyer, M. Vrijheid, Transfer of perfluoroalkyl substances from mother to fetus in a Spanish birth cohort. Environ. Res. 142, 471–478 (2015). 10.1016/j.envres.2015.07.020 26257032

[85] S. Poothong, C. Thomsen, J. A. Padilla-Sanchez, E. Papadopoulou, L. S. Haug, Distribution of novel and well-known poly- and perfluoroalkyl substances (PFASs) in human serum, plasma, and whole blood. Environ. Sci. Technol. 51, 13388–13396 (2017). 10.1021/acs.est.7b03299 29056041

[86] I. Rodushkin, M. D. Axelsson, Application of double focusing sector field ICP-MS for multielemental characterization of human hair and nails. Part I. Analytical methodology. Sci. Total Environ. 250, 83–100 (2000). 10.1016/s0048-9697(00)00369-7 10811254

[87] R. Ramon, M. Murcia, X. Aguinagalde, A. Amurrio, S. Llop, J. Ibarluzea, A. Lertxundi, M. Alvarez-Pedrerol, M. Casas, J. Vioque, J. Sunyer, A. Tardon, B. Martinez-Arguelles, F. Ballester, Prenatal mercury exposure in a multicenter cohort study in Spain. Environ. Int. 37, 597–604 (2011). 10.1016/j.envint.2010.12.004 21239061

[88] A. H. Stern, A. E. Smith, An assessment of the cord blood:maternal blood methylmercury ratio: Implications for risk assessment. Environ. Health Perspect. 111, 1465–1470 (2003). 10.1289/ehp.6187 PMC1241648 12948885

[89] M. A. Padilla, M. Elobeid, D. M. Ruden, D. B. Allison, An examination of the association of selected toxic metals with total and central obesity indices: NHANES 99-02. Int. J. Environ. Res. Public Health 7, 3332–3347 (2010). 10.3390/ijerph7093332 PMC2954548 20948927

[90] A. Sabaredzovic, A. K. Sakhi, A. L. Brantsæter, C. Thomsen, Determination of 12 urinary phthalate metabolites in Norwegian pregnant women by core–shell high performance liquid chromatography with on-line solid-phase extraction, column switching and tandem mass spectrometry. J. Chromatogr. B 1002, 343–352 (2015). 10.1016/j.jchromb.2015.08.040 26355271

[91] D. Valvi, N. Monfort, R. Ventura, M. Casas, L. Casas, J. Sunyer, M. Vrijheid, Variability and predictors of urinary phthalate metabolites in Spanish pregnant women. Int. J. Hyg. Environ. Health 218, 220–231 (2015). 10.1016/j.ijheh.2014.11.003 25558797

[92] C. Philippat, M. Mortamais, C. Chevrier, C. Petit, A. M. Calafat, X. Ye, M. J. Silva, C. Brambilla, I. Pin, M. A. Charles, S. Cordier, R. Slama, Exposure to phthalates and phenols during pregnancy and offspring size at birth. Environ. Health Perspect. 120, 464–470 (2012). 10.1289/ehp.1103634 PMC3295340 21900077

[93] E. Cequier, A. K. Sakhi, L. S. Haug, C. Thomsen, Development of an ion-pair liquid chromatography–high resolution mass spectrometry method for determination of organophosphate pesticide metabolites in large-scale biomonitoring studies. J. Chromatogr. A 1454, 32–41 (2016). 10.1016/j.chroma.2016.05.067 27264744

[94] J. J. Aurrekoetxea, M. Murcia, M. Rebagliato, M. J. López, A. M. Castilla, L. Santa-Marina, M. Guxens, A. Fernández-Somoano, M. Espada, A. Lertxundi, A. Tardón, F. Ballester, Determinants of self-reported smoking and misclassification during pregnancy, and analysis of optimal cut-off points for urinary cotinine: A cross-sectional study. BMJ Open 3, e002034 (2013). 10.1136/bmjopen-2012-002034 PMC3563144 23355667

[95] J. Sunyer, R. Garcia-Esteban, A. M. Castilla, J. J. Aurrekoetxea, C. Iñiguez, A. Tardón, M. Espada, A. Lertxundi, L. Chatzi, M. Rebagliato, M. Kogevinas, INMA project, Exposure to second-hand smoke and reproductive outcomes depending on maternal asthma. Eur. Respir. J. 40, 371–376 (2012). 10.1183/09031936.00091411 22323568

[96] A. Covaci, S. Voorspoels, C. Thomsen, B. van Bavel, H. Neels, Evaluation of total lipids using enzymatic methods for the normalization of persistent organic pollutant levels in serum. Sci. Total Environ. 366, 361–366 (2006). 10.1016/j.scitotenv.2006.03.006 16624383

[97] E. Grimvall, L. Rylander, P. Nilsson-Ehle, U. Nilsson, U. Strömberg, L. Hagmar, C. Östman, Monitoring of polychlorinated biphenyls in human blood plasma: Methodological developments and influence of age, lactation, and fish consumption. Arch. Environ. Contam. Toxicol. 32, 329–336 (1997). 10.1007/s002449900193 9096084

[98] Sedentary Behaviour Research Network, Letter to the editor: Standardized use of the terms “sedentary” and “sedentary behaviours”. Appl. Physiol. Nutr. Metab. 37, 540–542 (2012). 10.1139/h2012-024 22540258

[99] Y. Liu, M. Wang, J. Villberg, T. Torsheim, J. Tynjälä, Y. Lv, L. Kannas, Reliability and validity of Family Affluence Scale (FAS II) among adolescents in Beijing, China. Child Indic. Res. 5, 235–251 (2011).

[100] Y. Jin, M. J. Hein, J. A. Deddens, C. J. Hines, Analysis of lognormally distributed exposure data with repeated measures and values below the limit of detection using SAS. Ann. Occup. Hyg. 55, 97–112 (2011). 10.1093/annhyg/meq061 21177260

[101] E. A. Stuart, M. Azur, C. Frangakis, P. Leaf, Multiple imputation with large data sets: A case study of the Children’s Mental Health Initiative. Am. J. Epidemiol. 169, 1133–1139 (2009). 10.1093/aje/kwp026 PMC2727238 19318618

[102] S. van Buuren, K. Groothuis-Oudshoorn, mice: Multivariate Imputation by Chained Equations in R. J. Stat. Softw. 45, 1–67 (2011).

[103] L. Chen, J. Z. Huang, Sparse reduced-rank regression for simultaneous dimension reduction and variable selection. J. Am. Stat. Assoc. 107, 1533–1545 (2012).

[104] B. Bodinier, S. Filippi, T. H. Nøst, J. Chiquet, M. Chadeau-Hyam, Automated calibration for stability selection in penalised regression and graphical models. J. R. Stat. Soc. Ser. C Appl. Stat. 72, 1375–1393 (2023). 10.1093/jrsssc/qlad058 PMC10746547 38143734

[105] B. Bodinier, S. Rodrigues, M. Karimi, S. Filippi, J. Chiquet, M. Chadeau-Hyam, Stability selection and consensus clustering in R: The R package sharp. J. Stat. Softw. 112, 1–27 (2025).

[106] J. Koponen, P. Rantakokko, R. Airaksinen, H. Kiviranta, Determination of selected perfluorinated alkyl acids and persistent organic pollutants from a small volume human serum sample relevant for epidemiological studies. J. Chromatogr. A 1309, 48–55 (2013). 10.1016/j.chroma.2013.07.064 23972455

[107] L. S. Haug, C. Thomsen, G. Becher, Time trends and the influence of age and gender on serum concentrations of perfluorinated compounds in archived human samples. Environ. Sci. Technol. 43, 2131–2136 (2009). 10.1021/es802827u 19368225

[108] I. Garali, I. Adanyeguh, F. Ichou, V. Perlbarg, A. Seyer, B. Colsch, I. Moszer, V. Guillemot, A. Durr, F. Mochel, A. Tenenhaus, A strategy for multimodal data integration: Application to biomarkers identification in spinocerebellar ataxia. Brief. Bioinform. 19, 1356–1369 (2018). 10.1093/bib/bbx060 29106465

