# Supplementary material for: The persistent multidomain health effects of the early-life exposome
Source: Sci Adv. 2026 Nov 13;12(46):eaec2089. doi: 10.1126/sciadv.aec2089 (PMC13763833; doi:10.1126/sciadv.aec2089)
Supplement: Supplementary file 1 [file sciadv.aec2089_sm.pdf]

Science Advances
AAAS

Supplementary Materials for

The persistent multidomain health effects of the early-life exposome

Augusto Anguita Ruiz *et al.*

Corresponding author: Augusto Anguita Ruiz, augusto.anguita@isglobal.org

Sci. Adv. **12**, eaec2089 (2026)
DOI: 10.1126/sciadv.aec2089

This PDF file includes:

Sections S1 to S5
Tables S1 to S18
Figs. S1 to S19
References

1. Section I. Exposure assessment methods

The HELIX (Human Early Life Exposome) project is a collaborative project across six established, ongoing, longitudinal population-based birth cohort studies in Europe: Born in Bradford (BiB) in the United Kingdom, Étude des Déterminants pré et postnatals du développement et de la santé de l'Enfant (EDEN) in France, INfancia y Medio Ambiente (INMA) in Spain, Kaunas cohort (KANC) in Lithuania, the Norwegian Mother and Child Cohort (MoBa), and the Rhea Mother Child Cohort in Greece. These cohorts contributed to the HELIX subcohort of mother–child pairs who participated in a common, completely harmonized, follow-up examination between December 2013 and February 2016, when the children were between 6–11 y old, as fully described elsewhere (19). Eligibility criteria for inclusion in the subcohort were: a) age 6–11 y at the time of the visit, with a preference for ages 7–9 y; b) sufficient stored pregnancy blood and urine samples available for analysis of prenatal exposure biomarkers; c) complete address history available from first to last follow-up point; and d) no serious health problems that may affect the performance of the clinical testing or affect the volunteer's safety (e.g., acute respiratory infection). In addition, the selection considered whether data on important covariates (diet, socioeconomic factors) were available. Each cohort selected participants at random from the eligible pool in the entire cohort and invited them to participate in this subcohort until the required number of participants was reached. Our comparison of the subcohort with the entire group of cohorts (19) showed that basic characteristics of the subcohort were somewhat different from those of the entire cohort, probably reflecting selective participation of families in the intensive subcohort follow-up visit and data completeness requirements. The final sample size of the subcohort was 1,301 subjects. Recently, the Advancing Tools for Human Early Lifecourse Exposome Research and Translation (ATHLETE) project has followed-up this cohort into adolescence (at 12–18 years, 7 years after the HELIX visit), to add a prospective data collection time point for exposure, and health outcome data to allow evaluation of longitudinal associations into adolescence. Standardized protocols across the six cohorts repeated the common HELIX protocols. The work was covered by ethics approvals from each cohort, and all participants signed an informed consent form for the specific HELIX work, including clinical examination and biospecimen collection and analysis. The final sample size with available data on environmental exposures assessed during pregnancy and early childhood (6-11 years), and on clinical and preclinical health outcomes during early childhood (6-11 years) and adolescence (12-18 years) was 586 participants.

In this section, we provide the methods used to estimate all exposures included in the exposome for the HELIX subcohort. For the purpose of this document "pregnancy" refers to the period from conception to the day of birth, while "childhood" refers to the period between 6 and 11 years (the

exact range varies among cohorts). Part 1 pertains to outdoor and urban exposures, part 2 to water disinfection by-products and indoor air pollutants, part 3 to contaminant exposure biomarkers, and part 4 to lifestyle and other exposures (tobacco smoke, diet, physical activity, alcohol, allergens, sleep, socio-economic capital). As described in parts 1 to 4 below, more variables were available in the subcohort, but not all were included in the exposome list for main analyses for the following reasons:

- Exposures were not included only if they were missing in two entire cohorts (e.g. some diet variables, phthalate metabolites, phenols and PBDEs in pregnancy)
- Exposures with less than 30 subjects in a category without the possibility to recode were excluded. This was the case for DEDTP in pregnancy and childhood, and DMDTP in pregnancy.
- For sets of variables that measure a very similar exposure and those that exhibit correlations higher than 0.9, only one exposure representative of the group was included in the list of exposome variables. For example, this was the case for different UV variables, noise variables, or variables in the built environment calculated for different spatial buffers.
- High correlations (>0.9) were observed between individual PCBs and individual metabolites of DEHP (Phthalates). Sum variables were also created to combine the individual compounds into one variable.
- Some exposure variables were calculated for different time windows as described below (e.g. atmospheric pollutants average for the month before the sub cohort follow-up visit).

A detailed final list of all exposure variables included in the manuscript can be found in **Supplementary Table 1**. Environmental exposures assessed in pregnancy and childhood stages are presented and grouped by family. This table also includes the original percentage of missing values for each variable in the HELIX population (N=1,301) before imputation and the adopted numerical transformation to achieve normality.

Supplementary Table 1. Summary of all included exposome variables, % of missings in the original dataset, distribution of the untransformed variable, units, and transformation or categorization applied.

Exposure group	Exposure variable name	Missing values (%)	Units	Transformation applied**	Categories
**Pregnancy**					
Atmospheric pollutants	NO_2 pregnancy	8.0	µg/m³	Log	-
	$PM_{2.5}$ pregnancy	2.1	µg/m³	None	-
	PM_{10} pregnancy	9.0	µg/m³	None	-

Exposure group	Exposure variable name	Missing values (%)	Units	Transformation applied**	Categories
	PMabsorbance preg	17.2	μg/m^3	Log	-
Home surrounding natural spaces	Green spaces (300 m)	8.7	-	None	0: No; 1: Yes
	Green spaces size (300 m)	8.2	m^2	Log	-
	Green spaces distance	8.2	meters	Sqrt	-
	NDVI (300 m)	1.7	NDVI value	None	-
	Blue spaces (300 m)	8.7	-	None	0: No; 1: Yes
	Blue spaces distance	8.2	meters	Log	-
Meteorology	Temperature pregnancy	7.8	°C (average)	None	-
	Temperature satellite pregnancy	37	ºC	None	-
	Humidity pregnancy	7.8	%	None	-
	Pressure pregnancy	23.4	bar	None	-
Built Environment	Population density	1.8	people/km^2	Sqrt	-
	Building density 300m	8.3	built m^2/km^2	Sqrt	-
	Connectivity 300m	7.5	n of intersections/km^2	Sqrt	-
	Bus public transport lines 300m	47.7	m/km^2	Dichotomous	0: 0; 1: >0
	Bus public transport stops 300m	13.6	n/km^2	Log	-
	Facility density 300m	4.9	n/km^2	Log	-
	Facility richness 300m	4.9	-	None	-
	Land use 300m	8.7	-	None	-
	Walkability	15.1	-	None	-
Traffic	Major Road traffic load (100m)	1.69	vehicles/day	None	0: No major road; 1: major road
	Total traffic load on all roads 100 m	24.3	vehicles/day	Cube	-
	Traffic density on nearest road	1.5	vehicles / day	Cube	-
	Inverse distance to nearest road	1.6	m^{-1}	Log	-
OCs	DDE	19.4	ng/g lipids	Log2	-
	HCB	19.4	ng/g lipids	Log2	-
	PCB 138	19.4	ng/g lipids	Log2	-
	PCB 153	19.4	ng/g lipids	Log2	-

Exposure group	Exposure variable name	Missing values (%)	Units	Transformation applied**	Categories
	PCB 180	19.4	ng/g lipids	Log2	-
	PCB (sum)	19.4	ng/g lipids	Log2	-
PFASs	PFOA	4.7	µg/L	Log2	-
	PFNA	4.7	µg/L	Log2	-
	PFUNDA	20.7	µg/L	Log2	-
	PFHXS	4.7	µg/L	Log2	-
	PFOS	4.7	µg/L	Log2	-
Metals	Mercury	21.6	µg/L	Log2	-
	Magnesium	35.97	Mg/L	Log2	
	Potassium	35.97	Mg/L	Log2	
	Sodium	35.97	Mg/L	Log2	
	Selenium	35.97	Mg/L	Log2	
	Zinq	35.97	Mg/L	Log2	
OP pesticide metabolites	DMP	17.0	µg/g	Log2	-
	DMTP	16.7	µg/g	Log2	-
	DEP	16.8	µg/g	Log2	-
Tobacco smoking/Alcohol	Maternal smoking (active)	3.2	-	None	0=No;1=Yes
	Maternal smoking (passive)	1.69	-	None	0=No;1=Yes
	Maternal smoking (active and second-hand)	2.8	-	None	no exposure; only passive exposure; active smoker
	Cotinine	16.0	-	None	<18.32 ug/L; 18.4-48.4; >50
	Alcohol intake	5.8	-	None	0: No; 1: Yes
Water DBPs	Water THMs	14.5	µg/L	Log	-
	Water Chloroform	14.5	µg/L	Log	-
	Water Brominated THMs	14.5	µg/L	Log	-
Breastfeeding	Ever breastfeeding	24.6	-	None	0=No;1=Yes
Birth-related	Mode of delivery	16.7	Cesarean	None	0=No;1=Yes
	Gestational age	1.08	-	None	-
Socioeconomic	Sibling position	0.6	-	None	-
	House crowding	0.6	Nº People	None	-
	Working Mother	8.7	-	None	0=No;1=Yes
	Paternal age	13	-	None	-
	Type of residence	25.8	Urban	None	0=No;1=Yes

Exposure group	Exposure variable name	Missing values (%)	Units	Transformation applied**	Categories
Pregnancy clinical	Diabetes	21	-	None	0=No;1=Yes
	Pre-eclampsia	28	-	None	0=No;1=Yes
	Hypertension	14	-	None	0=No;1=Yes
Lifestyle	Meat intake	28.6	Times/week	Tertiles	T1: <6.5 ; T2: 6.5-10.0 ; T3: >10.0
	Fish and seafood intake	27.7	Times/week	Tertiles	T1: <1.9 ; T2: 1.9-4.1 ; T3: >4.1
	Cereals intake	27.4	Times/week	Tertiles	T1: <9.0 ; T2: 9.0-27.3 ; T3: >27.3
**Childhood**					
Atmospheric pollutants	NO_2year	0.5	μg/m³	Log	-
	$PM_{2.5}$ year	0.5	μg/m³	None	-
	PM_{10} year	15.6	μg/m³	None	-
	PMabsorbance year	30.9	μg/m³	Log	-
UV	UV - Vit.D month	0.5	kJ/m²	None	-
Surrounding natural space	Green spaces 300 m - home	8.2	-	None	0: No; 1: Yes
	Green spaces 300m - school	8.9	-	None	0: No; 1: Yes
	NDVI 300 m – home	0.3	NDVI value	None	-
	Green Size - home	8.2	m²	Log	-
	Green Size - School	8.9	m²	Log	-
	Green distance - home	8.2	m	Sqrt	-
	Green distance - home	8.9	m	Sqrt	-
	Blue spaces 300 m – home	8.2	-	None	0: No; 1: Yes
	Blue spaces 300m – school	8.9	-	None	0: No; 1: Yes
	Blue distance - home	8.2	m	Log	-
	Blue size - home	8.2	m²	Log	-
Meteorology	Temperature month	7.6	°C	None	-
	Humidity month	7.6	%	None	-
	Temperature (satellite)	16	°C	None	-
	Temperature month max	38	°C	None	-
	Temperature month min	38	°C	None	-
Built Environment	Population density home	0.2	people/km²	Sqrt	-
	Population density school	0.6	people/km²	Sqrt	-
	Building density 300m - home	11.1	built m²/km²	Sqrt	-

Exposure group	Exposure variable name	Missing values (%)	Units	Transformation applied**	Categories
	Building density 300m - school	8.9	built m^2/km^2	Sqrt	-
	Connectivity density 300 m – home	10.7	n of intersections/ m^2	Log	-
	Facility density 300m - school	0.6	n/km^2	Log	-
	Facility richness 300m – home	0.15	-	None	-
	Facility richness 300m– school	0.6	-	None	-
	Walkability index - school	14.1	-	None	-
	Bus public transport stops 300m - home	13.1	n/km^2	Log	-
	Bus public transport stops 300m - school	13.7	n/km^2	Log	-
	Facility density 300m – home	0.2	n/km^2	Log	-
	Land use 300m - home	8.1	-	None	-
	Land use 300m – school	8.9	-	None	-
	Walkability index - home	14.8	-	None	-
Traffic	Road traffic load (100 m) - home	3.4	Veh / day m	Cube	-
	Road traffic load (100 m) - school	3.5	Veh / day m	Cube	-
	Major road in 100m – school	1.8	-	None	0: no major road; 1: major road
	Major road in 100m – home	1.4	-	None	0: no major road; 1: major road
	Inverse distance to nearest road – school	1.8	m -1	Log	-
	Inverse distance to nearest road –home	1.4	m -1	Log	-
OCs	DDE	1.7	ng/g lipids	Log2	-
	DDT	1.7	ng/g lipids	Log2	-
	HCB	1.7	ng/g lipids	Log2	-
	PCB 118	1.7	ng/g lipids	Log2	-
	PCB 138	1.7	ng/g lipids	Log2	-
	PCB 153	1.7	ng/g lipids	Log2	-
	PCB 170	1.7	ng/g lipids	Log2	-

Exposure group	Exposure variable name	Missing values (%)	Units	Transformation applied**	Categories
	PCB 180	1.7	ng/g lipids	Log2	-
	PCBs (sum)	1.7	ng/g lipids	Log2	-
PBDEs	PBDE 47	1.7	ng/g lipids	Log2	-
PFASs	PFOA	0.0	µg/L	Log2	-
	PFNA	0.0	µg/L	Log2	-
	PFHXS	0.0	µg/L	Log2	-
	PFOS	0.0	µg/L	Log2	-
Metals	Arsenic	0.2	µg/L	Log2	-
	Cadmium	0.2	µg/L	Log2	-
	Cobalt	0.2	µg/L	Log2	-
	Cesium	0.2	µg/L	Log2	-
	Copper	0.2	µg/L	Log2	-
	Mercury	0.2	µg/L	Log2	-
	Manganese	0.2	µg/L	Log2	-
	Molybdenum	0.2	µg/L	Log2	-
	Lead	0.2	µg/L	Log2	-
	Potassium	0.2	Mg/L	Log2	-
	Magnesium	0.2	Mg/L	Log2	-
	Sodium	0.2	Mg/L	Log2	-
	Selenium	0.2	Mg/L	Log2	-
	Zinq	0.2	Mg/L	Log2	-
Phthalate metabolites	MEP	0.0	µg/g	Log2	-
	MiBP	0.0	µg/g	Log2	-
	MnBP	0.0	µg/g	Log2	-
	MBzP	0.1	µg/g	Log2	-
	MEHP	3.2	µg/g	Log2	-
	MEHHP	0.2	µg/g	Log2	-
	MEOHP	0.1	µg/g	Log2	-
	MECPP	0.1	µg/g	Log2	-
	DEHP sum (molar sum of MEHP, MEHHP, MEOHP and MECPP)	0.1	µg/g	Log2	-
	OH-MiNP	0.0	µg/g	Log2	-
	oxo-MiNP	0.0	µg/g	Log2	-
Phenols	MEPA	0.2	µg/g	Log2	-

Exposure group	Exposure variable name	Missing values (%)	Units	Transformation applied**	Categories
	ETPA	0.2	µg/g	Log2	-
	BPA	0.9	µg/g	Log2	-
	BUPA	0.4	µg/g	Log2	-
	OXBE	0.0	µg/g	Log2	-
	TRCS	0.0	µg/g	Log2	-
OP pesticide metabolites	DMTP	0.1	µg/g	Log2	-
	DEP	0.2	µg/g	Log2	-
Tobacco smoking	Parental smoking	1.2	-	None	1: neither; 2: one; 3: both
	Second-hand smoking	2.3	-	None	0: no exposure; 1: exposure
Indoor air	Indoor NO_2	11.5	µg/m³	Log	-
	Indoor BTEX	10.6	µg/m³	Log	-
	Indoor benzene	6.3	µg/m³	Log	-
	Indoor $PM_{2.5}$	6.1	µg/m³	Log	-
	Indoor PMabsorbance	6.8	µg/m³	Log	-
Lifestyle	Meat intake	0.9	Times/week	Tertile	T1: <6.0 ; T2: 6.0-9.0 ; T3: >9.0
	Processed meat intake	0.9	Times/week	Tertile	T1: <1.5 ; T2: 1.5-4.0 ; T3: >4.0
	Fish and seafood intake	0.9	Times/week	Tertile	T1: <1.5 ; T2: 1.5-3.0 ; T3: >3.0
	Vegetables intake	1.0	Times/week	Tertile	T1: <6.0 ; T2: 6.0-8.5 ; T3: >8.5
	Fruits intake	1.1	Times/week	Tertile	T1: <7.0 ; T2: 7.0-14.1 ; T3: >14.1
	Dairy products intake	0.9	Times/week	Tertile	T1: <14.6 ; T2: 14.6-25.6 ; T3: >25.6
	Breakfast cereals intake	0.9	Times/week	Tertile	T1: <1.1 ; T2: 1.1-5.5 ; T3: >5.5
	Cereals intake	1.1	Times/week	Tertile	T1: <14.1 ; T2: 14.1-23.6 ; T3: >23.6
	Fast Food intake	1.1	Times/week	Tertile	T1: <0.1 ; T2: 0.1-0.5 ; T3: >0.5

Exposure group	Exposure variable name	Missing values (%)	Units	Transformation applied**	Categories
	Yogurt intake	0.9	Times/week	Tertile	T1: <3.0 ; T2: 3.0-6.5 ; T3: >6.5
	Added fat intake	0.9	Times/week	Tertile	T1: <3.0 ; T2: 3.0-7.0 ; T3: >7.0
	Potatoes intake	1.0	Times/week	Tertile	T1: <3.0 ; T2: 3.0-4.0 ; T3: >4.0
	Bread intake	1.0	Times/week	Tertile	T1: <7.0 ; T2: 7.0-17.5 ; T3: >17.5
	Sweets intake	1.0	Times/week	Tertile	T1: <4.1 ; T2: 4.1-8.5 ; T3: >8.5
	Bakery products intake	0.9	Times/week	Tertile	T1: <2.0 ; T2: 2.0-6.0 ; T3: >6.0
	Soda intake	1.0	Times/week	Tertile	T1: <0.1 ; T2: 0.1-1.0 ; T3: >1.0
	Caffeinated drinks	1.2	Times/week	Tertile	T1: =0 ; T2: =0.13 ; T3: >0.13
	Organic food intake	1.1	Times/week	Tertile	T1: <0.1 ; T2: 0.1-1.0 ; T3: >1.0
	Ready-made food intake	1.1	Times/week	Tertile	T1: <0.1 ; T2: 0.1-0.5 ; T3: >0.5
	KIDMED score	0.8	-	None	-
	Moderate and vigorous PA	20.4	Min/day	None	-
	Sedentary behaviour	1.4	Min/day	None	-
	Sleep duration	0.8	Hours	None	-
Allergens	Mould at home	0.7	-	None	0: No; 1: Yes
	Other pets at home	0.7	-	None	0: No; 1: Yes
	Cat at home	0.2	-	None	0: No; 1: Yes
	Dog at home	0.2	-	None	0: No; 1: Yes
Socio-eco capital	Family affluence score	0.6	-	None	Low; Middle; High
	Contact with family and friends	1.5	-	None	1: (almost) Daily; 2: Once a week; 3: Less than once a week

Exposure group	Exposure variable name	Missing values (%)	Units	Transformation applied**	Categories
	WIFI at home	0.8	-	None	0: No; 1: Yes
	Smartphone	0.6	-	None	0: No; 1: Yes
	House crowding	0.6	People	None	-
Stress	Mother's perceived child's stress	0.69	-	None	-

** Transformations applied to the original variables because of skewed distributions: Log = natural logarithm; Log2=base 2 logarithm; Sqrt=square root; Dichotomous=dichotomized; Tertile=creation of 3 categories based on tertile cut off points; Cube=cubic root.*

1.1. Outdoor and urban exposures

Outdoor and urban exposures were assessed in the following exposure groups: Atmospheric pollutants, ultraviolet (UV) radiation, surrounding natural space, meteorological measures, built environment, traffic, and road traffic noise. Exposure assessment for these exposure groups was conducted within the PostgreSQL (copyright © 1996-2017 The PostgreSQL Global Development Group), PostGIS (Creative Commons Attribution-Share Alike 3.0 License http://postgis.net) and QGIS (QGIS Development Team, 2016. QGIS Geographic Information System) platforms. Source of data for each exposure is summarized in **Supplementary Table 2**. For the pregnancy period, exposure was assessed at the geocoded residential address of each woman. For each woman, assessment of exposure during pregnancy at the geocoded residential address at recruitment was made. For the childhood period, exposure was assessed at the geocoded residential and school addresses of each child as reported at the time of the subcohort visit. In case of multiple addresses, results were averaged by mother or child.

Supplementary Table 2. Exposure data sources

Exposure	BiB	EDEN	INMA	KANC	MoBa	Rhea
Atmospheric pollutants						
NO_2	ESCAPE local LUR	Local dispersion model	ESCAPE local LUR	ESCAPE local LUR	ESCAPE local LUR	ESCAPE local LUR
$PM_{2.5}$	ESCAPE local LUR	ESCAPE European LUR	ESCAPE local LUR	ESCAPE local LUR	ESCAPE local LUR	ESCAPE local LUR
PM_{10}	ESCAPE local LUR	Local dispersion model[a]	ESCAPE local LUR	ESCAPE local LUR	ESCAPE local LUR	ESCAPE local LUR

PM_{abs}	ESCAPE<br><br>local LUR	NA	ESCAPE<br><br>local LUR	ESCAPE<br><br>local LUR	ESCAPE<br><br>local LUR	ESCAPE<br><br>local LUR
Surrounding natural space						
Major green and blue spaces and landuse	Urbanatlas<br><br>(2006)	Urbanatlas<br><br>(2006)	Urbanatlas (2006)	Urbanatlas (2006)	Kartverket (2014)	Urbanatlas (2006)
NDVI	Landsat 4–5 TM, Landsat 7 ETM+, and Landsat 8 OLI/TIRS					
Meteorological measures						
Temperature, Humidity, Pressure[b]	Keighley	Poitiers	Cerdanyola/ Sabadell	Kaunas	Tryvannshogda	Iraklion
UV	TEMIS project	TEMIS project	TEMIS project	TEMIS project	TEMIS project	TEMIS project
Built environment						
Building Density	MasterMap (Ordinance Survey) (2013)	IGN (2014)	ICC (2011)	Open Street Maps (2014)	Open Street Maps (2014)	Greek Statistical Authority (2001)
Street Connectivity and Facilities	Navteq	Navteq	Navteq	Navteq	Navteq	Navteq
Population Density	EEA (2001)	EEA (2001)	INE (2011)	EEA (2001)	Statistics Norway (2005-2013)	EEA (2001)
Public transport (bus stops)	Bradford Metropolitan District Council (2014, 2015)	Grand Poitiers (2013)	Sabadell Municipality (2014)	Open Street Maps (2015)	Company “Ruter” (2015)	Open Street Maps (2015)
Road traffic						
Traffic	City of Bradford metropolitan district, Leeds City Council (2009, 2012)	Atmo Poitou Charentes (2005)	GENCAT (2007)	AudriusDedelé, Vytautas Magnus University (2010)	Municipality of Oslo, Norwegian Public Roads Administration (2006, 2011, 2014)	Fieldwork (2015
Road traffic noise						

Noise	DEFRA GOV. UK (2006)	Mairie de Poitiers (2007-2009)	GENCAT, Barcelona municipality (2006, 2012)	Kaunas Municipality (2007)	Oslo Municipality (2006, 2011)	Fieldwork (2015)

Abbreviations: **DEFRA**, Department of Environment Food and Rural Affairs; **EEA**, European Environment Agency; **ESCAPE**, European Study of Cohorts for Air Pollution Effects; **ETM+**, Enhanced Thematic Mapper Plus; **GENCAT**, Generalitat of Catalonia; **ICC**, Institut Cartogràfic de Catalunya; **IGN**, Institut National de l'Information Géographique et Forestière (http://professionnels.ign.fr); **INE**, Instituto Nacional de Estadística; **LUR**, Land Use Regression; **NA**, not available; **Navteq**: ESRI Street Map for Mobile Navteq 2012; **NDVI**, Normalized Difference Vegetation Index; **NO_2**, nitrogen dioxide; **OLI**, Operational Land Imager; **$PM_{2.5}$**, particulate matter with an aerodynamic diameter of less than 2.5 µm; **PM_{10}**, particulate matter with an aerodynamic diameter of less than 10µm; **PM_{abs}**, absorbance of $PM_{2.5}$ filters; **TEMIS**: Tropospheric Emission Monitoring Internet Service (https://www.temis.nl/uvradiation/UVarchive.php); **TIRS**, Therma Infrared Sensor; **TM**, Thematic Mapper; **UV**, ultraviolet.

[a] only for pregnancy period; [b]location of weather station.

1.1.1. Atmospheric pollutants

The following atmospheric pollutants were assessed: nitrogen dioxide (NO_2), particulate matter with an aerodynamic diameter of less than 2.5 µm ($PM_{2.5}$) and less than 10 µm (PM_{10}), and absorbance of $PM_{2.5}$ filters (PM_{abs}). These were assessed using land use regression (LUR) or dispersion models, temporally adjusted to measurements made in local background monitoring stations and averaged over the periods of interest. In most cases, we used site-specific LUR models developed in the context of the European Study of Cohorts for Air Pollution Effects (ESCAPE) project (64–68). For BiB, assessment for $PM_{2.5}$ and PM_{10} was made based on the ESCAPE LUR model developed in London/Oxford (UK) and adjusted for background PM levels from monitoring stations in Bradford (69). For EDEN, the ESCAPE European-wide LUR model was applied for $PM_{2.5}$ (70), and dispersion models were used to assess NO_2 and PM_{10} exposure (the latter only for the pregnancy period) (71). Data on daily background concentrations of air pollutants for temporal adjustment were obtained from routine background stations active during the whole study period. Back-extrapolation based on other available pollutants was used when data on a pollutant were not available. In particular, daily PM_{10} was used to adjust NO_2; daily NO_2 or PM_{10} factors to adjust $PM_{2.5}$; daily NO_2 to adjust PM_{10}; and daily NO_x to adjust PM_{abs}. Data availability is summarized in **Supplementary Table 3**. For the pregnancy period the exposure estimates were calculated for the three pregnancy trimesters and as the mean of whole pregnancy period. For this manuscript, the **pregnancy period** was selected as the main exposure variable. For childhood period, exposure calculated as the average over one day, one week and one year before the date of the subcohort follow-up examination of the child; this was done for the home address and school address. The **day and the year before examination at home address** were selected as the main exposures for this manuscript.

Supplementary Table 3. Availability of daily values for each outdoor air pollutant by cohort

Cohort	NO_2	PM_{10}	$PM_{2.5}$	PM_{abs}
MoBa	Daily values available	Daily values available	Daily values available	Back extrapolated (NO_x)
KANC	Daily values available	Daily values available	Back extrapolated (NO_2)	Back extrapolated (NO_x)
BiB	Daily values available	Back extrapolated (NO_2)	Back extrapolated (NO_2)	Back extrapolated (NO_x)
EDEN	Daily values available	Back extrapolated (NO_2)	Back extrapolated (NO_2)	NA
INMA	Daily values available	Back extrapolated (NO_2)	Back extrapolated (NO_2)	Back extrapolated (NO_x)
RHEA	Back extrapolated (PM_{10})	Daily values available	Back extrapolated (PM_{10})	NA

Abbreviations: **NA**, not available; **NO_2**, nitrogen dioxide; **$PM_{2.5}$**, particulate matter with an aerodynamic diameter of less than 2.5 µm; **PM_{10}**, particulate matter with an aerodynamic diameter of less than 10µm**;** **PM_{abs}**, absorbance of $PM_{2.5}$ filters.

1.1.2. Surrounding natural spaces

We followed the PHENOTYPE protocol (72) to measure the surrounding greenness, i.e. trees, shrubs and parkland, and applied the Normalized Difference Vegetation Index (NDVI) (73) derived from the Landsat 4–5 Thematic Mapper (TM), Landsat 7 Enhanced Thematic Mapper Plus (ETM+), and Landsat 8 Operational Land Imager (OLI)/Thermal Infrared Sensor (TIRS) with 30m × 30m resolution (courtesy of the U.S. Geology Survey). NDVI quantifies greenness by measuring the difference between near-infrared (which vegetation strongly reflects) and red light (which vegetation absorbs). NDVI values range from +1.0 to -1.0, with higher numbers indicating more greenness. To achieve maximum exposure contrast, we used available cloud-free Landsat images during the period between May and August for years relevant to our period of study and calculated greenness within 100, 300 and 500 meter buffers around each address. Furthermore, an indicator for “residential proximity to major green spaces” was created, as it covers different aspects of natural space exposure, i.e. easy access to recreational space. We calculated access to major green spaces (parks or countryside) and major blue spaces (bodies of water) as the straight line distance from the home or school to nearest blue or green space with an area greater than 5000 m^2 from topographical maps (74) or local sources, see table **Supplementary Table 2**. We also created a dichotomous variable to define whether a major green or blue space was present or not within a buffer of 300 m. For the pregnancy period the **presence of a major blue or green space, and NDVI** within a 300 meter buffer, were selected as the main exposure variables. For the childhood period, we selected the **presence of a major blue or green space and NDVI** in a 300 meter buffer (at **home** and at **school**).

1.1.3. Meteorological variables

We used meteorological stations in the study area to obtain data on temporal variability in temperature. Daily measurements of temperature and humidity were obtained from a local weather station in each study area and averaged over each period of interest. Atmospheric pressure data were obtained from the ESCAPE project, and were available only for pregnancy trimesters and the entire pregnancy period (**pregnancy mean** was selected as main exposure), not for the childhood period. During the childhood period temperature and humidity were estimated for the home and school address. Daily, weekly and monthly measurements of UV radiation (as erythemal UV, Viamin-D and DNA damaging UV) at home and at school at 0.5 x 0.5 degree resolution were obtained from the Global Ozone Monitoring Experiment onboard the ERS-2 (European Remote Sensing) satellite (Temis), and averaged over the day, week and month before the subcohort follow-up examination. For the childhood period, monthly UV-vit D at home address was selected as main exposure variable. For the pregnancy period, estimates were not available because of measurement errors.

1.1.4. Built environment

Topological maps for the following built environment indicators were obtained from local authorities or from Europe wide sources (**Supplementary Table 2**). Building density was calculated within 100 and 300 meters buffer by dividing the area of building cover (m^2) by the area of each buffer (km^2). Population density was calculated as the number of inhabitants per km^2 surrounding the home address. Street connectivity was calculated as the number of street intersections inside 100 and 300 meters buffer, divided by the area (km^2) of each buffer. Facility richness index was calculated as the number of different facility types present divided by the maximum potential number of facility types specified, in a buffer of 300 meters, giving a score of 0 to 1. Facility density index was calculated as the number of facilities present divided by the area of the 300 meters buffer (number of facilities/km^2). A higher value indicates a more availability of different facility types. Landuse Shannon's Evenness Index (SEI) was calculated to provide the proportional abundance of each type of land use in a buffer of 300 meters, giving a score between 0 and 1. It was calculated by multiplying each proportion of land use type by its logarithm and dividing the sum of all land use type products by the logarithm of the total possible land use types. We developed an indicator of walkability, adapted from the previous walkability indexes (75, 76), calculated as the mean and sum of the deciles of population density, street connectivity, facility richness index and land use SEI within 300 meters buffers, giving a walkability score ranging from 0 to 1. Accessibility was measured by BST (bus public transport)

lines and stops were obtained from local authorities of each study area and from Open Street Maps ("OpenStreetMap") where local layers were not available. BST lines density was calculated as meters of BST lines inside 100, 300 and 500 meters buffer, divided by the buffer area in square kilometers. BST stop density was calculated as the number of BST inside 100, 300 and 500 meters buffer, divided by the buffer area in square kilometers. For the current manuscript, we selected the **300 meter buffer at home and school address as** main exposure during pregnancy and childhood periods.

1.1.5. Road traffic

Traffic density indicators (traffic density on nearest road, traffic load on all and major roads within 100 m buffer and inverse distance to nearest road) were calculated from road network maps following the ESCAPE protocol (66, 68). A fieldwork campaign was conducted in Heraklion during 2015, to assess multiple exposures as previously described (77). Briefly, measurements of manual traffic counts of light and heavy vehicles over 15 minutes, and of noise, averaged over 30 minutes monitoring (Sonometer SC160, CESVA monitors -Spain), were made in 160 sites around the city. Sites were chosen representing multiple types (e.g. traffic, urban background, urban green etc.). During the campaign each monitoring site was measured three times in different seasons (summer, winter and autumn). We applied the LUR methods and GIS predictor variables used within the ESCAPE project to develop LUR models of traffic count and road traffic noise (**Supplementary Table 4**). For the analyses in this manuscript, we selected, the total traffic load on major roads in a 100 m buffer (home and school), the total traffic load on all roads in a 100 m buffer (pregnancy and home), traffic density on nearest road (pregnancy and home), and inverse distance to nearest road (pregnancy and home).

Supplementary Table 4. Summary of land use regression models and descriptive statistics of traffic count and road traffic noise exposure within Heraklion.

Exposure	LUR model	R^2 model	R^2 cross validation	RMSE	Moran's I^2 (p value)	Mean Measured levels (range)
Traffic count	1.2 -0.38 * TypeofRoad + PostCode + Land Use – 0.47 * LOG dist dense road + 0.004 * Buffer 50 m to roads	0.71	0.65	3.2veh/ 15 mins	-0.04 (0.16)	133 veh/15 mins [0-933]

Abbreviations: **dB**, decibel; **LUR**, land use regression; mins, minutes; **RMSE**, Root-mean-square deviation; **dist**, distance; **veh**, vehicles.

1.2. Water Disinfection By-Products and Indoor Air Pollutants

1.2.1. Water Disinfection By-Products (DBPs)

We collected data on routine measurements of disinfection by-product (DBP) in water from water companies for all cohorts for the pregnancy period. For KANC, BiB, INMA, and RHEA cohorts this was built on the HiWate project (Health Impacts of long-term exposure to disinfection by-products in drinking Water) (78) that previously modeled exposure levels in the water supply of the residence of each participating mother-child pair. For BiB, routine monitoring data on trihalomethanes (THMs) were obtained for the eight water supply zones covering the study area. Each zone was sampled nine times per year on average, giving 374 data points in total (79). For INMA, levels of THMs were ascertained based on sampling campaigns and regulatory data from local authorities and water companies. Sampling locations were defined to be geographically representative of the study areas, and water samples were collected from taps with no filtration or other treatments that could affect THMs concentration. THMs were determined in 198 places (80). For RHEA, the city was divided into six zones according to the source of underground water used in each area, corresponding to six different water treatment plants. In total, 18 sampling points were selected (12 areas in Heraklion and 6 in rural areas), which covered geographically the residences of participating mother-child pairs. For KANC, tap water THM concentration, derived as the average of quarterly sample values over the time that the pregnancy occurred from all sampling sites located in each distribution system, and geocoded maternal address at birth to assign the individual women's residential exposure index (81). Routine DBP measurements were acquired for MoBa and EDEN cohorts as these cohorts were not part of the HiWate project. THMs exposure levels were modeled for each residence, following the protocol developed within HiWate (78). We estimated exposure to total THMs, and for chloroform and brominated THMs separately during each pregnancy and in the entire pregnancy; for the current manuscript, the pregnancy averages were used.

1.2.2. Indoor Air Pollutants

Indoor air concentrations of nitrogen dioxide (NO_2), particulate matter <2.5µm ($PM_{2.5}$), particulate matter absorbance (PM_{abs}), benzene, and toluene, ethylbenzene, xylene (TEX) were estimated through a prediction model that combined measurements in the homes of a subgroup of children with questionnaire data from the subcohort. Measurements of indoor NO_2, benzene and TEX were conducted in the homes of 157 participants as part of the child panel study , which was nested within the HELIX subcohort in all cohorts except MoBa. $PM_{2.5}$ and PM_{Abs} were measured in INMA, BiB, and EDEN. Participants in the child panel study were followed for one week in two seasons, and the last day of the first week coincided with the subcohort examination, including the completion the main HELIX questionnaire. NO2, benzene and TEX sampling lasted

7 days, and $PM_{2.5}$ and PM_{Abs} sampling lasted 24 hours. NO_2 short-term diffusive Passam samplers were used to measure indoor NO_2 concentrations. The samplers were composed of polypropylene housing with a 20 mm diameter opening, covered with a removable plastic cap and protected from wind disturbance by a teflon membrane. Triethanolamine was used as absorbent material inside the tube. NO_2 was collected by molecular diffusion to the absorbent and its concentration was determined spectrophotometrically by the Saltzmann method. The detection limit (DL) for a week's sampling for the NO_2 sampler was 0.3 µg/m^3. Passam ORSA5 diffusion tubes were used to measure indoor levels of benzene, toluene, ethylbenzene and ortho-, para- and metaxylenes. The DL for a week's sampling for each compound was 0.4 µg/m^3. The samplers were placed in the living rooms of the participating homes, away from the sources of ventilation. After collection, the NO_2, Benzene and TEX samplers were hermetically sealed and kept in zip-lock bags in boxes, in a cool and dark place and shipped to the analyzing laboratory within 3 months of the end of the sampling campaign.

For indoor PM modeling, active $PM_{2.5}$ cyclone pumps were placed in the living room. After 24 hours the samples were collected and sent to the laboratory. $PM_{2.5}$ mass was collected gravimetrically using 37-mm Teflon filters held in a cyclone (model GK2.05 SH, BGI Inc., Waltham MA, USA) with an aerodynamic cut point of 2.5 µm and connected to a BGI/Mesa Labs A4004 pump working at 3.5L/min. Filter weighing and reflectance measurements were conducted with a microbalance of 1 µg accuracy (Model MX5, Mettler-Toledo International Inc., Switzerland) and a Smoke Stain Reflectometer (SSR) (Model 43D, Diffusion Systems Ltd., UK), respectively. Measurement procedures, quality control, as well as $PM_{2.5}$ mass concentration and absorbance estimations followed the ESCAPE project protocols (both available at https://research-portal.uu.nl/en/publications/development-of-no2-and-nox-land-use-regression-models-for-estimat/).

Statistical analyses were performed separately for each of the exposure variables. A TEX variable was created by summing the concentrations of each TEX compound. The HELIX main questionnaire was used to identify housing and participant characteristics as input for the prediction model; these characteristics included: exposure to environmental tobacco smoke, cooking and heating methods at the home, cleaning products among others.

After extracting potential predictor variables from the questionnaires, bivariate analyses were run by either Kruskal-Wallis or Wilcoxon rank sum tests, as all of the potential predictors were categorical and the exposure variables were not normally distributed. The variables that yielded a p-value lower than 0.2 in bivariate analyses were selected to enter into the multiple linear regression models. Prior to that, univariate linear regressions were performed for each of the predictors selected in the bivariate analysis in order to assess the adjusted determination coefficient (adjusted R^2) for each of them individually. To ensure normality of the distributions

of the outcome variables, the univariate linear regression models and subsequent multiple linear regression models were built using log-transformed.

Supervised forward stepwise procedure was employed to build multiple linear regression models. In all cases the starting point for the regression was the variable which yielded the highest adjusted R^2 in the univariate linear regressions. Then the other predictors were added one-by-one and additional increase in the adjusted R^2 was recorded. The variable which increased the adjusted R^2 by a highest value was retained in the model and the procedure was repeated until none of the variables increased the adjusted R^2 by at least 1%. In case any of the variables included into the model had individual p value equal or higher than 0.05, it was removed from the model. All statistical analyses were performed using R Statistic Software (version 3.4.1).

The best explained pollutant was NO_2 with an R^2 of 57%, followed by PM_{Abs} with 50%. **Supplementary Table 5** shows the efficiency of the models and the statistically significant variables. For example, cohort, natural gas oven, type of hob and boiler, butane in the living room, and the number of people living in the house, were the statistically significant variables in the NO_2 model; all of these were positively correlated. Prediction models were then use to estimate these 5 indoor air pollutants in the entire subcohort.

Supplementary Table 5. **Summary of the models of indoor air pollution. *** < 0.001 ** < 0.005 * < 0.05. For negative coefficients (-) sign is included.**

Exposure	NO_2	Benzene	TEX	PM2.5	PMAbs
**Explained variability (R^2)**	**57%**	**31%**	**31%**	**47%**	**50%**
Cohort	***			*	**
Oven with natural gas	***				
Type of hob	***				
Type of boiler	**				
Butane in living room	**			*	
How many people live at home?	***				
Garage connected to the house?		***	*		
$PM_{2.5}$ outdoor		***			
Does air pollution bother you?		*			
Does your family manage financially?		**			
Number of floors of the house		(-)*			

How many cigarettes per week smoke (mother)?	*			
How often you use degreasing sprays?		**		
Presence of central heating?		(-)*		
How often use perfumed cleaning products?		(-)**		
How many cigarettes smoke (mother's partner)?		**		
Calendar month		**		
NO_2 outdoor		*		***
How many cigarettes last week (mother)?			***	***
Family has a car?			(-)**	
Stay at home parent?			**	
How often use glass cleaning sprays?			*	

1.3. Contaminant exposure biomarkers

For the totality of children in the subcohort (1,301), the determinations of a set of chemical contaminants (organochlorine compounds (OCs), polybrominated diphenyl ethers (PBDEs), perfluoroalkyl substances (PFAS), metals and elements, phthalate metabolites, phenols, and organophosphate (OP) pesticide metabolites) were performed at the Department of Environmental Exposure and Epidemiology at the Norwegian Institute of Public Health (NIPH), in Norway or in collaboration with their contract laboratories as described in detail by Haug et al. (57). This was also the case for the majority of the maternal samples collected during pregnancy or at birth and stored in cohort biobanks; however, for some maternal samples in some cohorts, measurements were already completed at thus we used these results (**Supplementary Table 6**).

Supplementary Table 6. Concentrations of chemical contaminants previously analyzed in other labs

	Total maternal samples analyzed	Analyzed in NIPH as part of HELIX	Previously analyzed in other labs

Organochlorine compounds	1078	657	INMA: 223<br>RHEA: 198
Polybrominated diphenyl ethers	855	657	RHEA: 198<br>(only PBDE-47 available)
Perfluoroalkyl substances	1240	1032	INMA: 208
Metals and essential elements	1020	833	INMA: 223<br>(only Hg available)
Phthalate metabolites	1089	914	INMA: 175
Phenols	1085	1023	EDEN: 62
Organophosphate pesticide metabolites	1086	1086	-
Cotinine	1093	883	INMA: 210
Creatinine	1093	870	INMA: 223
Lipids	1075	654	INMA: 223<br>RHEA: 198

1.3.1. Quality assurance

The sample collections for the children were performed in a completely harmonized way, using the same protocols and equipment for sample collection and processing in all six cohorts. The children's samples were randomized into batches before chemical analyses, aiming at a minimum of three cohorts to be included in each batch. However, this was not feasible for the maternal samples as the cohorts shipped the maternal samples at different time points to the laboratories for analysis.

1.3.2. Chemical analysis

Supplementary Table 7 shows the fifty-eight environmental chemicals measured in the HELIX subcohort. **Supplementary Tables 8** and **9** show the collection time points and the biological matrices, respectively.

Supplementary Table 7. Chemical contaminants and number of samples analyzed from mothers and children in the HELIX subcohort.

Compound		Children<br><br>% quantifiable samples	Maternal<br><br>% quantifiable samples
**Organochlorine compounds (OCs)**			
2,3',4,4',5-Pentachlorobiphenyl	PCB 118	99.8	79.1
2,2',3,4,4',5'-Hexachlorobiphenyl	PCB 138	99.8	96.5
2,2',4,4',5,5'-Hexachlorobiphenyl	PCB 153	100	99.6
2,2',3,3',4,4',5-Heptachlorobiphenyl	PCB 170	90.7	99.5
2,2',3,4,4',5,5'-Heptachlorobiphenyl	PCB 180	99.2	97.6
4,4'dichlorodiphenyltrichloroethane	DDT	79.8	65.6
4,4'dichlorodiphenyldichloroethylene	DDE	100.0	99.9
Hexachlorobenzene	HCB	99.9	99.1
**Polybrominated diphenyl ethers (PBDEs)**			
2,2',4,4'-Tetrabromodiphenyl ether	PBDE 47	90.8	80.9
2,2',4,4',5,5'-Hexabromodiphenyl ether	PBDE 153	54.4	72.9
**Perfluoroalkyl substances (PFASs)**			
Perfluorohexanesulfonate	PFHxS	99.7	97.5
Perfluorooctanesulfonate	PFOS	99.8	100
Perfluorooctanoate	PFOA	100	99.7
Perfluorononanoate	PFNA	99.5	97.9
Perfluoroundecanoate	PFUnDA	68.6	95.4
**Metals and essential elements**			
Mercury	Hg	97.7	98.9
Cadmium	Cd	86.5	99.6
Lead	Pb	100	100
Arsenic	As	67.1	58.5
Cesium	Cs	100	100
Copper	Cu	100	100
Thallium	Tl	7.2	1.1
Manganese	Mn	100	100
Zinc	Zn	100	100

Cobalt	Co	99.9	100
Molybdenum	Mo	99.5	100
Sodium	Na	100	100
Potassium	K	100	100
Magnesium	Mg	100	100
**Phthalate metabolites**			
Monoethyl phthalate	MEP	100	99.0
Mono-iso-butyl phthalate	MiBP	100	99.9
Mono-n-butyl phthalate	MnBP	100	100
Mono benzyl phthalate	MBzP	99.9	99.7
Mono-2-ethylhexyl phthalate	MEHP	96.8	99.5
Mono-2-ethyl-5-hydroxyhexyl phthalate	MEHHP	99.8	100
Mono-2-ethyl-5-oxohexyl phthalate	MEOHP	99.9	100
Mono-2-ethyl 5-carboxypentyl phthalate	MECPP	99.9	99.9
Mono-4-methyl-7-hydroxyoctyl phthalate	oh-MiNP	100	92.6
Mono-4-methyl-7-oxooctyl phthalate	oxo-MiNP	100	95.7
**Phenols**			
Methyl paraben	MEPA	99.7	99.8
Ethyl-paraben	ETPA	99.3	97.4
Propyl-paraben	PRPA	67.3	97.3
N-Butyl paraben	BUPA	96.6	97.0
Bisphenol-A	BPA	98.3	99.4
Oxybenzone	OXBE	100	98.5
**Organophosphate (OP) pesticide metabolites**			
Triclosan	TCS	99.9	99.3
Dimethyl phosphate	DMP	49.3	90.8
Dimethyl thiophosphate	DMTP	90.4	88.9
Dimethyl dithiophosphate	DMDTP	18.2	41.6
Diethyl phosphate	DEP	80.9	97.8
Diethyl thiophosphate	DETP	43.5	50.0
Diethyl dithiophosphate	DEDTP	1.5	1.7
**Other compounds**			
Cotinine		17.4	43.7

Creatinine		100	100
Phospholipids		100	62.4
Total cholesterol		100	100
Triglycerides		100	100
High-density lipoprotein cholesterol	HDL	100	100
Low-density lipoprotein cholesterol	LDL	99.8	100

n analysed: samples with biomarker measurements
% quantifiable samples: % of the biomarker measurements with concentrations reported

Supplementary Table 8. Collection time points of maternal and child blood and urine samples (mean, SD)

	Cohort					
	BiB	EDEN	KANC	INMA	MoBa	RHEA
Mother, gestational weeks	26.6 (1.4)	26.1 (1.2)	39.4 (1.3)	13.7 (2.0) / 34.2 (1.3) [a]	18.7 (0.9)	14.1 (3.7)
Child, years	6.6 (0.2)	10.8 (0.6)	6.5 (0.5)	8.8 (0.6)	8.5 (0.5)	6.5 (0.3)

Abbreviations: **SD**: standard deviation
[a]In INMA, blood was collected in the first trimester whereas urine was collected in the third trimester of pregnancy.

Supplementary Table 9. Biological matrices of maternal and child samples

	Cohort					
Chemicals	BiB	EDEN	KANC	INMA	MoBa	RHEA
**OCs and PBDEs**						
Mother	serum/plasma	serum	-	serum	plasma	serum
Child	serum	serum	serum	serum	serum	serum
**PFASs**						
Mother	serum/plasma	serum	whole blood	plasma	plasma	serum
Child	plasma	plasma	plasma	plasma	plasma	plasma
**Metals**						
Mother	whole blood	whole blood	whole blood	cord whole blood	whole blood	whole blood
Child	whole blood	whole blood	whole blood	whole blood	whole blood	whole blood

**Phthalate metabolites, phenols, OP pesticide metabolites, cotinine, and creatinine**						
Mother	urine	urine	-	urine	urine	urine
Child	urine	urine	urine	urine	urine	urine
**Lipids**						
Mother	serum/plasma	serum	-	serum	plasma	serum
Child	plasma	plasma	Plasma	plasma	plasma	plasma

Abbreviations: **OC**: organochlorine; **OP**: organophosphate ; **PBDEs**: polybrominateddiphenyl ethers; **PFASs**: per-fluoroalkyl substances.

1.3.3. Organochlorine compounds (OCs)

Concentrations of OCs were determined in serum or plasma according to Caspersen et al. (82) except that gas chromatography–mass spectrometry (GC-MS/MS) was used instead of gas chromatography/high-resolution mass spectrometry (GC-HRMS). The limit of detection (LOD) was in the range of 0.3 to 1.5 pg/g. OCs concentrations in maternal samples (serum) of INMA and RHEA were determined according to Goñi et al. (83) with a LOD of 67.0 pg/g and Koponen et al. (106) with LODs between 1.7 and 14.3 pg/g, respectively. We also calculated the sum of PCBs by summing the concentrations of the 5 PCBs in pg/g.

1.3.4. Polybriminated diphenylethers (PBDEs)

Concentrations of PBDEs were determined in serum or plasma following the method described in Caspersen et al. (82) also using GC-MS/MS for detection. The LOD ranged from 0.15 to 0.3 pg/g. In RHEA only PBDE-47 was determined in maternal samples (serum) following the method described in Koponen et al. (106) with a LOD of 2.85pg/g.

1.3.5. Perfluoroalkyl substances (PFAS)

Concentrations of PFASs were determined in serum or plasma using the method by Haug et al. (107), while the method by Poothong et al. (85) was applied for the whole blood samples. The LOD was 0.02 µg/L for all PFASs. In the majority of INMA maternal samples (plasma), PFASs were determined according to Manzano-Salgado et al. (84) and with LODs between 0.05 and 0.1 µg/L. Only five maternal samples from INMA were analyzed at NIPH. In order to know whether concentrations measured in both labs were comparable we performed an inter-laboratory comparison of 10 samples with low to high PFOS concentrations as reference selected from all

analyzed in the Institute for Occupational Medicine, RWTH Aachen University (Germany) (84). NIPH was blinded to the concentrations of samples. PFOS and PFHxS plasma concentrations determined in both laboratories were highly correlated (Spearman r=0.83 and 0.93, respectively) whereas PFOA and PFNA were less correlated (Spearman r=0.70 and 0.55, respectively). The three samples with low PFOS concentrations had levels between the LOD and the LOQ or close to the LOQ for PFHxS, PFOA, and PFNA. Considering that concentrations between the LOD and the LOQ have higher uncertainty, we excluded these samples and the spearman correlations became higher: PFOA r=0.96, PFHxS r=0.93, and PFNA r=0.86. Due to the high correlations the NIPH concentrations for subjects included in the comparison have been used. For the PFASs, 1:1 ratios were assumed for serum and plasma, while 1:2 ratios were used for whole blood vs serum/plasma (85). Thus, for PFASs all whole blood concentrations were multiplied by two.

1.3.6. *Metals and essential elements*

Concentrations of 15 metals and elements in whole blood were performed at ALS Scandinavia, Sweden according to Rodushkin et al. (86). The LOD ranged from 0.003–3.03 µg/L except for sodium (Na), potassium (K) and magnesium (Mg) for which the LOD ranges from 0.06-0.15 mg/L. Mercury in INMA was determined in cord whole blood following the procedure described in Ramon et al. (87) with a LOD of 2.0 µg/L. Cord blood Hg concentrations were be divided by 1.7 to be comparable with maternal whole blood concentrations (88, 89)

1.3.7. **Phthalate metabolites**

Concentrations of ten phthalate metabolites were determined in urine according to Sabaredzovic et al. (90). The LOD ranged from 0.06 to 0.61µg/L. In the majority of INMA maternal samples, phthalates were determined according to Valvi et al. (91) with LOD ranged from 0.5-1.0 µg/L except 37 INMA samples that were analyzed at NIPH. For comparability, we analyzed 10 samples with low to high monoethyl phthalate (MEP) concentrations as reference selected from all analyzed in the Bioanalysis Research Group at the Hospital del Mar Medical Research Institute (Barcelona, Spain) (91). NIPH was blinded to the concentrations of samples. Urinary concentrations of the phthalate metabolites determined in both laboratories were highly correlated (Spearman ranging from r=0.69 to 0.97). Due to the high correlations the
NIPH concentrations for subjects included in the comparison have been used. We also calculated the total concentration of di-2-ethylhexyl phthalate (DEHP) by summing the molar concentrations of mono-2-ethylhexyl phthalate (MEHP), mono-2-ethyl-5-hydroxyhexyl phthalate (MEHHP), mono-2-ethyl-5-oxohexyl phthalate (MEOHP), and mono-2-ethyl 5-carboxypentyl phthalate (MECPP). The molar concentrations (in µmol/L) were calculated by dividing the concentration of every metabolite by its molecular weight.

1.3.8. Phenols

Concentrations of phenols were determined in urine according to Güil-Oumrait et al (2024) (42) with the LOD ranging between 0.03-0.06 μg/L. In EDEN, phthalate metabolites were determined in urine samples according to Philippat et al. (92) with the LOD ranged from 0.2-2.3 μg/L. We performed an inter-lab comparison of 12 samples selected from all analyzed in the I National Center for Environmental Health laboratory at the CDC in Atlanta, Georgia, USA (92). NIPH was blinded to the concentrations of samples. Phenols urinary concentrations determined in both laboratories were strongly correlated (Spearman ranging from r=0.90 to 1.0). Due to the high correlations the NIPH concentrations for subjects included in the comparison have been used.

1.3.9. Organophosphate (OP) pesticide metabolites

Analysis of OP pesticide metabolites in urine was made according to Cequier et al. (93) and with the LOD ranged from 0.06-0.36μg/L.

1.3.10. Cotinine

Concentrations of cotinine in urine were determined using The Immulite® 2000 Nicotine Metabolite (Cotinine) 600 Test on an Immulite 2000 XPi from Siemens Healthineers at Fürst Medisinsk Laboratorium, Norway. The LOD was 3.03μg/L. Cotinine in maternal urine samples from INMA were determined according to Aurrekoetxea et al. (94) and with a LOD of 1.21μg/L. We performed an interlab-comparison of 10 urine samples with low to high cotinine concentrations selected from all analyzed in the Public Health Laboratory of Bilbao - LSPPV (Spain) (94). NIPH was blinded to the concentrations of samples. Cotinine urinary concentrations determined in both laboratories were highly correlated (Spearman r=0.95).

For maternal smoking, a categorical variable was created based on the urinary cotinine levels to distinguish non-smokers, second-hand-tobacco smokers, and smokers (95):

- Non-smokers: values <LOD or cotinine levels <18.5 μg/L
- Second-hand-tobacco smokers: cotinine levels ≥18.5-50 μg/L
- Smokers: cotinine levels >50 μg/L

In the children, a categorical variable was created categorizing urinary cotinine levels as detected or not detected considering the limit of detection of 3.03μg/L.

1.3.11. Adjustments for total fat percentage and creatinine

Concentrations of lipids were determined in the Fürst Medical Analysis Laboratory in serum or plasma using the FS kit from DiaSys for phospholipids and the ADVIA® Chemistry XPT System

for the other lipids. LODs ranged from 0.003 to 0.08 mmol/L. In maternal samples (serum) of INMA and RHEA total cholesterol and triglycerides were determined using the Cobas Mira self-analyzer (Roche Diagnostic, Basel, Switzerland) using an enzymatic-colorimetric method with spin react reagents and a standard enzymatic method, respectively. Phospholipid concentrations in maternal samples from INMA and RHEA were calculated based on the formula of (96). Total fat percentage was calculated considering the molecular weight of phospholipids, total cholesterol, and triglycerides, and calculated according to the method described in Grimvall et al. (97). Concentrations of OCs and PBDEs were then adjusted in respect to total fat percentage and expressed in ng/g of lipids. Concentrations of creatinine in urine were performed on an AU680 Chemistry System form Beckman Coulter using DRI® Creatinine-Detect® Test at Fürst Medisinsk Laboratorium, Norway with a LOD of 0,03mmol/L. Creatinine in maternal samples of INMA and EDEN were determined by using the Jaffé method - Beckman Coulter© AU5400 and an enzymatic reaction using a Roche Hitachi 912 chemistry analyzer (Roche Hitachi, Basel, Switzerland), respectively. Urinary concentrations of phthalate metabolites, phenols, OP pesticide metabolites, and cotinine were adjusted in respect to creatinine and expressed in μg/g of creatinine.

1.4. <u>Lifestyle and other exposures</u>

1.4.1. <u>Tobacco smoke</u>

Tobacco smoke exposure was assessed in pregnancy via questionnaire for active and passive smoking, as well as based on cotinine measurements (as described in part 3). Pregnancy questions on tobacco smoke from the cohorts were harmonized as part of the ESCAPE project. Tobacco smoke exposure of the mother at any point during pregnancy was categorised into: no exposure, only passive smoke exposure, active smoking. Active smoking was also measured by the number of cigarettes per day on average during pregnancy

For children, in addition to the cotinine-based classification, the following two variables were created based on the questionnaires completed by the parents:

- The global exposure of the child to ETS with two categories: "no exposure", no exposure at home neither in other places; “exposure”: exposure in at least one place, at home or outside.
- Active smoking of the parents: “1” none of the parents, “2” one parent or “3” both parents.

1.4.2. <u>Diet</u>

Diet during pregnancy was assessed through food frequency questionnaires by each cohort and harmonized *a posteriori* for the HELIX project. Harmonization was possible for eight main food groups (average consumption in times/week) and folic acid supplementation intake (yes/no) in the first trimester for five of the six cohorts (KANC not available) as summarized in **Supplementary Table 10.** Information about breastfeeding duration (in weeks) was collected by the cohorts and then harmonized as part of HELIX. Information on the child's diet was collected through the standardized HELIX subcohort questionnaire. The child's diet was then summarized in 15 food groups (times/week) and dietary habits such as eating organic food (see **Supplementary Table** 10). We also included the KIDMED index, a dietary score representative of healthy eating and based on the principles of Mediterranean dietary patterns. The KIDMED index consists of 16 questions with questions denoting a negative connotation with respect to the Mediterranean diet assigned a value of -1, and those with a positive aspect scored +1. Further, we analyzed as separate variables few factors that contribute to the KIDMED index including fast food visits, organic food and ready-made supermarket meal consumption. All diet variables were categorized in terciles (representing low, moderate and high intake).

Supplementary Table 10. Diet variables included in the exposome for pregnancy and childhood periods

	Pregnancy	Childhood
Cereals	Yes	Yes
Dairy products	-	Yes
Fish and seafood	Yes	Yes
Fruits	-	Yes
Meat	Yes	Yes
Vegetables	-	Yes
Visits a fast food restaurant/take away	-	Yes
Folic acid supplementation (yes/no)	-	-
Legumes	-	-
Breastfeeding duration (in weeks)	-	Yes
Bakery products	-	Yes
Breakfast cereal	-	Yes
Bread (white and whole wheat)	-	Yes
Potatoes	-	Yes
Sweets	-	Yes
Yogurt and probiotics	-	Yes
Processed meat	-	Yes

Total added lipids (butter, margarine and vegetable oils)	-	Yes
Beverages (sodas)	-	Yes
Caffeinated drinks	-	Yes
Organic food	-	Yes
Ready-made supermarket meal	-	Yes
KIDMED score	-	Yes

1.4.3. Physical activity

For children, the moderate-to-vigorous physical activity variable was created based on questionnaire data. It was defined as the amount of time children spent doing physical activities with intensity above 3 metabolic equivalent tasks (METs), and is expressed in units of min/day. Physical activity over-reporting was corrected based on the accelerometer (Actigraph) correlation with questionnaire answers, using the data from three cities involved in the HELIX panels (nested study of the HELIX project where participants wore accelerometers for two non-consecutive weeks).

A variable representing sedentary behavior in the children was created based on the questionnaire and corresponds to the duration of time spent watching TV, playing computer games, or other sedentary games. This variable is a new concept that is commonly defined as ''any waking behavior characterized by an energy expenditure <1.5 metabolic equivalent tasks (METs) while in a sitting or reclining posture'' by the Sedentary Behaviour Research Network (98). Sedentary behavior has been shown to be a health risk factor independently from physical activity.

1.4.4. Alcohol

Alcohol consumption during pregnancy was harmonized based on questionnaire data from the cohorts and classified as whether or not any alcohol was consumed during pregnancy (except in the KANC cohort where the lowest exposure category included women with less than 1 glass a month).

1.4.5. Allergens

For allergens exposure only pet ownership of the child was added to the exposome. There was no prenatal information on this. Three variables were created based on the HELIX questionnaire as follows: (1) if the child had any cats that live mainly in his home (2) or dogs, or (3) any other pets than dogs and cats. The presence of mold at home was assessed by a questionnaire.

1.4.6. Sleep

Sleep duration was available for the subcohort children, not for the mothers, and corresponds to the average sleep duration at night during an entire week (weighted average of weekdays and weekend sleep duration). This variable was calculated based on the questionnaire taking the average bedtime and wake-up time (earliest and latest bedtime/wake-up times available) during weekdays and weekends.

1.4.7. Socio-economic capital

Questions related to socio-economic position (maternal education and others) were collected during the pregnancy in all cohorts and harmonized for use as covariates in analyses; they were not included in the exposome as separate exposure variables. In the childhood exposome, the Family Affluence Score (FAS) was included based on questions from the subcohort questionnaire (99). A composite FAS score was calculated based on the responses to the next four items: (1) Does your family own a car, van or truck? (2) Do you have your own bedroom for yourself? (3) During the past 12 months, how many times did you travel away on holiday with your family? (4) How many computers does your family own? (Liu et al. (99)). A three point ordinal scale was used, where FAS low (score 0,1,2) indicates low affluence, FAS medium (score 3,4,5) indicates middle affluence, and FAS high (score 6,7,8,9) indicates high affluence FAS (58). The FAS score in this study had only a maximum value of 7 instead of 9 because of the smaller number of possible answers for certain items.

Further social capital-related questions were included in the HELIX questionnaire to capture different aspects of social capital, relating both to the cognitive (feelings about relationships) and structural (number of friends, number of organizations) dimensions and to bonding capital (close friends and family), bridging capital (neighborhood connections, looser ties) and linking capital (ties across power levels; for example, political membership). Two summary variables were selected for the exposome analysis: social participation (membership of organizations: 0, 1, or 2) and contact with friends and family (daily, once a week, less than once a week). In addition, house crowding was included, representing the number of persons living in the house with the child.

2. Section II. Health outcomes

The selection of health outcome domains was guided by the strategic framework of the Human Early Life Exposome (HELIX) project (19) and prioritized based on their public health relevance as major non-communicable disease (NCD) end-points. Following the 'Developmental Origins of Health and Disease' (DOHaD) paradigm, these systems (cardiometabolic, respiratory,

neurodevelopment & mental, and immune health) are considered particularly sensitive to environmental insults during critical prenatal and early-childhood developmental windows.

Health outcomes were assessed during both the childhood and adolescence follow-ups, enabling a longitudinal examination of exposome-health associations. The initially considered areas encompassed: 1) Mental health and cognition, we performed neuropsychological and neurobehavioral assessments calculating internalizing and externalizing symptoms scores, using the child behavior checklist (CBCL), and the Conner's Attention Deficit Hyperactivity Disorder (ADHD) index. Cognitive domains were fluid intelligence (Raven's Coloured Progressive Matrices test, CPM), attention (Attention Network Test, ANT) and working memory (N-Back task); 2) The cardiometabolic system, we assessed height and weight and calculated BMI age-and-sex–standardized z-scores (z-BMI) using the international World Health Organization (WHO) reference curves. Blood pressure (BP) was also assessed following common, standardized protocols, and age- and sex-specific percentiles were constructed according to the updated National High Blood Pressure Education Program (NHBPEP) criteria; 3) The respiratory system, we assessed respiratory symptoms (e.g., wheeze), clinical outcomes (e.g., doctor diagnoses of asthma), and lung function (forced vital capacity (FVC), and forced expiratory volume in 1 s percent predicted values (FEV_1%)) assessed through a spirometry test; and 4) The immune system, focusing on allergy-related outcomes (e.g., dermatitis, eczema, rhinitis).

To ensure statistical power and longitudinal design for our outcome-wide models, specific exclusion criteria were applied. First, we focused on outcomes measured across follow-ups. Second, only outcomes with a missing data rate <30% at both childhood and adolescence stages were selected for the final analysis (**Supplementary Figure 1**). Ultimately, 11 clinical and preclinical outcomes were included in the study (**Supplementary Table 11**) spanning the following domains (cardiometabolic, respiratory, mental, and immune health), with their distributions presented in **Supplementary Figure 2**.

Supplementary Table 11. List of health parameters (outcomes) studied in outcome-wide analysis

Cardiometabolic health	Mental health	Respiratory health	Allergy/Immune System
**- Systolic Blood Pressure Z-Score**<br>**- Diastolic Blood Pressure Z-Score**	- ADHD index (DSM-IV)<br>- Internalizing index (CBCL)	- Diagnosis of Asthma (ever)<br>- Use of Asthma medication in the last 12 months	- Dermatitis in the last 12 months<br>- Rhinitis in the last 12 months

**- Body Mass Index Z-Score**	- Externalizing index (CBCL)	- Wheezing in the last 12 months

Each outcome has been assessed twice (in early childhood and adolescence follow-ups). Abbreviations ADHD: Attention Deficit Hyperactivity Disorders, CBCL: Child Behaviour Checklist.

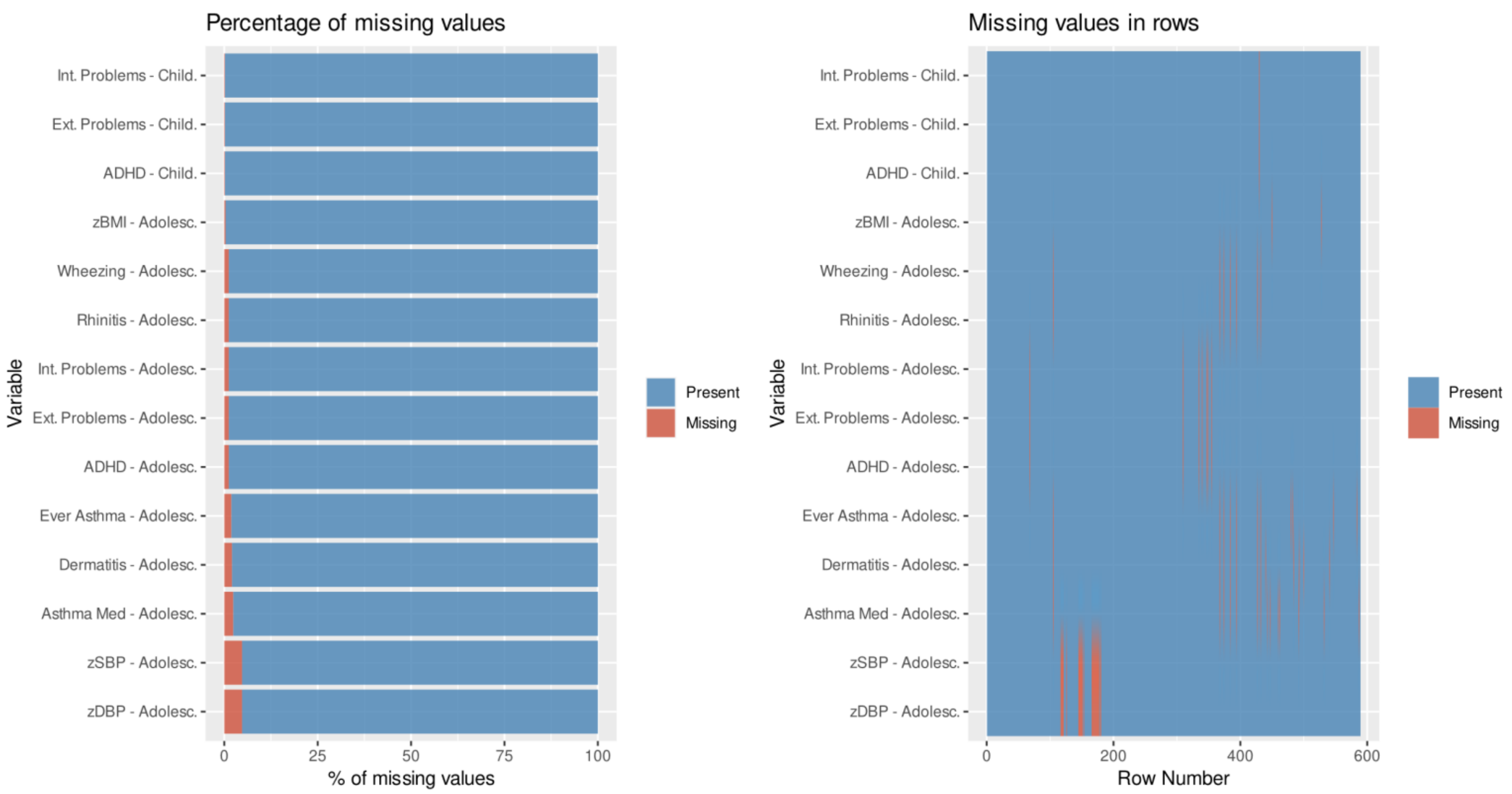

Supplementary Figure 1. Missing patterns of health outcomes at both childhood and adolescence stages.

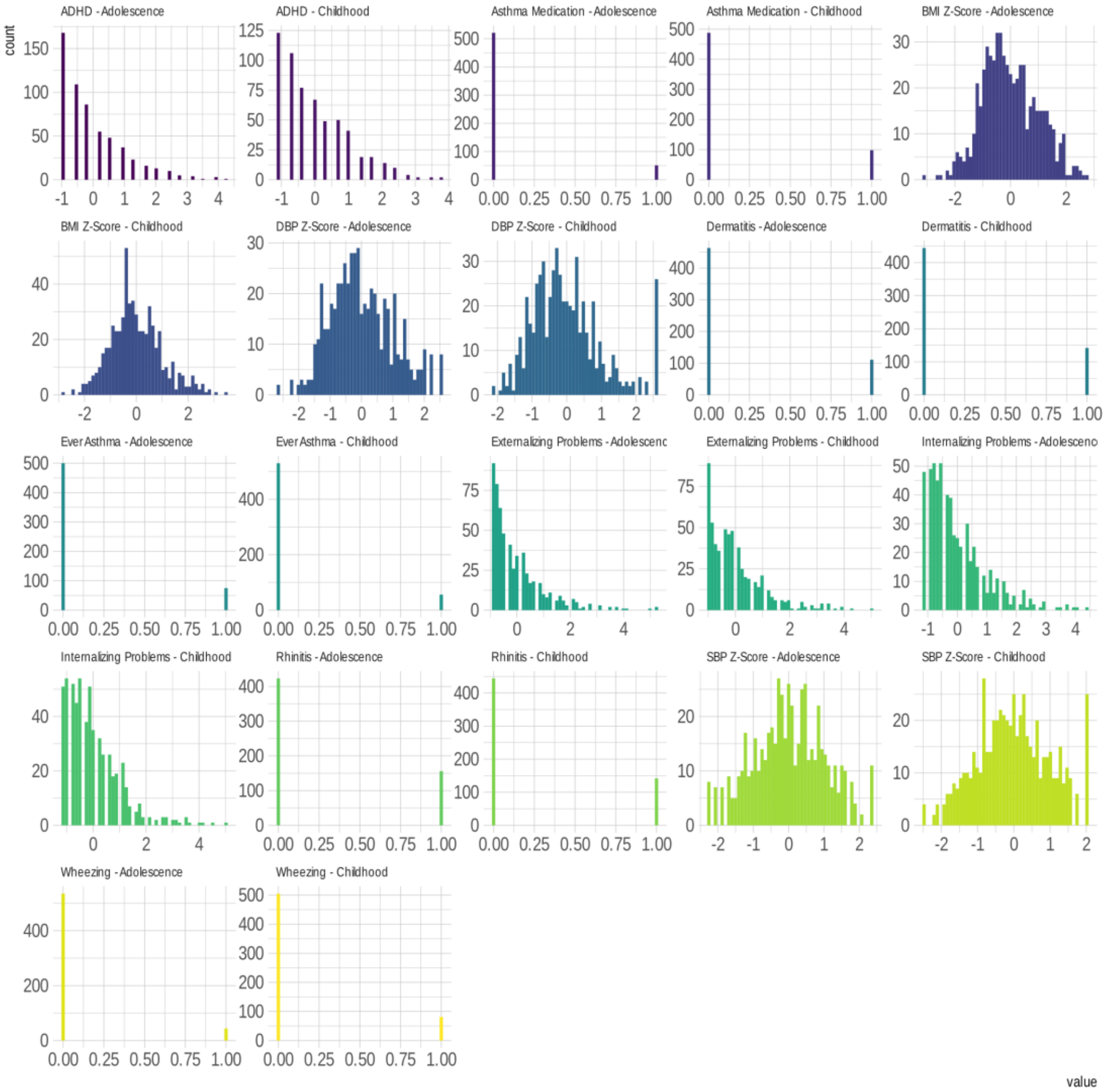

Supplementary Figure 2. Histograms of health outcomes at both childhood and adolescence stages. *Binary outcomes (asthma medication, ever asthma, dermatitis, rhinitis and wheezing) were recoded from Yes/No to 0*

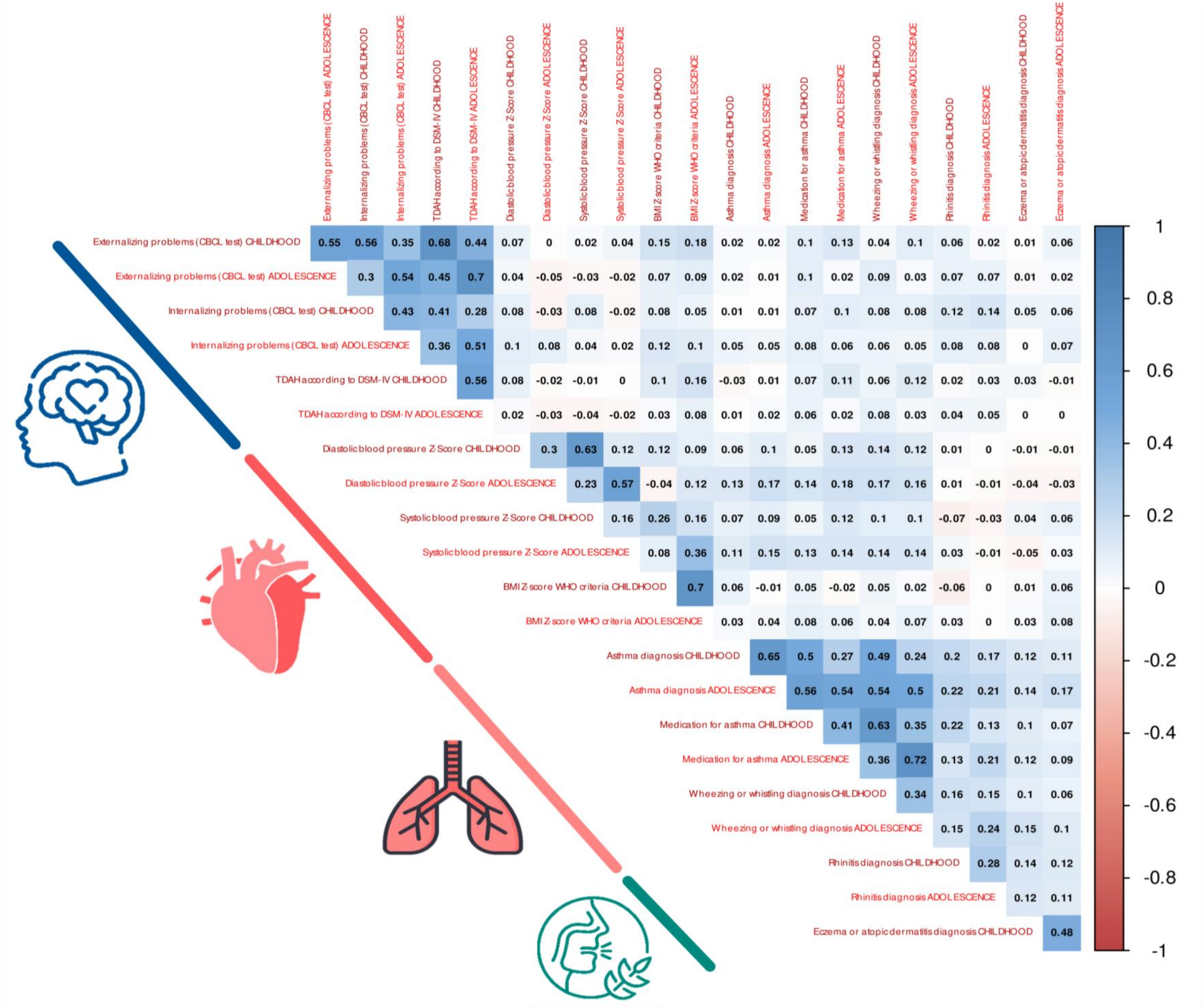

Supplementary Figure 3. Health outcomes correlation heatmap. *The plot includes clinical and preclinical health outcomes assessed both at childhood and adolescent follow-ups.*

3. Section III. Confounders

The strategy adopted for confounding control involved considering as potential confounders all factors occurring temporally prior to the measured exposures that might affect the exposures, and at least one of the outcomes of interest. Potential confounders were identified according to previous individual research conducted in HELIX for each of the evaluated health outcomes. These included; cohort, if parents are natives of the country from which they were recruited, maternal education (categorized as low, medium, or high), pre-pregnancy maternal BMI, maternal age, breastfeeding duration, parity before pregnancy, trimester of conception, paternal education (categorized as low, medium, or high), child's height sex and age at outcome assessment follow-ups, nursery attendance in the first two years of life, and child's ethnicity (Caucasian, Pakistani or Asian, and other). We also considered pubertal status as a potential confounder (evaluated using the Tanner scale through images and questionnaires). Tanner was finally not included as an adjusting covariate given its high correlation with child's age at the adolescence follow-up (model stability problems), and since findings were not altered after its removal. Histograms and correlation plots for identified confounders are presented in **Supplementary Figure 4** and **5**.

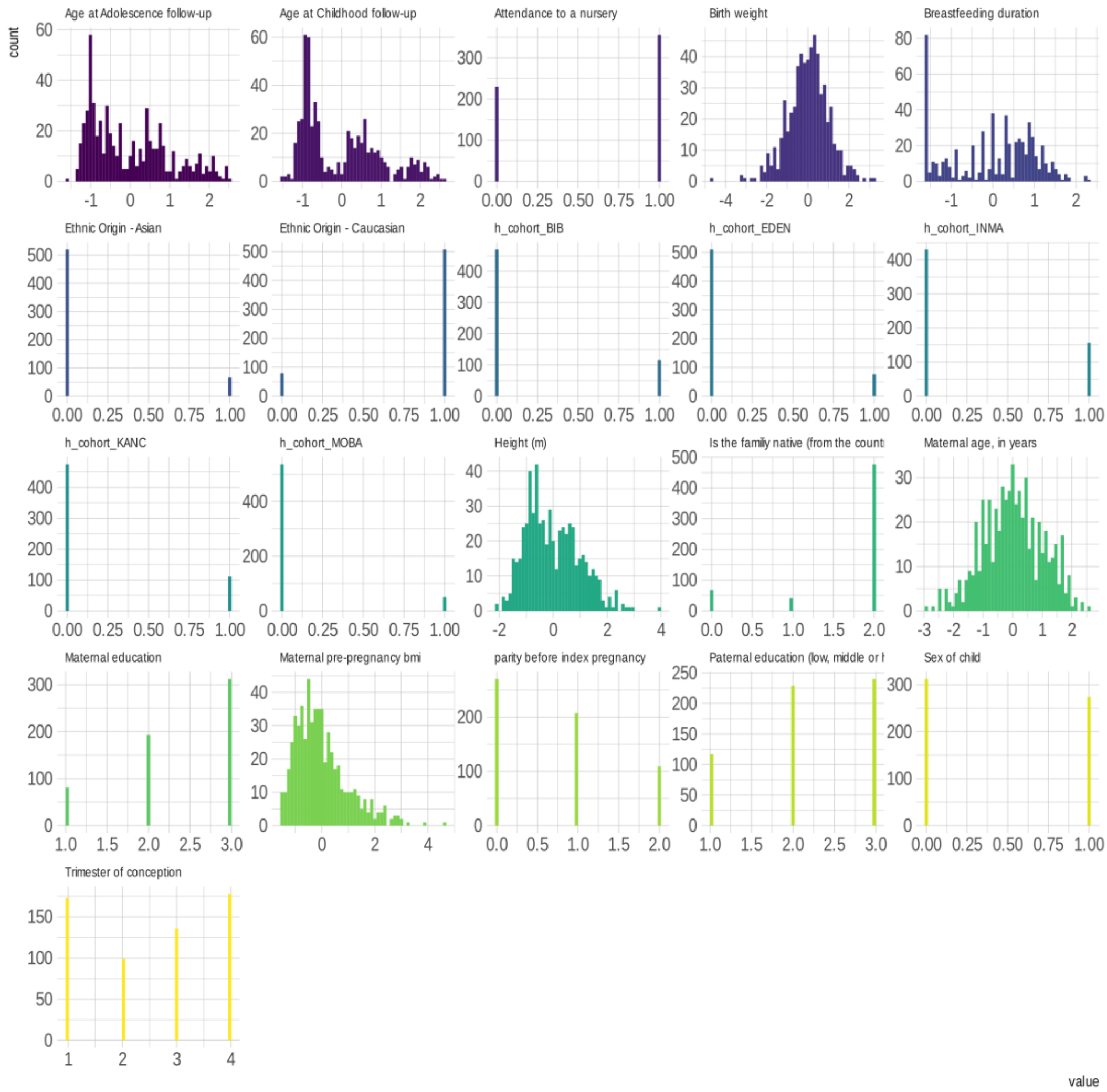

Supplementary Figure 4. Histograms of identified confounders. *Binary confounders (cohort, sex) were recoded to 0/1.*

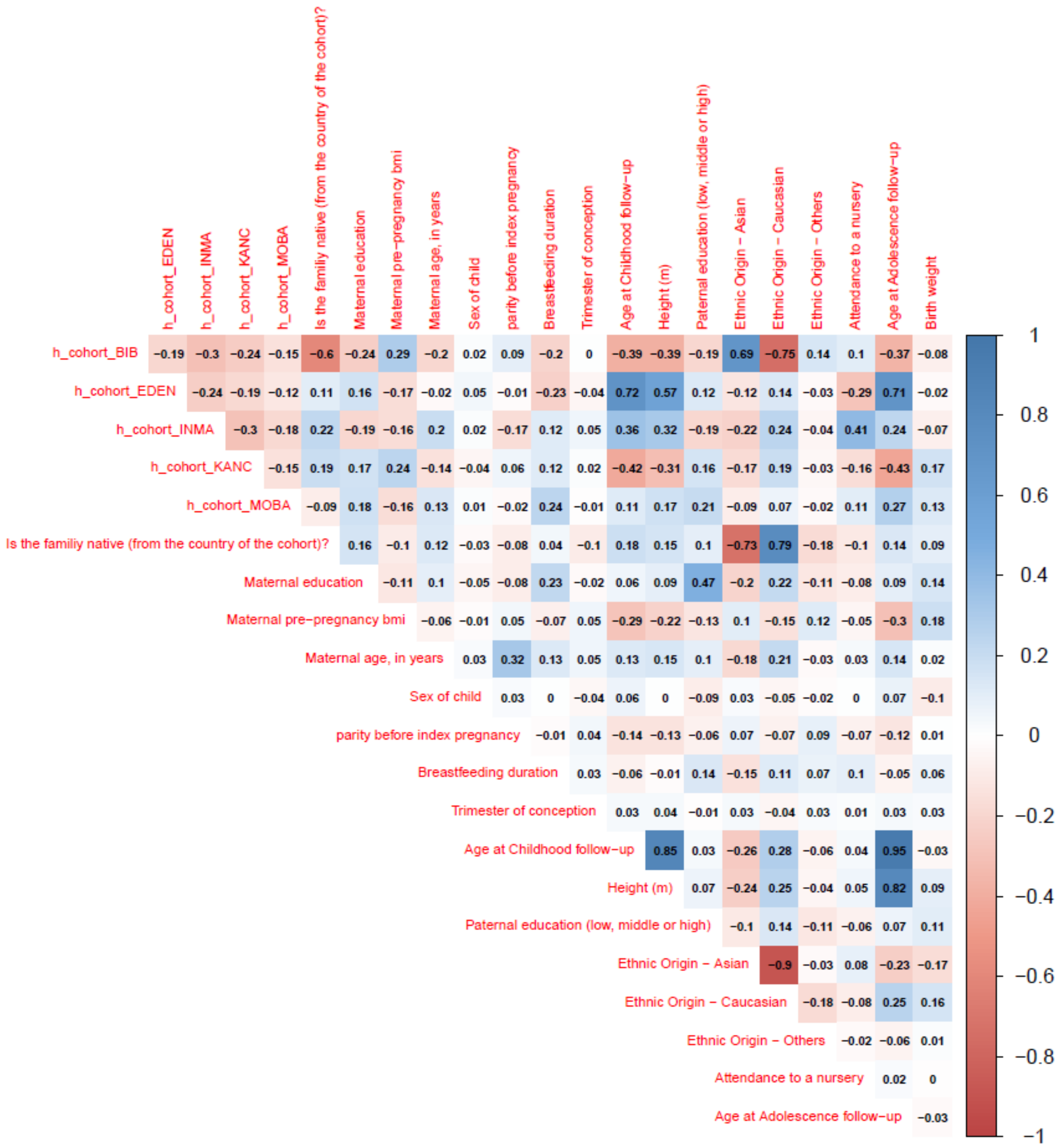

Supplementary Figure 5. Correlation heatmap for identified confounders.

4. Section IV. Statistical analyses

4.1. Data pre-processing

For each variable, the optimal transformation to approach normality was chosen using a Box-Cox power transformation approach. When normality could not be achieved by transformation, the variable was categorized. More details on the transformation used for each variable and their missing rate are provided in **Supplementary Table 1**. For biomarker exposures, values under the limit of detection were imputed using a distribution-based method (100). To allow the comparison between exposures and outcomes, standardized beta coefficients are presented where continuous exposures and outcomes have been centered and scaled previously to data modelling.

4.2. Imputation process

To prevent losing information and introducing potential selection biases, missing values of exposures and confounders were imputed. When possible, we relied on the multiple imputation procedure, which is a commonly used and accepted method to deal with missing data (101). Multiple imputation provides valid inferences under the missing at random (MAR) assumption, which assumes that missing data are associated with observed variables and not with unobserved information. In multiple imputation, missing values are imputed stochastically several times. Imputing missing values several times allows the quantification of the uncertainty in results associated with imputation, and to account for this uncertainty in the final standard errors, confidence intervals and p-values.

For the imputation process, continuous variables should have a normal distribution. As mentioned above, skewed exposure variables were transformed to achieve normality or categorized if no transformation worked. The distributions of all transformed variables were examined to make sure that transformations did not lead to extreme/influential observations. In cases of variables with zeros that required a log transformation, a constant value was added to the variable as the log of zero is minus infinity. The constant value was chosen to minimize the skewness of the resulting variable. Exposures with more than 70% of missing values were excluded from the process.

Missing values of exposures and adjustment variables were imputed using the method of chained equations (60), using the mice package in R (102). The large number of variables involved in the HELIX analyses implies that not all exposures can be used as predictors in imputation models, as imputation models would be too large, and therefore reduced imputation models had to be specified. It is recommended that imputation models include between 10 and 25 variables (102). We used the quickpred function to reduce the number of predictors. With this function, potential predictors for a given variable var1 were restricted to those that had: 1) an absolute correlation

greater than 0.2 with var1 or with the binary variable indicating if var1 is missing (mincor=0.2); and 2) a proportion of non-missing observations greater than 40% among the observations with missing values in var1 (minpuc=0.4). Values for mincor and minpucwere tuned to have a number of predictors between 5 and 25 for all variables. If some variables continued to have too many predictors, they were reduced manually. In addition, we forced into the imputation models cohort and one representative outcome variable of each the main groups of health outcomes that will be subsequently analyzed in the HELIX project (namely asthma as representative of respiratory health; z-score of BMI as representative of cardio metabolic outcomes; and number of correct responses in RAVEN test and total problems in CBCL as representative of neurodevelopment). Those variables were included with the include option. With the procedures described, the imputation process still produced some errors, which were solved on a case-by-case basis. Mostly, this consisted in removing from the predictor set of a given variable highly correlated variables. The method of predictive mean matching was used for all continuous exposures. Twenty imputed datasets were created.

After imputation, the following diagnostics were conducted. All variables with missing values were inspected. In particular, the imputed and non-missing observations were compared using density plots and stripplots. These types of comparisons were only done when there were more than 5% of the observations with missing values. Numerically, variables were flagged if they have 1) an absolute difference between means of the observed and imputed values greater than 2 standard deviations; or 2) a ratio of variances of the observed and imputed values that is less than 0.5 or greater than 2 (101). For categorical variables, those with a significant chi-squared test between imputed and non-imputed were examined. If variables were flagged and imputations seemed implausible, the predictors included in the imputation model for that variable were changed to that plausible imputations were generated.

4.3. Imputed exposome data exploration

A total of 25 families of exposures were successfully imputed in each mother-child pair, with 69 variables in the pregnancy stage and 129 in the early childhood. The correlation plots for both pregnancy and childhood exposures are available in **Supplementary Figures 5** and **6**.

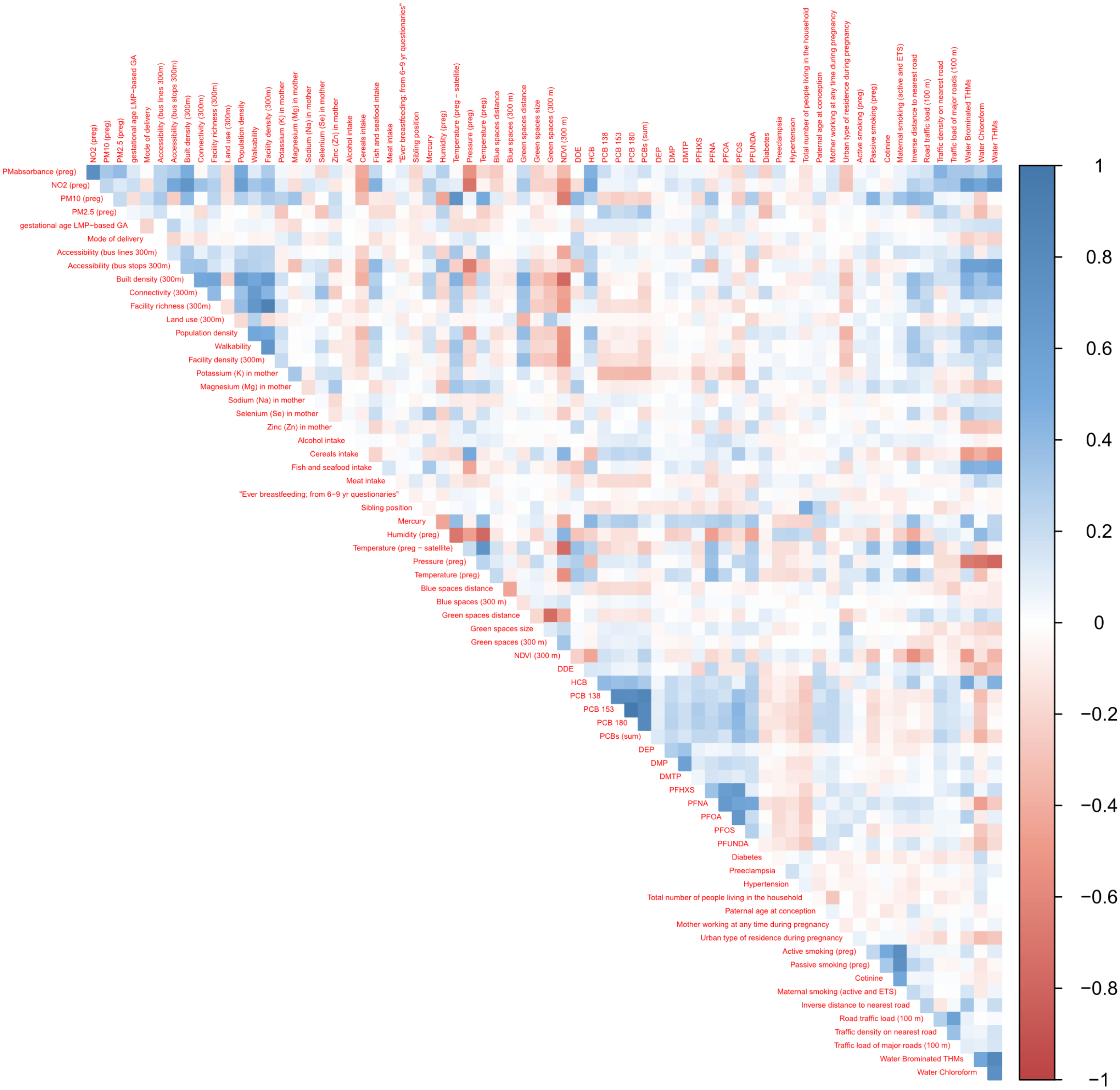

Supplementary Figure 6. Correlation heatmap for pregnancy exposome variables. *Sixty-nine exposures were initially included as part of the pregnancy exposome expanding 16 families (Air Pollution, Birth-related, Built Environment, Essential minerals, Lifestyle, Metals, Meteorological, Natural Spaces, OCs, OP Pesticides, PFASs, Pregnancy clinical, Socio-demographic, Tobacco Smoke, Traffic, and Water DBPs).*

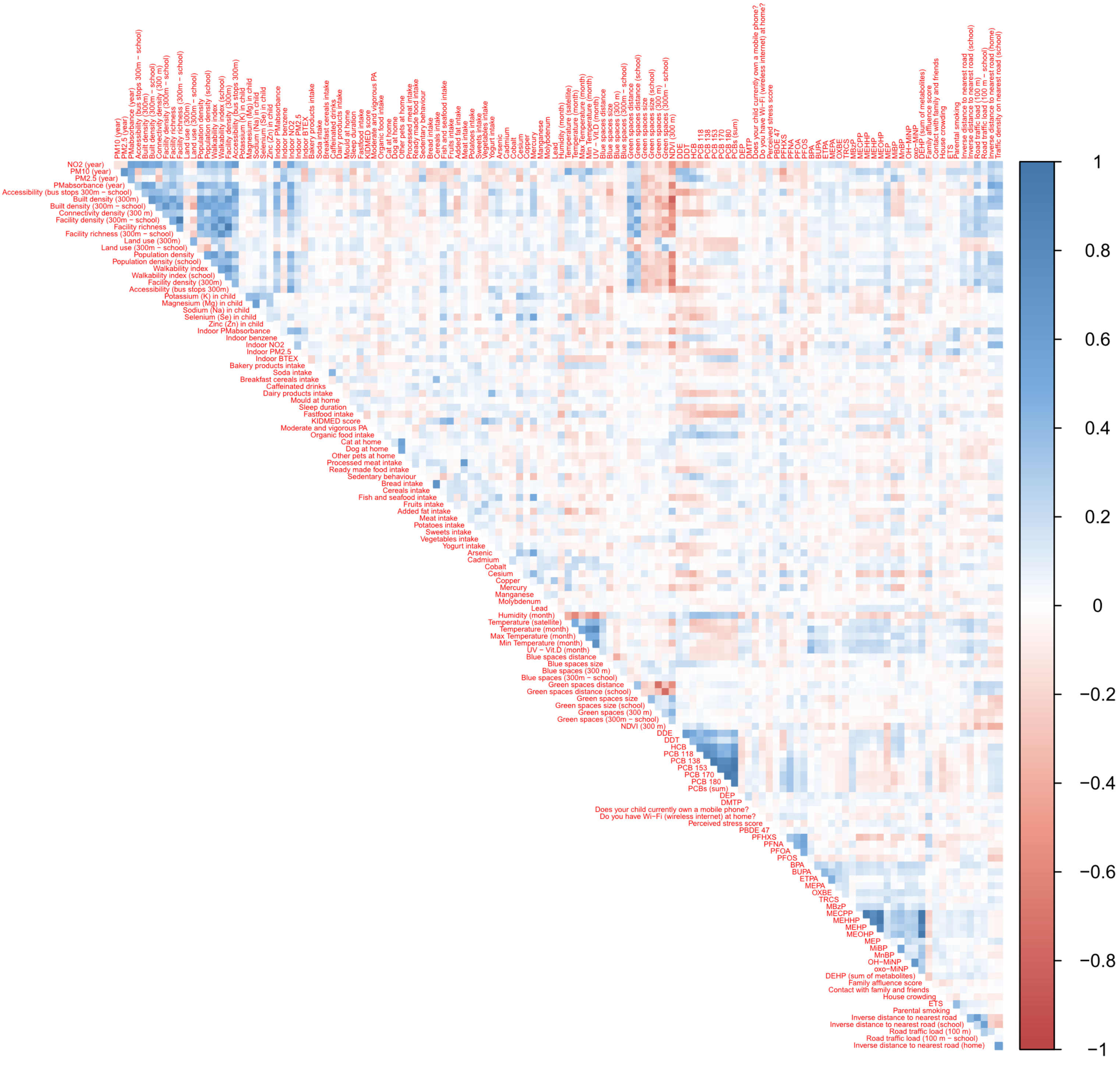

Supplementary Figure 7. Correlation heatmap for childhood exposome variables. *One-hundred thirty exposures were initially included as part of the childhood exposome expanding 17 families (Air Pollution, Built Environment, Essential minerals, Indoor air, Lifestyle, Metals, Meteorological, Natural Spaces, OCs, OP Pesticides, PBDEs, PFASs, Phenols, Phthalates, Socio-eco capital, Tobacco Smoke, and Traffic).*

4.4. Reduced-rank regression for outcome-wide analysis

The methodological goodness of the outcome-wide approach for the study of exposome-health associations has been recently reviewed (17), with specific conclusions on the most suitable methods and tools for its implementation. Among the different methods suitable for outcome-wide exposome analysis, the reduced rank regression (RRR) technique stands out as one of the most promising and useful algorithms, given its ability to handle the majority of hurdles encountered when working with multiple health domains in the context of the exposome (high dimensionality, multicollinearity, missing values, mixed-type outcomes, etc.).

RRR and its multiple variants are a group of dimensionality reduction techniques that assume that all the outcomes and exposures are associated through a shared low-dimensional subspace of latent factors (18, 103). The computation of such latent factors in RRR is guided by the maximization of a quantity representing both the variance in the outcome space and the relationship existing between predictors and outcomes [*Corr2(X,Y)*Var(Y)*, where *X* and *Y* are centered and scaled exposures (NxP) and outcomes (N×Q) matrices, respectively]. This way, RRR takes advantage of the existing correlations between outcome variables to improve predictive accuracy. In RRR, exposome-health associations for each outcome and exposure are individually quantified in the form of regression coefficients (beta estimates, β), so that output beta estimates are presented as a *PxQ* matrix where *P* refers to the number of assessed exposures and *Q* to the number of outcomes. In this matrix, each row represents the coefficient estimates for one predictor on all outcomes, and each column represents the coefficient estimates for all predictors on one outcome. RRR makes a restriction on the rank of the output regression coefficient matrix, which reduces the number of parameters to be estimated and improves the efficiency of estimation. Thanks to that, RRR is also efficient in dealing with multicollinearity among predictors and favors the selection of the outcome-associated exposures.

Here, we applied an extension of RRR called mixed-response reduced-rank regression (mRRR) model, given its ability to accommodate both continuous and binary outcomes. Moreover, mRRR permits dealing with incomplete records, allowing to skip the imputation of missing outcome values or the use of complete cases only. The mRRR was applied using the R package "rrpack". As a new functionality, we implemented a new feature-selection step into the algorithm and the new method was renamed as mixed-response sparse reduced-rank regression (msRRR). Specifically, denote the multivariate outcome matrix as $Y \in R^{n\times Q}$, the exposure matrix as $X \in R^{n\times P}$, the covariate matrix as $Z \in R^{n\times P_0}$, the msRRR aims to minimize the objective function

$$-logL(Y,X,Z) + \lambda\left||U|\right|_{2,1}, s.t. V^T V = I_r,$$

where $logL$ is the sum of the log-likelihood function of generalized linear models $y_k \sim XUv_k + Za_k$ with the missing outcome variables omitted. Matrices $B = UV^T \in R^{P\times Q}$ $and\ A =$

$\left[a_1, \dots a_Q\right]^T \in R^{P_0 \times Q}$ are the coefficient matrices for exposures and covariates respectively, with $U \in R^{P \times r}, V = \left[v_1, \dots, v_Q\right]^T \in R^{Q \times r}$, and $r < min(P, Q)$ is the rank to be determined. The penalty term involves the row-wise matrix norm $||U||_{2,1} = \sum_{i=1}^{P} \quad ||U_i,||_2$ and the tuning parameter λ. This penalty term is a group-lasso type penalty, which shrinks some entire rows of the coefficient's matrix towards zero. The reduced-rank coefficient matrix structure and the sparsity constraints in msRRR allowed for the identification of a small set of important exposure variables to associate with the inter-dependent outcome variables (as U is row-wise sparse) via a few latent factors (i.e. XU). The general strategy employed with msRRR for outcome-wide analysis is presented in **Figure 1** and **Supplementary Figure 11**. Two main approaches were conducted (cross-domains and domain-wise). In the cross-domains approach, all outcomes of interest were modelled together (regardless of the health area they belong to). In domain-wise approaches, a different msRRR model was applied to each group of outcomes (e.g., cardiometabolic, mental health, respiratory, and allergy). Cross-domains and domain-wise approaches offer complementary visions on the relationship between early life exposures and multiple health domains. While the cross-domain model allows the identification of environmental exposures affecting different health domains simultaneously, domain-wise models permit deepening into associations within each health family, highlighting exposures affecting multiple traits within the same domain. This gave a total of 10 separate msRRR models. The optimization of model hyperparameters (number of latent factors and penalty) was done through 5 folds-cross validation (CV). For each model, the whole population was partitioned into a training set including 80 % of participants for model training (hyperparameter optimization through 5-CV), and a test set with the remaining 20 % for model evaluation according to predictive performance metrics (**Supplementary Figure 12**). All population splits were created ensuring the equidistribution of cohorts and binary outcomes across partitions. Once the optimal hyperparameters were identified, a new model using the whole population sample was created using such hyperparameters and these models was used for interpretation.

Additionally, following recommendations from Anguita-Ruiz *et al*. (2023) (17), we implemented a post-selection inference approach for the curation of outcome-wide findings. The motivation for that is the fact that we may not always expect that evaluated exposures affect all outcomes under study (as msRRR assumes). To achieve such intra-row sparsity in the output coefficient matrix, we adopted the concept of stability selection (104). In stability selection, a feature selection model is combined with resampling procedures to compute bootstrap p-value for evaluating the stability and robustness of each exposure-outcome association estimated by msRRR. Stability selection results are less likely to be driven by outlying observations and the significance can be interpreted as a measure of feature importance, conditionally on all other important features. The procedure of estimation of bootstrap p-values for each exposure-outcome

association was implemented according to Garali et al. (108). Briefly, each final model was fitted 500 times in population bootstrap subsets with replacement to derive a p-value for each exposure-outcome association, allowing to evaluate the stability and robustness of each association.

4.5. Statistical analysis

Before outcome-wide analysis, the dimensionality of the exposome dataset was reduced following recommendations and lessons learned from previous simulation studies (12, 63). The high dimensionality of the exposome is a well-known complication in the case of low-to-moderate sample sizes, and it is exacerbated when multiple outcomes are considered as it requires the exploration of how exposome domains could be affecting each of the different studied phenotypes. This requires study populations with a large enough sample size (i.e., "one in ten rule" for each outcome) to robustly test multiple research hypotheses as well as to incorporate effective strategies for feature selection during the model training. Another issue related to the high dimensionality problem in outcome-wide analysis is the increased probability of making one or more false discoveries when testing multiple hypotheses (family-wise error rate), which should also be addressed through the incorporation of adequate inference techniques.

Exposome-wide association study (ExWAS) analysis has been proposed as a first-stage method for feature selection, to filter down the number of exposures passing to downstream advanced analyses, given its ability to select markers while keeping high sensitivity rates (i.e., a low number of false negatives) in previous simulations (12, 63). The 20 imputed datasets were used and Rubin's rules were employed to combine the results. Using ExWAS, exposure-by-exposure estimations of the association with each of the health outcomes of interest were conducted, using multivariate linear or logistic regressions adjusted for confounders. To account for multiple comparisons, a family-wise error rate correction was used to correct the p-value threshold (5% divided by the effective number of tests per outcome). Individual estimates for each exposure-outcome pair can be found in **Online Supplementary catalogue (https://outcomewide.isglobal.org/).** Exposures showing association **(Nominal *P-value* < 0.1)** with at least two childhood outcomes or two adolescent outcomes were considered **(Supplementary Figure 8)**. Among those, overlapping exposures between childhood and adolescence approaches were finally retained (excluding those exposures showing correlations $r^2>0.75$ **(Supplementary Figures 8** and **9)).** After ExWAS, the final dimension of exposure datasets for outcome-wide analysis was P=17 for pregnancy exposures and P=56 for childhood exposures (**Supplementary Tables 12** and **13**).

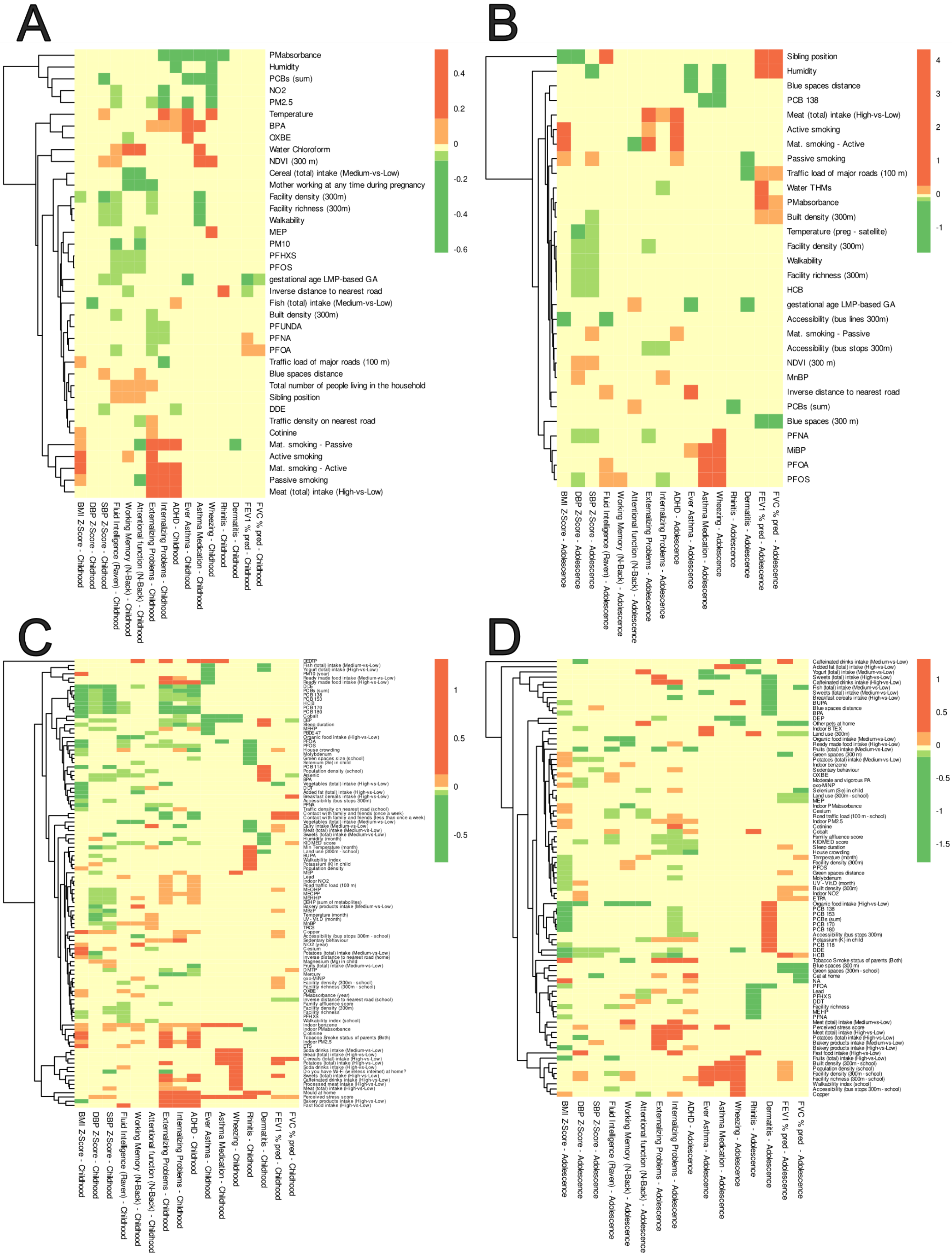

A
PMabsorbance
Humidity
PCBs (sum)
NO2
PM2.5
Temperature
BPA
OXBE
Water Chloroform
NDVI (300 m)
Cereal (total) intake (Medium-vs-Low)
Mother working at any time during pregnancy
Facility density (300m)
Facility richness (300m)
Walkability
MEP
PM10
PFHXS
PFOS
gestational age LMP-based GA
Inverse distance to nearest road
Fish (total) intake (Medium-vs-Low)
Built density (300m)
PFUNDA
PFNA
PFOA
Traffic load of major roads (100 m)
Blue spaces distance
Total number of people living in the household
Sibling position
DDE
Traffic density on nearest road
Cotinine
Mat. smoking - Passive
Active smoking
Mat. smoking - Active
Passive smoking
Meat (total) intake (High-vs-Low)
0.4
0.2
0
-0.2
-0.4
-0.6
BMI Z-Score - Childhood
DBP Z-Score - Childhood
SBP Z-Score - Childhood
Fluid Intelligence (Raven) - Childhood
Working Memory (N-Back) - Childhood
Attentional function (N-Back) - Childhood
Externalizing Problems - Childhood
Internalizing Problems - Childhood
ADHD - Childhood
Ever Asthma - Childhood
Asthma Medication - Childhood
Wheezing - Childhood
Rhinitis - Childhood
Dermatitis - Childhood
FEV1 % pred - Childhood
FVC % pred - Childhood
B
Sibling position
Humidity
Blue spaces distance
PCB 138
Meat (total) intake (High-vs-Low)
Active smoking
Mat. smoking - Active
Passive smoking
Traffic load of major roads (100 m)
Water THMs
PMabsorbance
Built density (300m)
Temperature (preg - satellite)
Facility density (300m)
Walkability
Facility richness (300m)
HCB
gestational age LMP-based GA
Accessibility (bus lines 300m)
Mat. smoking - Passive
Accessibility (bus stops 300m)
NDVI (300 m)
MnBP
Inverse distance to nearest road
PCBs (sum)
Blue spaces (300 m)
PFNA
MiBP
PFOA
PFOS
4
3
2
1
0
-1
BMI Z-Score - Adolescence
DBP Z-Score - Adolescence
SBP Z-Score - Adolescence
Fluid Intelligence (Raven) - Adolescence
Working Memory (N-Back) - Adolescence
Attentional function (N-Back) - Adolescence
Externalizing Problems - Adolescence
Internalizing Problems - Adolescence
ADHD - Adolescence
Ever Asthma - Adolescence
Asthma Medication - Adolescence
Wheezing - Adolescence
Rhinitis - Adolescence
Dermatitis - Adolescence
FEV1 % pred - Adolescence
FVC % pred - Adolescence
C
1
0.5
0
-0.5
BMI Z-Score - Childhood
DBP Z-Score - Childhood
SBP Z-Score - Childhood
Fluid Intelligence (Raven) - Childhood
Working Memory (N-Back) - Childhood
Attentional function (N-Back) - Childhood
Externalizing Problems - Childhood
Internalizing Problems - Childhood
ADHD - Childhood
Ever Asthma - Childhood
Asthma Medication - Childhood
Wheezing - Childhood
Rhinitis - Childhood
Dermatitis - Childhood
FEV1 % pred - Childhood
FVC % pred - Childhood
D
1
0.5
0
-0.5
-1
-1.5
BMI Z-Score - Adolescence
DBP Z-Score - Adolescence
SBP Z-Score - Adolescence
Fluid Intelligence (Raven) - Adolescence
Working Memory (N-Back) - Adolescence
Attentional function (N-Back) - Adolescence
Externalizing Problems - Adolescence
Internalizing Problems - Adolescence
ADHD - Adolescence
Ever Asthma - Adolescence
Asthma Medication - Adolescence
Wheezing - Adolescence
Rhinitis - Adolescence
Dermatitis - Adolescence
FEV1 % pred - Adolescence
FVC % pred - Adolescence

Supplementary Figure 8. Estimated coefficients for each exposure-outcome pair association from ExWAS models. *Panels on the left show childhood stage outcomes, while panels on the right show the results for adolescent follow-up outcomes. Panels (**A-B**) show associations from pregnancy exposures, while panels (**C-D**) show associations from early-childhood exposures. Beta estimates for outcome-exposure pairs with a nominal P-value > 0.1 were set to zero (non-significant).*

Supplementary Table 12. Numbers of pregnancy exposures selected by ExWAS.

Type of exposure	Exposures	N of exposures	N of exposures selected by ExWAS
	**URBAN EXPOSOME**		
**Outdoor air pollution ***	NO_2, $PM_{10.}$ $PM_{2.5}$, $PM_{2.5}$ absorbance	4	1
**Meteorology ***	Temperature, humidity, pressure	4	1
**Surrounding natural spaces ***	NDVI, presence/distance/size of a major greenspaces and blue space	6	2
**Built environment ***	Population density, building density 300m, Connectivity 300m, Bus public transport lines 300m, Bus public transport stops 300m, Facility density 300m, Facility richness 300m, Land use 300m, Walkability	9	3
**Road traffic and noise ***	Traffic load on all roads and major road (100m), traffic density on nearest road, inverse distance to the nearest road	4	2
**Water DBPs**	THMs, brominated THMs, chloroform	3	0
Total of urban exposures		30	9
	**LIFESTYLE & OTHER BIRTH-RELATED FACTORS**		
**Tobacco/Alcohol ***	Alcohol intake, Active and passive smoking	4	1
**Diet ***	Cereal, Meat, and Fish intake	3	1
**Breastfeeding**	Ever breastfeeding	1	0
**Birth-related ***	Mode of delivery, gestational age	2	1
**Socio-economic ***	Sibling position, Type of residence, working mother, paternal age, house crowding	5	1
**Pregnancy clinical**	Diabetes, preeclampsia, hypertension	3	0
Total of lifestyle & other birth-related exposures		18	4
	**CHEMICAL EXPOSOME**		
**Organochlorine pesticides (OCs) ***	PCB138, PCB153, PCB180, PCBsum[a], DDE, HCB	6	1
**Perfluoroalkyl substances (PFASs) ***	PFHxS, PFOS, PFOA, PFNA, PFUnDA	5	3
**Metal and essential elements**	Hg, K, Mg, Na, Se, Zn	6	0
**Organophosphate (OP) metabolites**	DMP, DMTP, DEP	3	0
**Tobacco**	Cotinine	1	0

Total of chemical exposures	21	4
**Total number of exposures**	**69**	**17**

[a] *PCB138 + PCB153 + PCB 180.*
* *Refers to domains that were retained for the outcome-wide analysis.*

Supplementary Table 13. Numbers of childhood exposures selected by ExWAS.

Type of exposure	Exposures	N of exposures	N of exposures selected by ExWAS
	**URBAN EXPOSOME**		
**Outdoor air pollution**	NO_2, $PM_{10.}$ $PM_{2.5}$, $PM_{2.5}$ absorbance	4	0
**Indoor air pollution ***	NO_2, $PM_{2.5}$, PM absorbance, Benzene, TEX	5	4
**Meteorology/UV ***	Temperature, humidity, UV-vit D	6	1
**Surrounding natural spaces**	NDVI (300m), presence/distance/size of a major greenspaces and blue spaces at home and school (300m buffer)	11	0
**Built environment ***	Accessibility, population density, building density, street connectivity, accessibility, facility richness, walkability, land use index (300m buffer)	15	8
**Road traffic and noise**	Traffic load on all roads and nearest road (100 m buffer), traffic density on nearest road, inverse distance to nearest road. Traffic noise at home and school during the day	6	0
Total of urban exposures		47	13
	**LIFESTYLE**		
**Tobacco ***	Child exposure to smoke (ETS) and parental smoking	2	1
**Diet ***	Foods groups intakes, adherence to the Mediterranean diet (KIDMED score)	20	15
**Sleep**	Average sleep	1	0
**Physical activity ***	Moderate/vigorous activity, sedentary time	2	1
**Allergens**	Mould at home, Dogs, Cats or other Pet at home	4	0
**Socio-economic ***	Family Affluence Score (FAS), family contact, house crowding, WIFI at home, smartphone	5	2
**Stress ***	Mother's perceived child's stress	1	1
Total of lifestyle exposures		35	20
	**CHEMICAL EXPOSOME**		
**Organochlorine pesticides (OCs) ***	PCB118, PCB 138, PCB153, PCB170, PCB180, PCBsum[a], DDE, DDT, HCB	9	5
**Perfluoroalkyl substances (PFASs) ***	PFHxS, PFOS, PFOA, PFNA	4	4
**Brominated compounds (PBDEs)**	PBDE 47	1	0
**Metal and essential elements ***	As, Cd, Co, Cu, Cs, Hg, K, Mg, Mn, Mo, Na, Pb, Se, Zn	14	7

**Phthalates ***	MECPP, MEHHP, MEHP, MEOHP, MEP , MiBP, MnBP, MBzP, DEHP [b], oxo-MiNP and oh-MiNP	11	3
**Phenols ***	MEPA, ETPA, BUPA, BPA, OXBE, TRCS	6	3
**Organophosphate (OP) metabolites ***	DMTP, DEP	2	1
Total of chemical exposures		47	23
**Total number of exposures**		**129**	**56**

[a] *PCB118 + PCB 138 + PCB153 + PCB170 + PCB180.*

[b] *DEHP: molar sum of MEHP, MEHHP, MEOHP and MECPP.*

** Refers to domains that were retained for the outcome-wide analysis.*

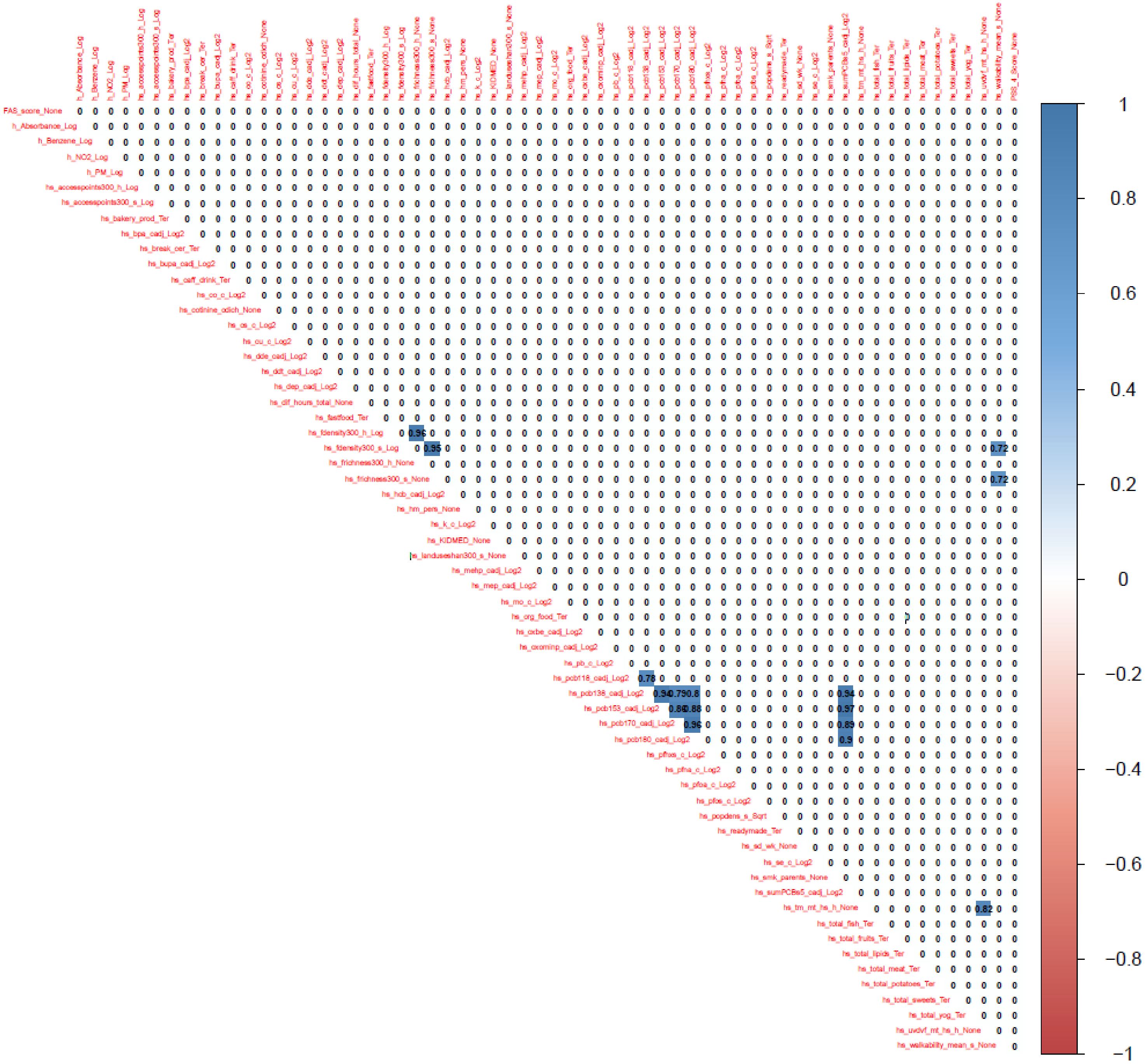

Supplementary Figure 9. Correlation plot for selected childhood exposures after ExWAS filtering step.

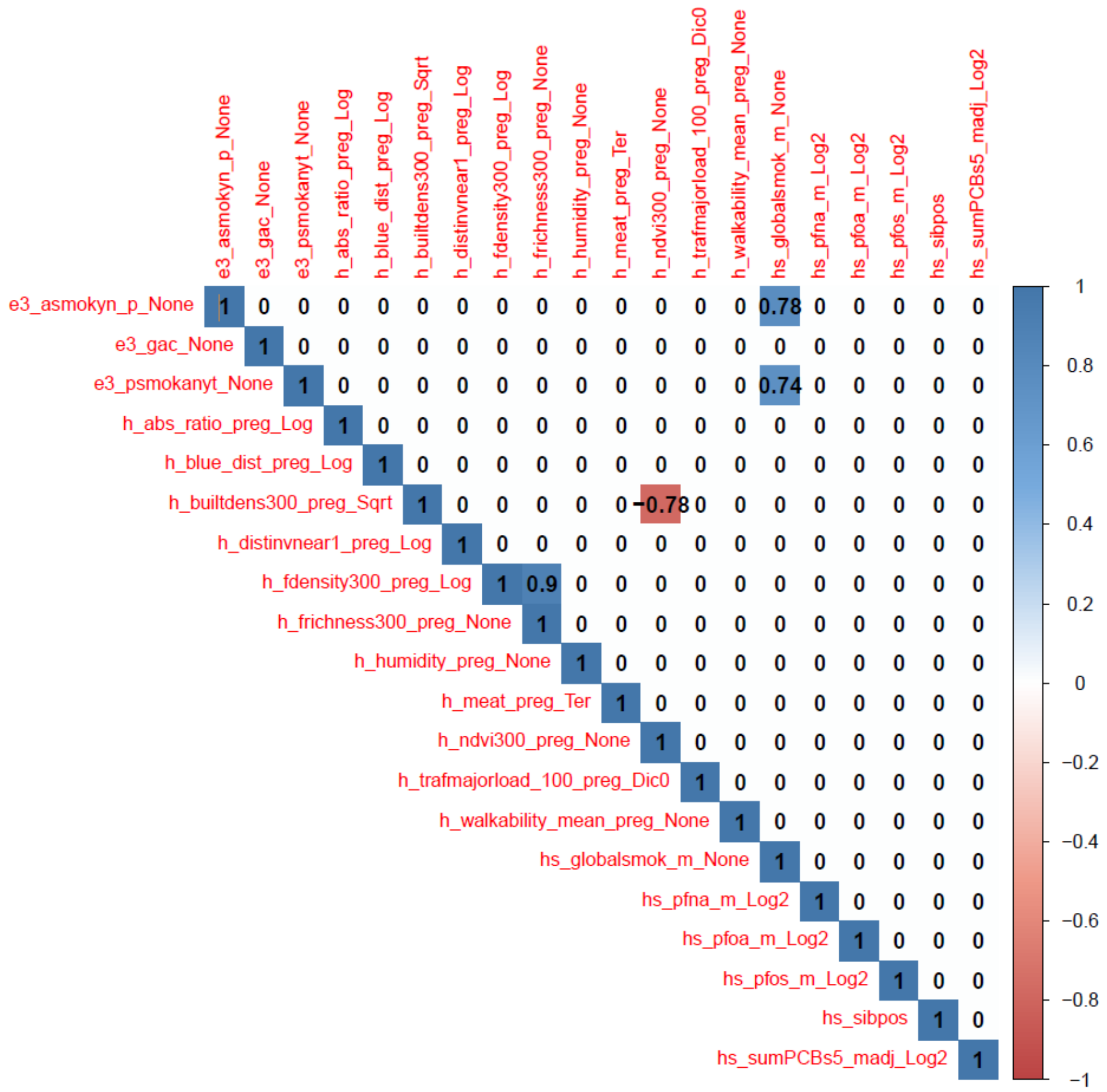

Supplementary Figure 10. Correlation plot for selected pregnancy exposures after ExWAS filtering step.

Motivated by the high correlation existing for exposures between pregnancy and childhood stages, msRRR was applied separately to pregnancy and childhood exposures. Outcomes from childhood and adolescence stages were instead modeled together given the ability of msRRR to exploit correlations in the outcome space. The msRRR was applied following two distinct approaches (**Supplementary Figure 11**). In the cross-domains approach, all outcomes of interest were modeled together (regardless of the health area they belong to). In the domain-wise approaches, a different msRRR model was applied to each group of outcomes (e.g., cardiometabolic, mental health, respiratory, and allergy). Cross-domains and domain-wise approaches offer complementary visions of the relationship between early life exposures and multiple health domains. While the cross-domain model allows the identification of environmental exposures affecting different health domains simultaneously, domain-wise models permit deepening into associations within each health family, highlighting exposures affecting

multiple traits within the same domain. This gave a total of 10 msRRR independent models (**Supplementary Figure 11**).

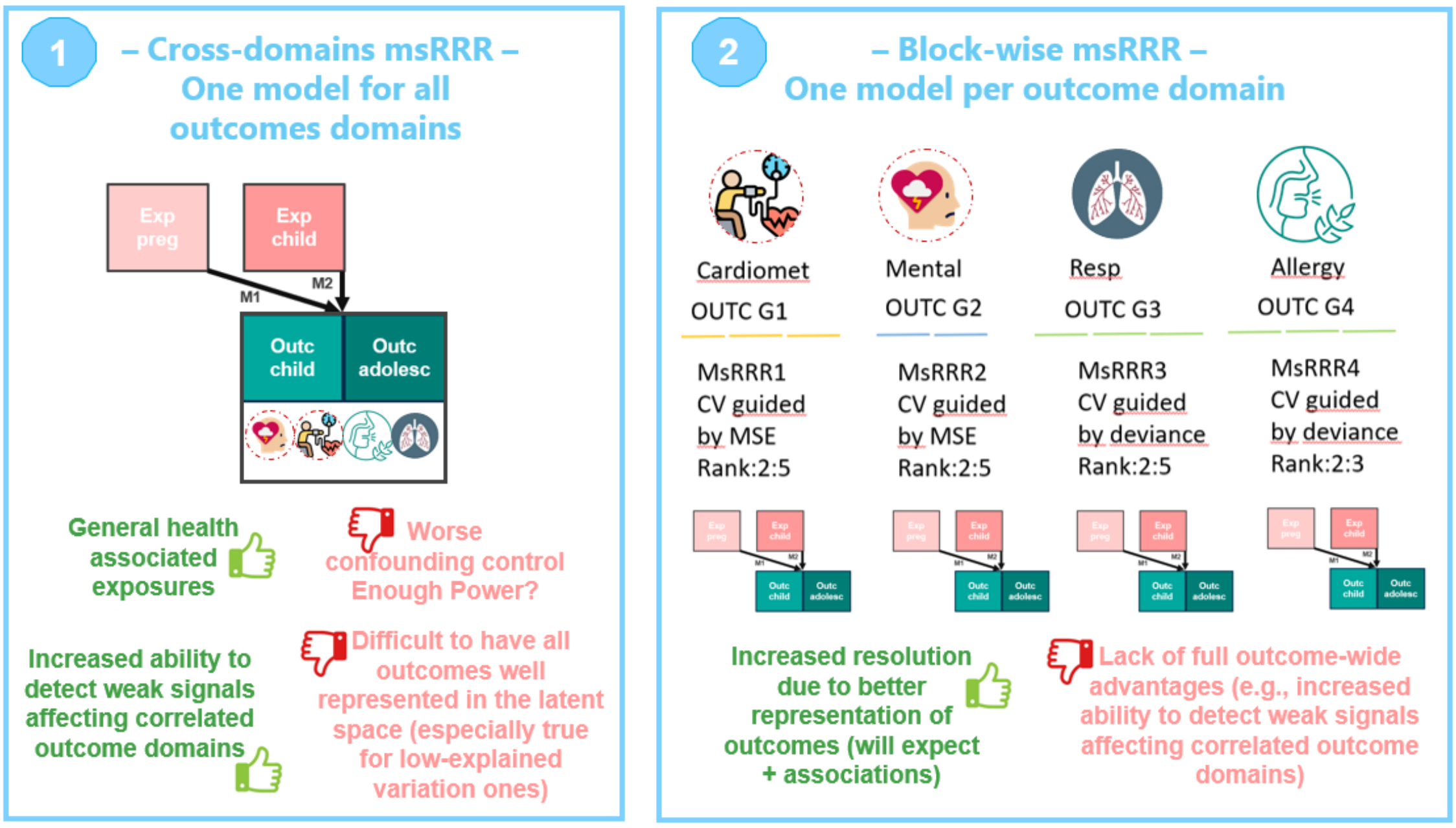

Supplementary Figure 11. Adopted approaches for outcome-wide analysis in our study.

Two hyperparameters had to be defined for each msRRR model; the rank or number of latent factors, and the L1 penalty for sparsity. Hyperparameters optimization for each msRRR model was done through 5 folds-cross validation (CV), guiding the evaluation of model performance on the deviance criterion if binary outcomes were considered (which is a comparable metric for both continuous and binary outcomes), or on the mean squared error if only continuous outcomes were considered. The range of input values for the identification of optimal hyperparameters in each model is defined in **Supplementary Table 14**. The definition of each range of latent factors was done after exploration of the correlation structure in the outcome space, which helped to identify the expected number of latent variables summarizing each group of outcomes (**Figure 2**). The optimal L1 penalty was identified among 100 different input values (**Supplementary Table 14**).

The adopted population stratification strategy for model performance evaluation is represented in **Supplementary Figure 12**. The population was split into a training set including 80 % of participants for model training (hyperparameter optimization through 5-CV) and a test set with the remaining 20 % for model evaluation. All population splits were created ensuring the equidistribution of cohorts and binary outcomes across partitions. For model interpretation, a final model was built in the whole population with identified optimal hyperparameters. The final

model was built 500 times in bootstrap subsets of the whole population with resampling to compute bootstrap confidence intervals and p-values for evaluating the significance/stability of each exposure-outcome association.

Supplementary Table 14. Range of input values for hyperparameters optimization in each msRRR model.

Model	N of outcomes modeled	Rank/Latent factors	Lambda (L1) penalty	Criterion guiding the search for optimal hyperparameter
Cross-domains msRRR (pregnancy)	24	3,4,5,6,7,8	10^(seq(log10(1e-04), log10(1), len = 100))	Deviance
Cross-domains msRRR (childhood)	24	3,4,5,6,7,8	10^(seq(log10(1e-04), log10(1), len = 100))	Deviance
Cardiometabolic Domain-wise msRRR (pregnancy)	6	2,3,4,5	10^(seq(log10(1e-04), log10(2), len = 100))	Mean squared error
Cardiometabolic Domain-wise msRRR (childhood)	6	2,3,4,5	10^(seq(log10(1e-04), log10(2), len = 100))	Mean squared error
Mental Domain-wise msRRR (pregnancy)	6	2,3,4,5	10^(seq(log10(1e-04), log10(2), len = 100))	Mean squared error
Mental Domain-wise msRRR (childhood)	6	2,3,4,5	10^(seq(log10(1e-04), log10(2), len = 100))	Mean squared error
Respiratory Domain-wise msRRR (pregnancy)	6	2,3,4,5	10^(seq(log10(1e-04), log10(2), len = 100))	Deviance
Respiratory Domain-wise msRRR (childhood)	6	2,3,4,5	10^(seq(log10(1e-04), log10(2), len = 100))	Deviance
Allergy Domain-wise msRRR (pregnancy)	4	2,3	10^(seq(log10(1e-04), log10(2), len = 100))	Deviance
Allergy Domain-wise msRRR (childhood)	4	2,3	10^(seq(log10(1e-04), log10(2), len = 100))	Deviance

The sequence of input lambda values is defined in R syntax due to the limit of space.

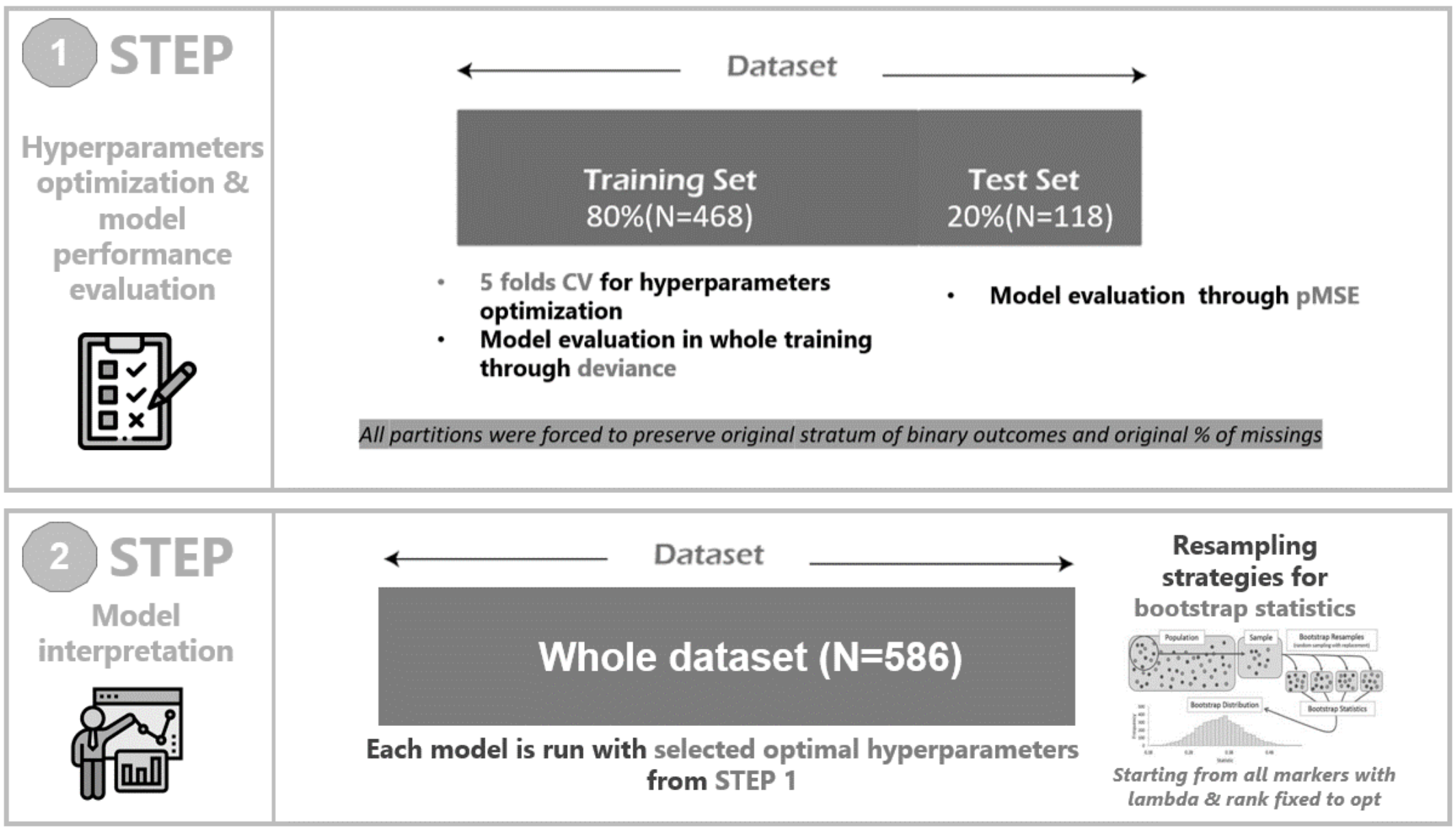

Supplementary Figure 12. Sample stratification strategy for model performance evaluation and interpretation. Abbreviations: CV, cross-validation; pMSE, pseudo-mean squared error.

While our initial msRRR framework mitigates multiple testing and multicollinearity (i.e., msRRR models the whole set of exposures all together under a dimensionality reduction framework designed to deal with correlated variables), the risk of cohort-driven false discoveries remained a significant concern in our study. To this end, our model was designed with the functionality to incorporate fixed confounders during the parameters' estimation. Still, we acknowledge that the adopted strategy of adjusting by cohort dummy variables not fully eliminated the concern, since it might not fully capture all sources of between-cohort heterogeneity.

For this reason, we decided to fully address it by implementing the Leave-One-Cohort-Out (LOCO) cross-validation sensitivity analysis. This represented an important computational and analytical effort aimed at demonstrating the robustness of our results. Specifically, we systematically re-ran all outcome-wide msRRR models (cross-domain and domain-wise, for both pregnancy and childhood exposure windows) six consecutive times, excluding a different cohort in each iteration. This resulted in 60 newly trained models with feature selection for outcome-wide exposure relevance, and which were subsequently subjected to bootstrap resampling each, to derive stability empirical p-values, adding intra-exposure sparsity. To establish a rigorous threshold for robustness when selecting exposure-outcome significant association pairs, we evaluated not only the frequency of each exposure-outcome pair selection across the 6 LOCO folds, but also the consistency of the effect direction and its cross-regional generalizability. To inform new results, we adopted a two stage-validation criterion:

- Frequency of selection and directional consistency: An exposure-outcome association had to be selected in the majority of the iterations (>4 out of 6 LOCO folds) maintaining the exact same effect sign direction (risk or protective). This threshold reflects a majority-based consensus criterion, ensuring that associations are reproducibly identified across different cohort configurations. We note that our conclusions were robust to nearby thresholds (e.g., ≥3 or ≥5 folds), and we therefore adopted ≥4 folds as a balanced and interpretable compromise between stringency and statistical power.

- Cross-regional robustness: Following our previous methodology in the same European multi-cohort setting (26), we stratified the six HELIX cohorts into two distinct geographic and socio-cultural blocks: South-Mediterranean (INMA, Spain; RHEA, Greece) and North-Western (MOBA, Norway; KANC, Lithuania; BIB, UK; EDEN, France). This macro-regional partition captures two distinct European realities characterized by divergent socioeconomic structures, urban configurations, and lifestyle habits (e.g., diet), which collectively drive the distribution of most early-life exposures far beyond mere individual, country-level variations. In order an exposure-outcome pair association to receive the designation of cross-regional robustness, we required it to remain selected in all LOCO iterations when excluding cohorts from both regions. For instance, if an association was selected in 4 folds but disappeared entirely whenever a South-Mediterranean cohort was excluded (i.e., 0/2 in South iterations), it was flagged as a region-driven phenomenon and failed this criterion. Conversely, if an association successfully survived the sequential exclusion of cohorts from both macro-regions, it earned the cross-regional robustness designation (denoted with an asterisk * in our new supplementary heatmaps). This second layer of stringency provides a powerful demonstration of generalizability, confirming that our reported "master regulators" represent European landscape-wide exposure associations rather than localized artifacts driven by specific regional or cultural phenomena.

5. Section V. Supplementary results:

In the present section, supplementary tables and figures that support the main findings are presented. These figures and tables are cited along the result section of the main paper.

Supplementary Table 15. General descriptive for the study population from childhood to adolescence stratified by country of recruitment.

Characteristics	RHEA			BIB			EDEN			INMA			KANC			MOBA			Two-way ANOVA on deltas (T1-T0)
	Early-childhood	Adolescence	p-value [1]	Early-childhood	Adolescence	p-value [1]	Early-childhood	Adolescence	p-value [1]	Early-childhood	Adolescence	p-value [1]	Early-childhood	Adolescence	p-value [1]	Early-childhood	Adolescence	p-value [1]	Overall P-Value[2]
	N=77	N=77		N=116	N=116		N=76	N=76		N=156	N=156		N=111	N=111		N=50	N=50		
Child's Sex																			
Male	46 (60%)			59 (51%)			36 (47%)			82 (53%)			63 (57%)			26 (52%)			
Female	31 (40%)			57 (49%)			40 (53%)			74 (47%)			48 (43%)			24 (48%)			
Child's Age	6.45 (0.25)	13.83 (0.33)	<0.001	6.61 (0.24)	13.92 (0.52)	<0.001	10.83 (0.57)	17.72 (0.58)	<0.001	8.77 (0.55)	15.58 (0.55)	<0.001	6.48 (0.53)	13.70 (0.43)	<0.001	8.49 (0.50)	16.39 (0.59)	<0.001	**<0.001**
Child's Height (m)	1.19 (0.04)	1.62 (0.07)	<0.001	1.20 (0.05)	1.62 (0.07)	<0.001	1.43 (0.08)	1.70 (0.10)	<0.001	1.33 (0.06)	1.68 (0.08)	<0.001	1.21 (0.06)	1.65 (0.09)	<0.001	1.34 (0.06)	1.74 (0.09)	<0.001	**<0.001**
Is the family native from the country of recruitment?																			.
Any	0 (0%)			54 (47%)			0 (0%)			0 (0%)			2 (1.8%)			12 (24%)			
One parent	3 (3.9%)			20 (17%)			9 (12%)			9 (5.8%)			0 (0%)			0 (0%)			
Both parents	74 (96%)			42 (36%)			67 (88%)			147 (94%)			109 (98%)			38 (76%)			
Maternal education																			
Low	2 (2.6%)			43 (37%)			3 (3.9%)			31 (20%)			2 (1.8%)			0 (0%)			
Middle	38 (49%)			28 (24%)			17 (22%)			67 (43%)			34 (31%)			9 (18%)			
High	37 (48%)			45 (39%)			56 (74%)			58 (37%)			75 (68%)			41 (82%)			
Maternal pre-pregnancy bmi	23.6 (3.6)			28.1 (5.4)			23.0 (4.2)			23.8 (4.2)			27.6 (4.8)			22.46 (3.01)			.
Maternal age, in years	31.5 (4.5)			28.8 (5.4)			30.5 (4.5)			32.4 (3.8)			29.4 (4.9)			32.9 (4.0)			.

Parity																			
Nulliparous	28 (36%)			48 (41%)			36 (47%)			84 (54%)			51 (46%)			23 (46%)			
Monoparous	32 (42%)			33 (28%)			27 (36%)			63 (40%)			31 (28%)			21 (42%)			
Multiparous	17 (22%)			35 (30%)			13 (17%)			9 (5.8%)			29 (26%)			6 (12%)			
Breastfeeding duration	4.89 (0.95)			4.52 (1.23)			4.27 (0.94)			5.22 (1.10)			5.24 (1.09)			5.89 (0.60)			.
Trimester of conception:																			
1	18 (23%)			44 (38%)			26 (34%)			37 (24%)			31 (28%)			17 (34%)			
2	23 (30%)			8 (6.9%)			18 (24%)			28 (18%)			15 (14%)			7 (14%)			
3	22 (29%)			19 (16%)			9 (12%)			42 (27%)			35 (32%)			9 (18%)			
4	14 (18%)			45 (39%)			23 (30%)			49 (31%)			30 (27%)			17 (34%)			
Parental Education																			
Low	7 (9.1%)			52 (45%)			7 (9.2%)			49 (31%)			2 (1.8%)			0 (0%)			
Middle	46 (60%)			21 (18%)			30 (39%)			63 (40%)			56 (50%)			13 (26%)			
High	24 (31%)			43 (37%)			39 (51%)			44 (28%)			53 (48%)			37 (74%)			
Ethnic Origin - Asian	0 (0%)			64 (55%)			1 (1.3%)			0 (0%)			0 (0%)			1 (2.0%)			
Ethnic Origin - Caucasian	77 (100%)			41 (35%)			75 (99%)			156 (100%)			111 (100%)			47 (94%)			
Ethnic Origin - Others	0 (0%)			11 (9.5%)			0 (0%)			0 (0%)			0 (0%)			2 (4.0%)			
Attendance to a nursery	18 (23%)			82 (71%)			16 (21%)			143 (92%)			59 (53%)			38 (76%)			
Birth weight (g)	3,251 (396)			3,289 (538)			3,305 (479)			3,274 (395)			3,502 (494)			3,542 (421)			.
Externalizing Problems - Scale	8.5 (7.2)	6.5 (4.9)	0.004	6 (7)	6 (7)	0.4	6.7 (5.7)	4.1 (4.9)	<0.001	6.4 (6.5)	5.8 (7.0)	0.2	7.8 (6.2)	7.1 (6.6)	0.2	2.18 (2.81)	2.66 (3.65)	0.6	**0.003**
Missing	1	0		0	1											0	6		

Internalizing Problems - Scale	5.9 (4.1)	7.9 (6.8)	0.008	5 (6)	9 (9)	<0.001	8.9 (5.8)	6.5 (5.7)	<0.001	7 (6)	8 (6)	0.6	7.7 (6.2)	8.7 (6.3)	0.2	3.22 (2.25)	4.84 (6.56)	0.073	**<0.001**
Missing	1	0		0	1											0	6		
ADHD Index	3.45 (2.71)	2.34 (2.38)	<0.001	3.16 (2.99)	3.22 (3.40)	0.7	2.11 (2.57)	1.49 (1.79)	0.011	3.22 (3.05)	2.60 (2.59)	0.003	3.82 (2.89)	2.77 (2.81)	<0.001	1.40 (1.65)	1.11 (1.40)	0.4	**0.008**
Missing	1	0		0	1											0	6		
DBP Z-Score	-0.41 (0.61)	0.18 (0.76)	<0.001	0.48 (1.09)	0.89 (0.74)	<0.001	-0.11 (0.65)	0.60 (0.87)	<0.001	0.13 (0.81)	-0.22 (0.72)	<0.001	-0.03 (0.59)	-0.20 (0.64)	0.022	-0.29 (1.04)	-0.01 (0.73)	0.092	**<0.001**
Missing							0	28											
SBP Z-Score	-0.36 (0.97)	0.37 (0.87)	<0.001	0.52 (1.10)	0.69 (0.85)	0.2	0.62 (0.77)	0.19 (0.96)	0.006	0.63 (0.87)	-0.79 (0.85)	<0.001	-0.44 (0.77)	0.01 (0.84)	<0.001	0.13 (1.09)	0.04 (0.82)	0.6	**<0.001**
Missing							0	28											
BMI Z-Score	0.47 (1.32)	0.81 (1.29)	0.006	0.14 (1.12)	0.58 (1.35)	<0.001	0.10 (1.15)	0.03 (1.27)	0.5	0.70 (1.21)	0.40 (1.13)	<0.001	0.38 (1.11)	0.49 (1.17)	0.12	0.01 (0.69)	-0.09 (0.88)	0.3	**<0.001**
Missing										0	2								
Ever Asthma:			0.3			0.11			>0.9			0.4			>0.9			0.2	**0.002**
Healthy	72 (94%)	63 (89%)		95 (82%)	84 (73%)		64 (84%)	64 (84%)		148 (95%)	141 (93%)		103 (93%)	103 (93%)		48 (96%)	45 (90%)		
Altered	5 (6.5%)	8 (11%)		21 (18%)	31 (27%)		12 (16%)	12 (16%)		8 (5.1%)	11 (7.2%)		8 (7.2%)	8 (7.2%)		2 (4.0%)	5 (10%)		
Missing	0	6		0	1					0	4								
Asthma Medication:			0.001			0.4			>0.9			<0.001			0.007			0.2	**<0.001**
Healthy	53 (69%)	64 (90%)		88 (76%)	93 (81%)		67 (88%)	67 (88%)		133 (85%)	143 (96%)		98 (88%)	108 (97%)		49 (98%)	46 (92%)		
Altered	24 (31%)	7 (9.9%)		28 (24%)	22 (19%)		9 (12%)	9 (12%)		23 (15%)	6 (4.0%)		13 (12%)	3 (2.7%)		1 (2.0%)	4 (8.0%)		
Missing	0	6		0	1					0	7								
Rhinitis:			0.6			0.4			0.11			0.4			0.7			0.8	**0.003**
Healthy	60 (78%)	58 (82%)		87 (75%)	81 (70%)		57 (75%)	48 (63%)		128 (82%)	122 (78%)		69 (62%)	72 (65%)		43 (86%)	42 (84%)		
Altered	17 (22%)	13 (18%)		29 (25%)	34 (30%)		19 (25%)	28 (37%)		28 (18%)	34 (22%)		42 (38%)	39 (35%)		7 (14%)	8 (16%)		
Missing	0	6		0	1														
Dermatitis:			0.4			0.5			0.047			0.15			0.4			0.4	0.075
Healthy	62 (81%)	53 (75%)		90 (78%)	93 (81%)		59 (78%)	68 (89%)		104 (67%)	112 (74%)		89 (80%)	94 (85%)		40 (80%)	43 (86%)		

Altered	15 (19%)	18 (25%)		26 (22%)	22 (19%)		17 (22%)	8 (11%)		52 (33%)	39 (26%)		22 (20%)	17 (15%)		10 (20%)	7 (14%)		
Missing	0	6		0	1					0	5								
Wheezing:			0.8			0.036			0.4			0.015			0.007			0.2	**<0.001**
Healthy	73 (95%)	68 (96%)		80 (69%)	93 (81%)		68 (89%)	71 (93%)		139 (89%)	150 (96%)		96 (86%)	107 (96%)		49 (98%)	46 (92%)		
Altered	4 (5.2%)	3 (4.2%)		36 (31%)	22 (19%)		8 (11%)	5 (6.6%)		17 (11%)	6 (3.8%)		15 (14%)	4 (3.6%)		1 (2.0%)	4 (8.0%)		
Missing	0	6		0	1														

Descriptive statistics are presented as mean (standard deviation) or n (%). [1] *Within group P-values were obtained from paired t-test analysis for continuous outcomes and from McNemar's test for binary outcomes.* [2] *Between group P-values were obtained from ANOVA on deltas*

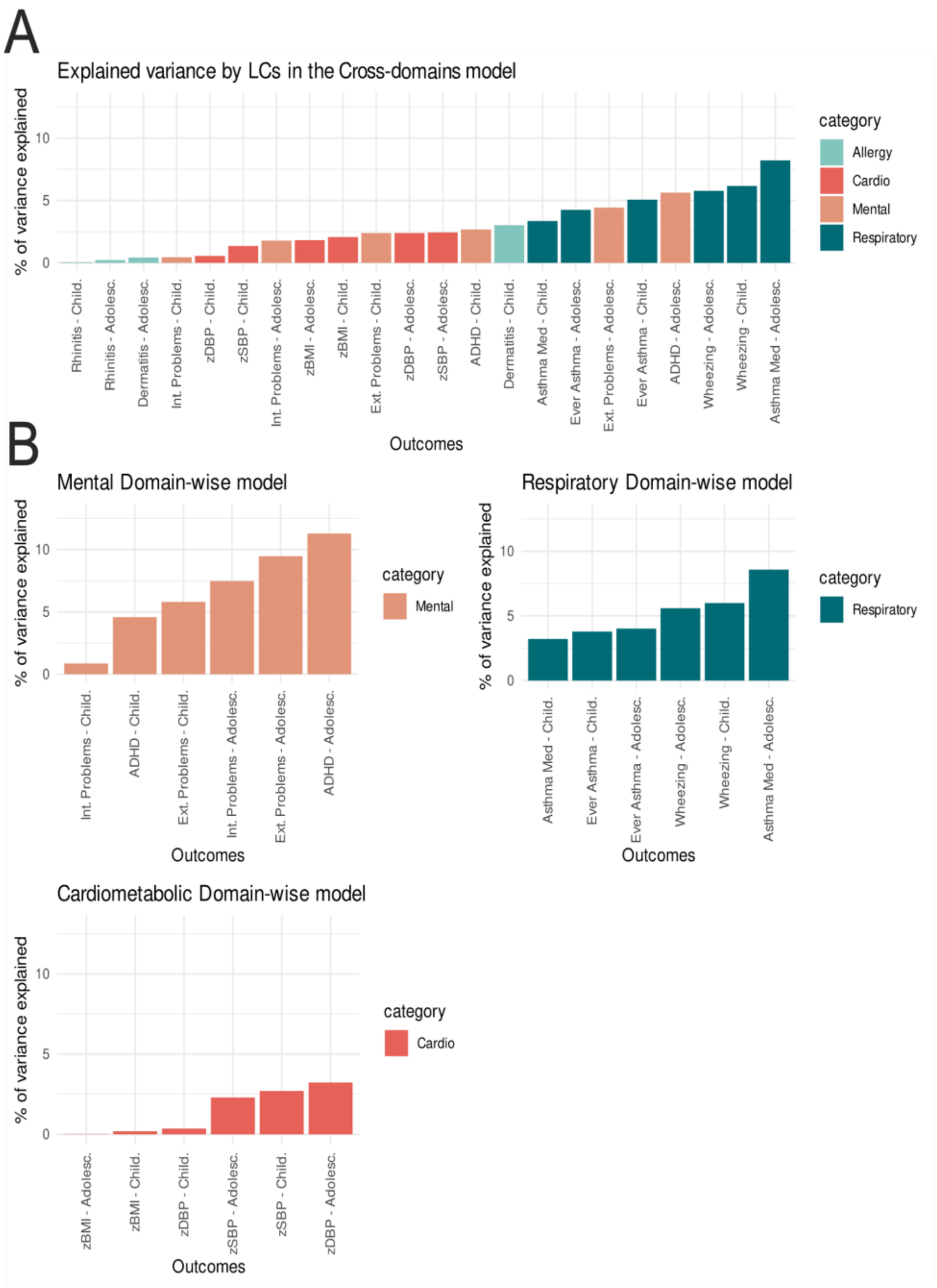

Supplementary Figure 13. Percentage of explained variance by Latent Components for each health outcome in the pregnancy exposome models. *Panel (**A**) refers to the msRRR cross-domains approach (overall health), panels (**B**) refers to the msRRR domain-wise Cardiometabolic, domain-wise Mental health, and Domain-wise Respiratory health. These Latent Components are built based not only in health outcomes correlation structure but also in their relation with exposures, therefore, these LCs summarize as well the exposome-outcomes relationship. Domain-wise approach for allergy trait is not represented since no latent factors were identified by the method.*

Supplementary Table 16. Explained variances for each health outcome and domain in the pregnancy exposome models.

	Averaged Explained Variance in the Respiratory health Domain	Averaged Explained Variance in the Allergy Domain	Averaged Explained Variance in Binary outcomes	Cumulative Explained Variance in Binary outcomes	% of explained variance by latent component (exclusive) In Binary outcomes	Averaged Explained Variance in the Cardiometabolic Domain	Averaged Explained Variance in the Mental health Domain	Averaged Explained Variance in Continuous outcomes	Cumulative Explained Variance in Continuous outcomes	% of explained variance by latent component (exclusive) in continuous outcomes
**Cross-domain outcome-wide approach**										
LC1	2.66	0.44	1.77	1.77	48.37	0.53	1.18	0.85	0.85	36.88
LC2	0.24	0.06	0.17	1.94	4.70	0.73	0.92	0.82	1.68	34.66
LC3	2.58	0.43	1.72	**3.66**	46.92	0.51	0.80	0.66	**2.34**	28.46
Cumulative	**5.48**	**0.94**	-	-	100	**1.78**	**2.90**	-	-	100
**Domain-wise outcome-wide approaches**										
Cumulative by all LCs	**5.20**	-	-	-	-	**1.47**	**6.58**	-	-	-

Abbreviations: LCs; Latent Components.

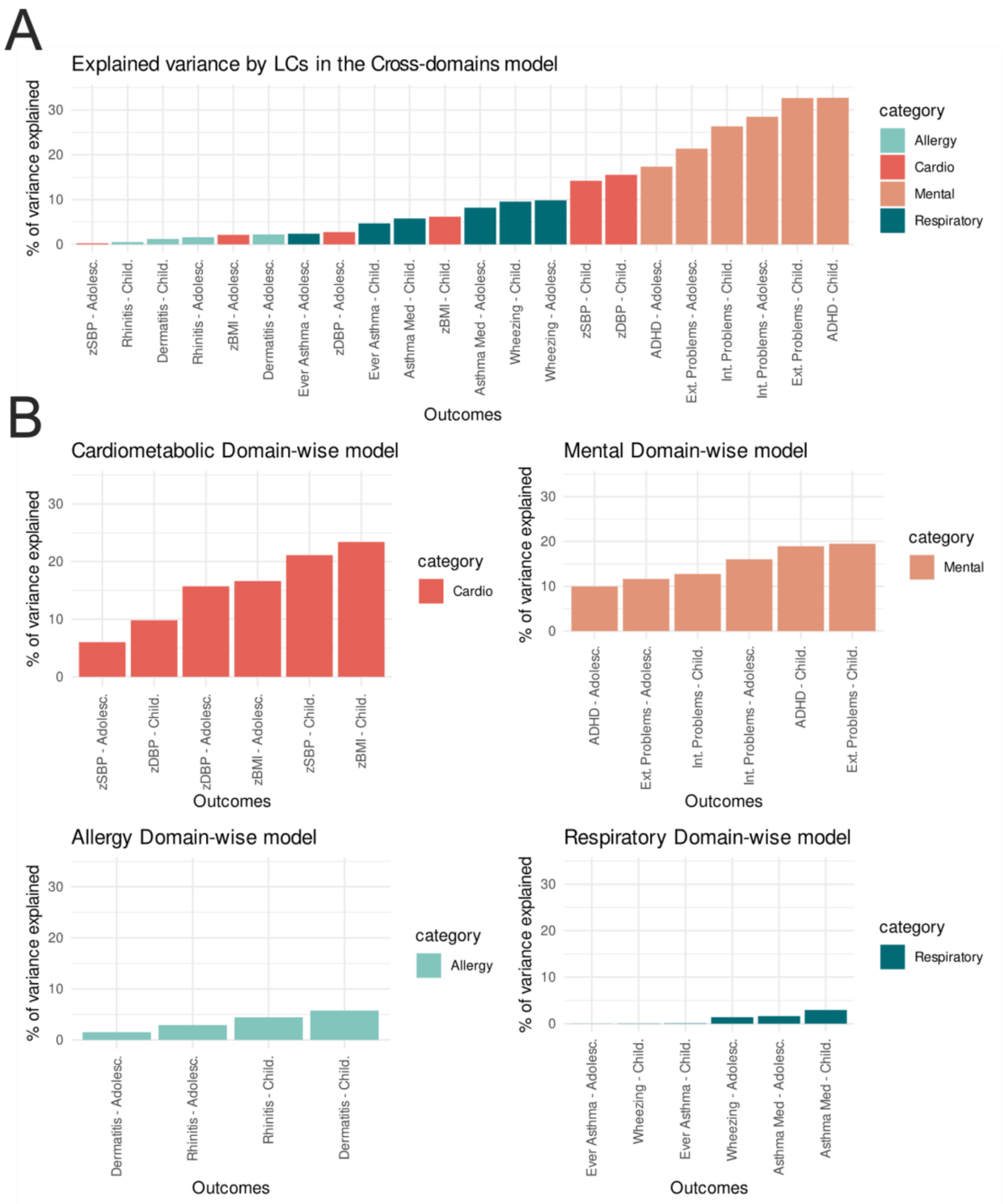

Supplementary Figure 14. Percentage of explained variance for each health outcome in the childhood exposome models. *Panel (**A**) refers to the msRRR cross-domains approach (overall health), panel (**B**) refers to the msRRR domain-wise Cardiometabolic, domain-wise Mental health, Domain-wise Respiratory health and Domain-wise Allergy.*

Table S17. Overall results from msRRR outcome-wide models.

Approach	N Latent Factors (rank)	λ	MSE in Training Set	MSE in Test Set	N Selected exposures
**Pregnancy exposome**					
Cross-domains approach (overall health)	3	0.83	0.87	0.97	2
Domain-wise Cardiometabolic	2	0.99	0.78	0.85	1
Domain-wise Mental health	5	0.60	0.90	0.99	6
Domain-wise Respiratory health	2	0.81	0.92	0.94	1
Domain-wise Allergy	2	0.73	0.93	1.12	0
**Childhood exposome**					
Cross-domains approach (overall health)	4	0.68	0.82	0.90	16
Domain-wise Cardiometabolic	3	0.40	0.68	0.77	32
Domain-wise Mental health	2	0.81	0.85	0.91	4
Domain-wise Respiratory health	2	0.60	0.90	0.96	2
Domain-wise Allergy	3	0.49	0.92	1.05	4

Optimal hyperparameters (N latent factors and lambda penalty) are presented for each model. Predictive model performance in training and test sets are shown based on mean square error. Abbreviations: MSE: Mean squared error.

Supplementary Table 18. Explained variances for each health outcome and domain in the childhood exposome models.

	Averaged Explained Variance in the Respiratory health Domain	Averaged Explained Variance in the Allergy Domain	Averaged Explained Variance in Binary outcomes	Cumulative Explained Variance in Binary outcomes	% of explained variance by latent component (exclusive) In Binary outcomes	Averaged Explained Variance in the Cardiometabolic Domain	Averaged Explained Variance in the Mental health Domain	Averaged Explained Variance in Continuous outcomes	Cumulative Explained Variance in Continuous outcomes	% of explained variance by latent component (exclusive) in continuous outcomes
**Cross-domain outcome-wide approach**										
LC1	3.16	0.87	2.24	2.24	48.73	1.23	4.44	2.84	2.84	16.97
LC2	1.39	0.23	0.93	3.17	20.16	1.91	5.61	3.76	6.60	22.61
LC3	0.49	0.11	0.34	3.51	7.41	1.11	7.65	4.38	10.97	26.28
LC4	1.70	0.18	1.09	**4.60**	23.71	2.58	8.79	5.69	**16.66**	34.14
Cumulative	**6.74**	**1.39**	-	-	100	**6.84**	**26.48**	-	-	100
**Domain-wise outcome-wide approaches**										
Cumulative by all LCs	**1.02**	**3.65**	-	-	-	**15.45**	**14.80**	-	-	-

Abbreviations: LCs; Latent Components.

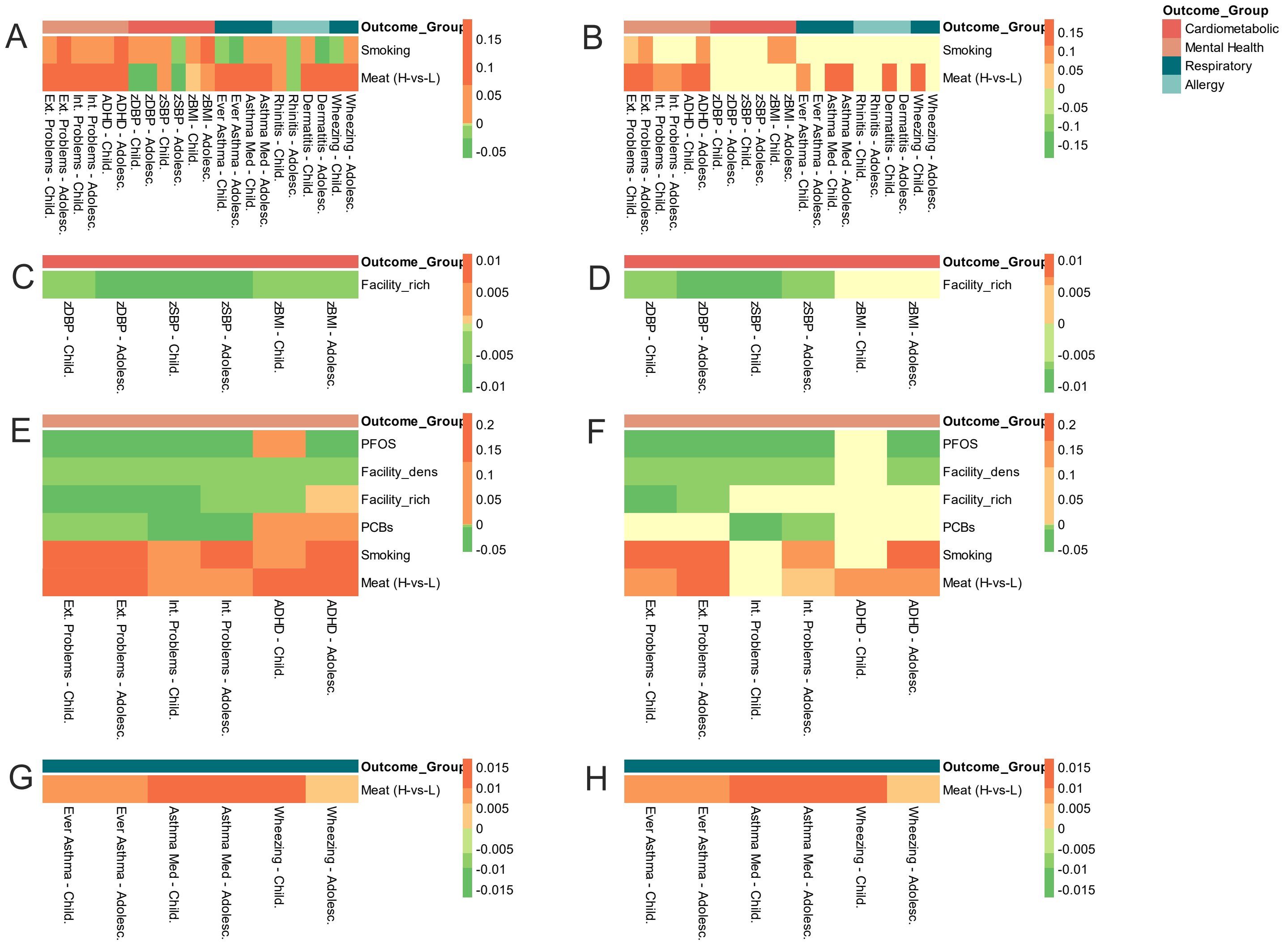

Supplementary Figure 15. Estimated coefficients for each exposure-outcome pair association from msRRR models with pregnancy exposome. *Panels on the left refer to raw msRRR results, while panels on the right are the ones obtained after bootstrapping. Panels (**A-B**) refer to the cross-domains model (approach 1), panels (**C-D**) refer to the cardiometabolic domain-wise model, panels (**E-F**) refer to the mental health domain-wise model, and panels (**G-H**) refer to the respiratory domain-wise model. No exposure was selected in the allergy domain-wise model. Left-column heatmaps show results without bootstrap resampling; right-column heatmaps show results with 500 bootstrap iterations to derive empirical p-values, adding an intra-exposure sparsity layer.*

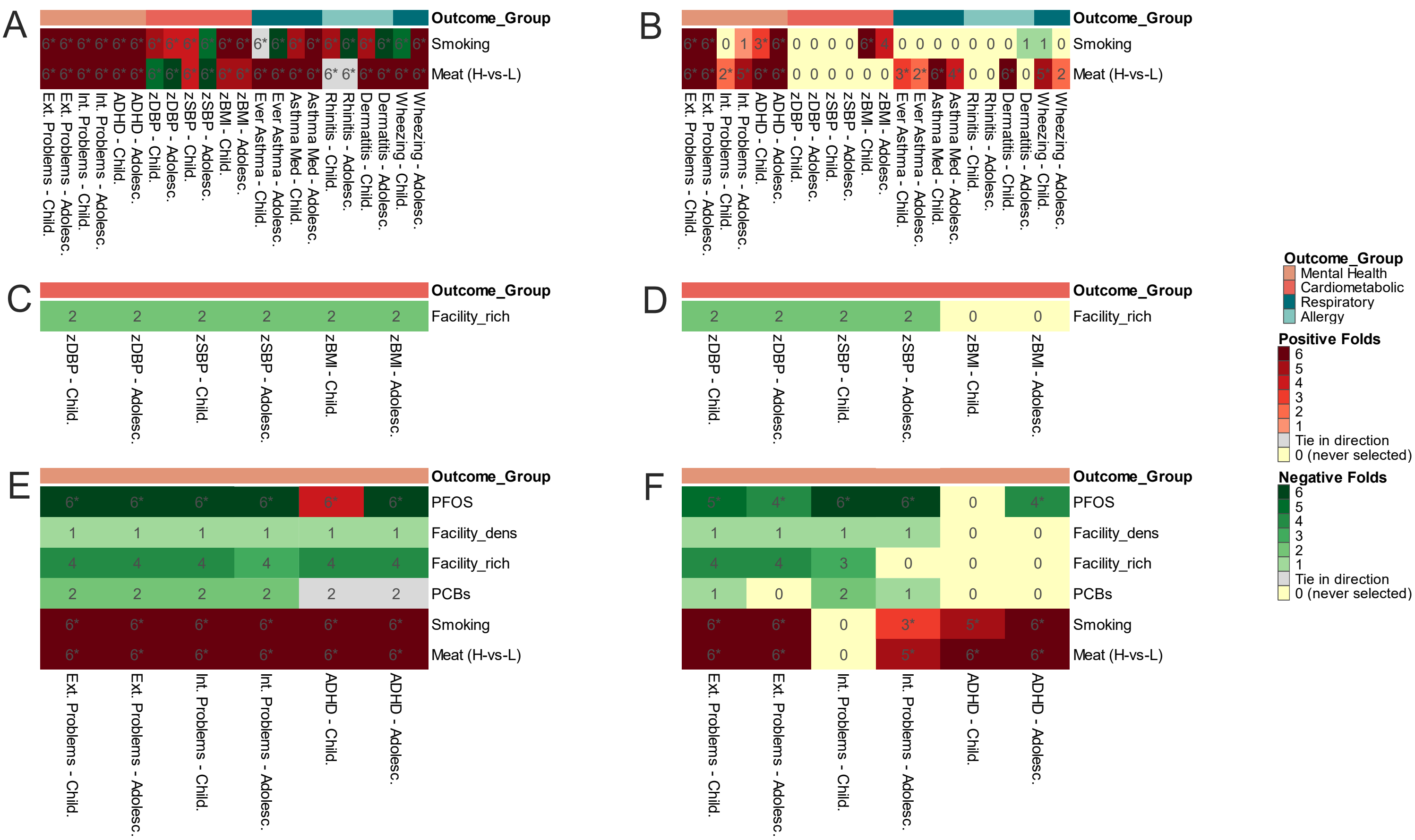

Supplementary Figure 16. Leave-one-cohort-out (LOCO) sensitivity analysis of outcome-wide associations for pregnancy exposures. *Summary heatmaps from the leave-one-cohort-out (LOCO) sensitivity analysis applied to all msRRR models using the pregnancy exposure dataset. Each model was re-estimated six times, systematically excluding one of the six HELIX cohorts per iteration (BiB, EDEN, INMA, KANC, MoBa, RHEA). Panels (**A-B**) refer to the cross-domains model (approach 1), panels (**C-D**) refer to the cardiometabolic domain-wise model, panels (**E-F**) refer to the mental health domain-wise model, no exposure was selected in the respiratory and allergy domain-wise models. For each exposure–outcome pair, the number shown in each cell indicates in how many of the six LOCO iterations the association remained selected after penalization (range: 0–6). Left-column heatmaps show results without bootstrap resampling; right-column heatmaps show results with 500 bootstrap iterations to derive empirical p-values, adding an intra-exposure sparsity layer. Cell color reflects the direction of the association — not its magnitude — following the same palette as figure S15: red indicates increased health risk, green indicates a protective association. Darker shading reflects a higher number of LOCO iterations supporting the association in that direction. Cells shown in grey indicate directional ties across iterations (i.e., no consensus on effect sign). Cells showing 0 indicate that the exposure–outcome pair was never selected. An asterisk (*) denotes cross-regional robustness: the association was selected in at least one LOCO iteration from both the South-Mediterranean block (INMA, RHEA) and the North-Western European block (MoBa, KANC, BiB, EDEN), confirming generalizability across distinct European socio-environmental contexts. Associations meeting both the frequency criterion (>4 of 6 folds) and the cross-regional criterion (asterisk present) were considered robust and are discussed in the main text. Abbreviations: msRRR, mixed-responses sparse reduced-rank regression; LOCO, leave-one-cohort-out; PFOS, perfluorooctane sulfonate; PCBs, polychlorinated biphenyls; Facility_dens, facility density within 300m buffer; Facility_rich, facility richness within 300m buffer; H-vs-L, high vs. low intake tertile.*

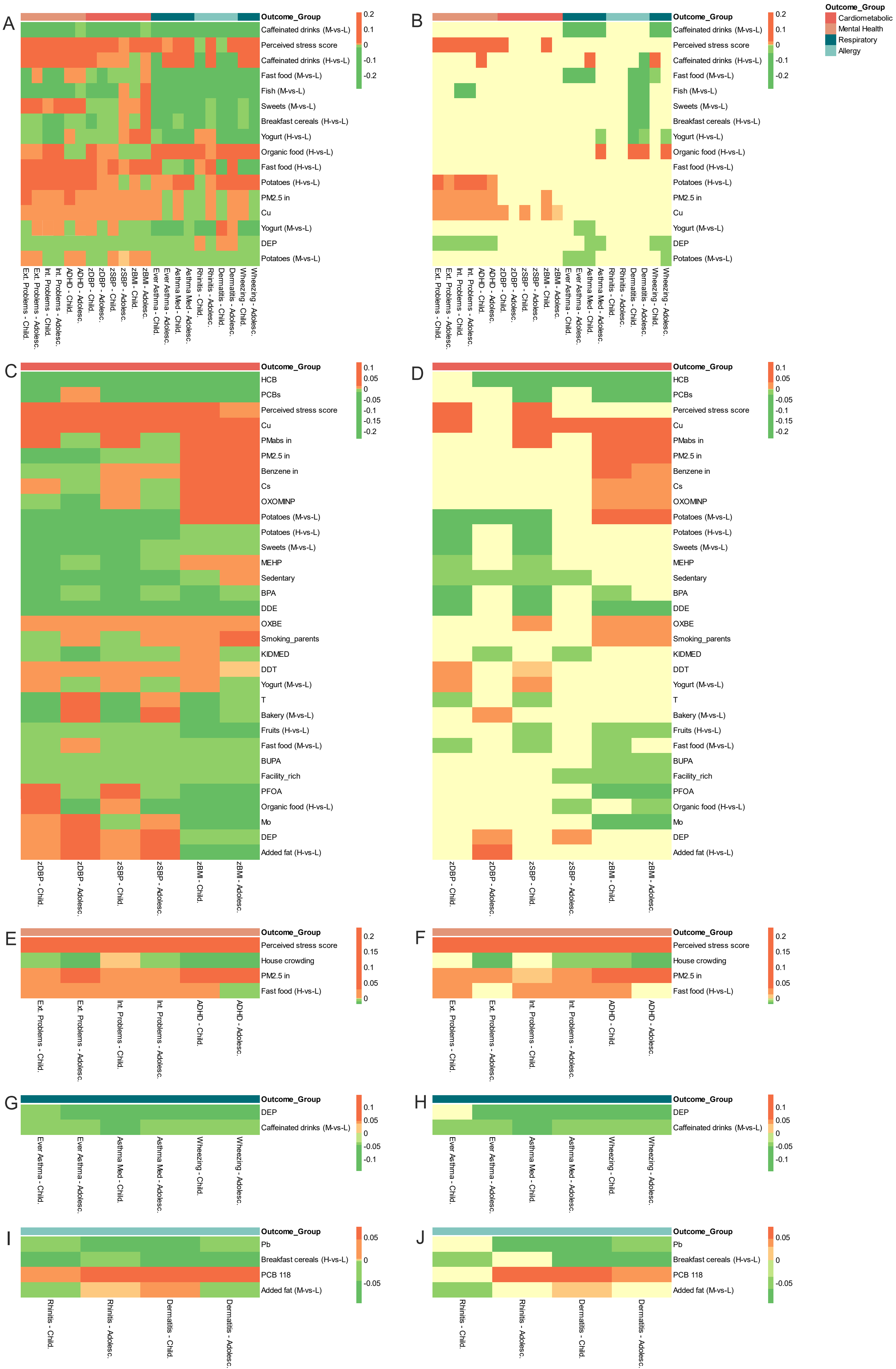
A
B
Outcome_Group
Cardiometabolic
Mental Health
Respiratory
Allergy
0.2
0.1
0
-0.1
-0.2
Caffeinated drinks (M-vs-L)
Perceived stress score
Caffeinated drinks (H-vs-L)
Fast food (M-vs-L)
Fish (M-vs-L)
Sweets (M-vs-L)
Breakfast cereals (H-vs-L)
Yogurt (H-vs-L)
Organic food (H-vs-L)
Fast food (H-vs-L)
Potatoes (H-vs-L)
PM2.5 in
Cu
Yogurt (M-vs-L)
DEP
Potatoes (M-vs-L)
Ext. Problems - Child.
Ext. Problems - Adolesc.
Int. Problems - Child.
Int. Problems - Adolesc.
ADHD - Child.
ADHD - Adolesc.
zDBP - Child.
zDBP - Adolesc.
zSBP - Child.
zSBP - Adolesc.
zBMI - Child.
zBMI - Adolesc.
Ever Asthma - Child.
Ever Asthma - Adolesc.
Asthma Med - Child.
Asthma Med - Adolesc.
Rhinitis - Child.
Rhinitis - Adolesc.
Dermatitis - Child.
Dermatitis - Adolesc.
Wheezing - Child.
Wheezing - Adolesc.
C
D
0.1
0.05
0
-0.05
-0.1
-0.15
-0.2
HCB
PCBs
Perceived stress score
Cu
PMabs in
PM2.5 in
Benzene in
Cs
OXOMINP
Potatoes (M-vs-L)
Potatoes (H-vs-L)
Sweets (M-vs-L)
MEHP
Sedentary
BPA
DDE
OXBE
Smoking_parents
KIDMED
DDT
Yogurt (M-vs-L)
T
Bakery (M-vs-L)
Fruits (H-vs-L)
Fast food (M-vs-L)
BUPA
Facility_rich
PFOA
Organic food (H-vs-L)
Mo
DEP
Added fat (H-vs-L)
zDBP - Child.
zDBP - Adolesc.
zSBP - Child.
zSBP - Adolesc.
zBMI - Child.
zBMI - Adolesc.
E
F
0.2
0.15
0.1
0.05
0
Perceived stress score
House crowding
PM2.5 in
Fast food (H-vs-L)
Ext. Problems - Child.
Ext. Problems - Adolesc.
Int. Problems - Child.
Int. Problems - Adolesc.
ADHD - Child.
ADHD - Adolesc.
G
H
0.1
0.05
0
-0.05
-0.1
DEP
Caffeinated drinks (M-vs-L)
Ever Asthma - Child.
Ever Asthma - Adolesc.
Asthma Med - Child.
Asthma Med - Adolesc.
Wheezing - Child.
Wheezing - Adolesc.
I
J
0.05
0
-0.05
Pb
Breakfast cereals (H-vs-L)
PCB 118
Added fat (M-vs-L)
Rhinitis - Child.
Rhinitis - Adolesc.
Dermatitis - Child.
Dermatitis - Adolesc.

Supplementary Figure 17. Estimated coefficients for each exposure-outcome pair association from msRRR models with early-childhood exposome. *Panels on the left refer to raw msRRR results, while panels on the right are the ones obtained after bootstrapping. Panels (**A-B**) refer to the cross-domains model (approach 1), panels (**C-D**) refer to the cardiometabolic domain-wise model, panels (**E-F**) refer to the mental health domain-wise model, panels (**G-H**) refer to the respiratory domain-wise model, and panels (**I-J**) refer to the allergy domain-wise model. Left-column heatmaps show results without bootstrap resampling; right-column heatmaps show results with 500 bootstrap iterations to derive empirical p-values, adding an intra-exposure sparsity layer.*

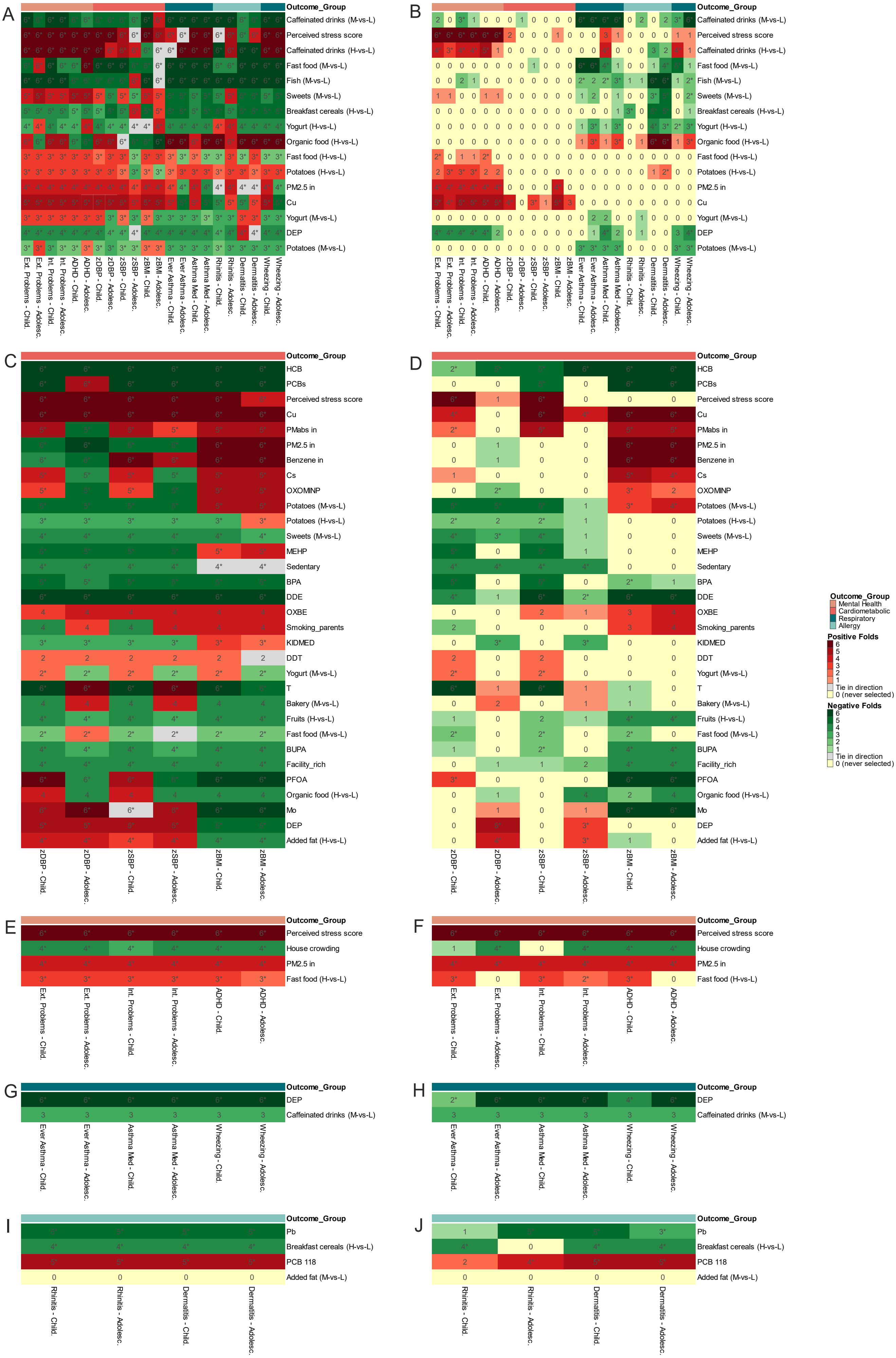
A
B
Outcome_Group
Caffeinated drinks (M-vs-L)
Perceived stress score
Caffeinated drinks (H-vs-L)
Fast food (M-vs-L)
Fish (M-vs-L)
Sweets (M-vs-L)
Breakfast cereals (H-vs-L)
Yogurt (H-vs-L)
Organic food (H-vs-L)
Fast food (H-vs-L)
Potatoes (H-vs-L)
PM2.5 in
Cu
Yogurt (M-vs-L)
DEP
Potatoes (M-vs-L)
Ext. Problems - Child.
Ext. Problems - Adolesc.
Int. Problems - Child.
Int. Problems - Adolesc.
ADHD - Child.
ADHD - Adolesc.
zDBP - Child.
zDBP - Adolesc.
zSBP - Child.
zSBP - Adolesc.
zBMI - Child.
zBMI - Adolesc.
Ever Asthma - Child.
Ever Asthma - Adolesc.
Asthma Med - Child.
Asthma Med - Adolesc.
Rhinitis - Child.
Rhinitis - Adolesc.
Dermatitis - Child.
Dermatitis - Adolesc.
Wheezing - Child.
Wheezing - Adolesc.
C
D
HCB
PCBs
PMabs in
Benzene in
Cs
OXOMINP
Sweets (M-vs-L)
MEHP
Sedentary
BPA
DDE
OXBE
Smoking_parents
KIDMED
DDT
T
Bakery (M-vs-L)
Fruits (H-vs-L)
BUPA
Facility_rich
PFOA
Mo
Added fat (H-vs-L)
E
F
House crowding
G
H
I
J
Pb
PCB 118
Added fat (M-vs-L)
Outcome_Group
Mental Health
Cardiometabolic
Respiratory
Allergy
Positive Folds
Tie in direction
0 (never selected)
Negative Folds

Supplementary Figure 18. Leave-one-cohort-out (LOCO) sensitivity analysis of outcome-wide associations for childhood exposures. *Summary heatmaps from the leave-one-cohort-out (LOCO) sensitivity analysis applied to all msRRR models using the childhood exposure dataset. Design and interpretation follow the same conventions as Supplementary Figure S16 (prenatal). Each model was re-estimated six times excluding one HELIX cohort per iteration (BiB, EDEN, INMA, KANC, MoBa, RHEA). Panels on the left refer to raw msRRR results, while panels on the right are the ones obtained after bootstrapping. Panels (**A-B**) refer to the cross-domains model (approach 1), panels (**C-D**) refer to the cardiometabolic domain-wise model, panels (**E-F**) refer to the mental health domain-wise model, panels (**G-H**) refer to the respiratory domain-wise model, and panels (**I-J**) refer to the allergy domain-wise model. Left-column heatmaps show results without bootstrap resampling; right-column heatmaps show results with 500 bootstrap iterations to derive empirical p-values, adding an intra-exposure sparsity layer. Cell color reflects the direction of the association — not its magnitude — following the same palette as figure S17: red indicates increased health risk, green indicates a protective association. Darker shading reflects a higher number of LOCO iterations supporting the association in that direction. Cells shown in grey indicate directional ties across iterations (i.e., no consensus on effect sign). Cells showing 0 indicate that the exposure–outcome pair was never selected. An asterisk (*) denotes cross-regional robustness: the association was selected in at least one LOCO iteration from both the South-Mediterranean block (INMA, RHEA) and the North-Western European block (MoBa, KANC, BiB, EDEN), confirming generalizability across distinct European socio-environmental contexts. Associations meeting both the frequency criterion (>4 of 6 folds) and the cross-regional criterion (asterisk present) were considered robust and are discussed in the main text. Abbreviations: msRRR, mixed-responses sparse reduced-rank regression; LOCO, leave-one-cohort-out; $PM_{2.5}$, fine particulate matter (indoor); PMabs, particulate matter absorbance; HCB, hexachlorobenzene; PCBs, polychlorinated biphenyls; DDE, dichlorodiphenyldichloroethylene; DDT, dichlorodiphenyltrichloroethane; DEP, diethyl phosphate; MEHP, mono(2-ethylhexyl) phthalate; OXBE, monobutyl phthalate oxidative metabolite; oxo-MiNP, oxo-mono-isononyl phthalate; BPA, bisphenol A; PFOA, perfluorooctanoic acid; BUPA, mono-n-butyl phthalate; KIDMED, Mediterranean diet adherence index; M-vs-L / H-vs-L, medium or high vs. low intake tertile.*

Besides outcome-wide analyses, we conducted an additional step. We implemented a Gaussian graphical Lasso model with an additional step for stability selection to construct sparse partial correlation networks linking exposures and health outcomes. Specifically, we applied the SHARP framework (stability of selection approach based on re-sampling procedures (105), which controls for multiplicity and selects only robust pairwise associations.

A complementary circular network plots is provided to visualize the interdependencies within the exposome-health architecture (**Figure S19**). It presents a global network linking prenatal and childhood exposures directly to individual health outcomes across both developmental stages (childhood and adolescence). This visualization confirms several key observations: 1) a distinct temporal density pattern, where childhood exposures show a higher degree of connectivity with adolescent outcomes, while prenatal exposures are mostly connected with childhood outcomes, aligning with the greater explained variance observed for the childhood exposome in our primary msRRR models; 2) outcome-time window accumulation, as adolescent health measures aggregate more connections than those in childhood, reflecting the persistence and potential latency of environmental impacts over time; 3) robustness against confounding, no worrying pattern of confounders directly driving outcomes (beyond expected cohort-structure) was observed, providing reassurance against major selection and confounding biases; and 4) validation of 'master regulators of health', where exposures identified as highly influential in our main analysis (e.g., perceived stress, dietary patterns, and indoor $PM_{2.5}$) emerge as the most highly connected nodes (hubs) in the network.

Interestingly, we also observed minimal cross-temporal connectivity between pregnancy and childhood exposures, with the exception of stable built environment features, underscoring that these periods represent distinct etiological windows. This lack of correlation reinforces the importance of longitudinal exposome characterization, as childhood exposures do not merely proxy prenatal conditions but contribute independent and cumulative risk to the health trajectory.

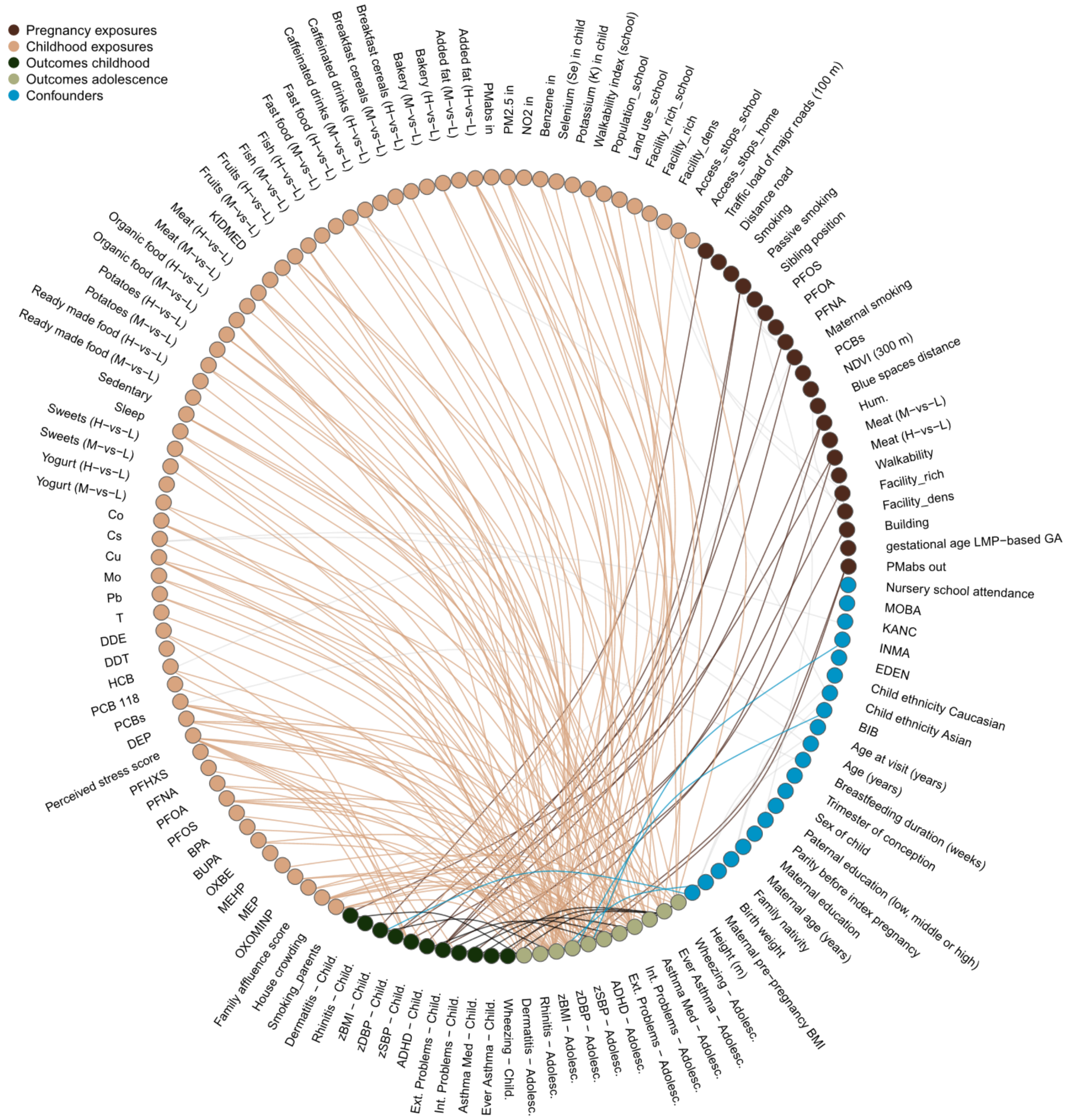

Supplementary Figure 19. Sparse partial correlation network for childhood and pregnancy exposures and individual health outcomes at childhood and adolescence.

In summary, this network analysis further illuminates the architecture of exposome–health relationships, confirming the temporal logic of our longitudinal design: childhood exposures accumulate more connections with adolescent outcomes than prenatal exposures, and the minimal cross-temporal connectivity between the two windows confirms that they represent genuinely distinct etiological environments. Importantly however, the network approach yields a dense, poorly sparse solution susceptible to false positives from co-exposure correlations — it therefore complements rather than replaces our penalized outcome-wide framework, which is specifically designed to produce sparse, interpretable associations under rigorous statistical control.

REFERENCES

1. P. D. Gluckman, M. A. Hanson, "The developmental origins of health and disease" in *Early Life Origins of Health and Disease* (Springer, 2006), vol. 573, pp. 1–7.

2. C. Wagner, C. Carmeli, J. Jackisch, M. Kivimäki, B. W. A. van der Linden, S. Cullati, A. Chiolero, Life course epidemiology and public health. *Lancet Public Health* **9**, e261–e269 (2024).

3. R. Vermeulen, E. L. Schymanski, A. L. Barabási, G. W. Miller, The exposome and health: Where chemistry meets biology. *Science* **367**, 392–396 (2020).

4. G. W. Miller, Banbury Exposomics Consortium, Integrating exposomics into biomedicine. *Science* **388**, 356–358 (2025).

5. M. Fang, L. Hu, D. Chen, Y. Guo, J. Liu, C. Lan, J. Gong, B. Wang, Exposome in human health: Utopia or wonderland? *Innovation* **2**, 100172 (2021).

6. J. N. Weiss, A. Karma, W. R. MacLellan, M. Deng, C. D. Rau, C. M. Rees, J. Wang, N. Wisniewski, E. Eskin, S. Horvath, Z. Qu, Y. Wang, A. J. Lusis, "Good enough solutions" and the genetics of complex diseases. *Circ. Res.* **111**, 493–504 (2012).

7. S. L. Prescott, Early-life environmental determinants of allergic diseases and the wider pandemic of inflammatory noncommunicable diseases. *J. Allergy Clin. Immunol.* **131**, 23–30 (2013).

8. GBD 2019 Diseases and Injuries Collaborators, Global burden of 369 diseases and injuries in 204 countries and territories, 1990–2019: A systematic analysis for the Global Burden of Disease Study 2019. *Lancet* **396**, 1204–1222 (2020).

9. G. Banderali, A. Martelli, M. Landi, F. Moretti, F. Betti, G. Radaelli, C. Lassandro, E. Verduci, Short and long term health effects of parental tobacco smoking during pregnancy and lactation: A descriptive review. *J. Transl. Med.* **13**, 327 (2015).

10. L. Agier, X. Basagaña, L. Maitre, B. Granum, P. K. Bird, M. Casas, B. Oftedal, J. Wright, S. Andrusaityte, M. de Castro, E. Cequier, L. Chatzi, D. Donaire-Gonzalez, R. Grazuleviciene, L. S. Haug, A. K. Sakhi, V. Leventakou, R. McEachan, M. Nieuwenhuijsen, I. Petraviciene, O. Robinson, T. Roumeliotaki, J. Sunyer, I. Tamayo-Uria, C. Thomsen, J. Urquiza, A. Valentin, R. Slama, M. Vrijheid, V. Siroux, Early-life exposome and lung function in children in Europe: An analysis of data from the longitudinal, population-based HELIX cohort. *Lancet Planet. Health* **3**, e81–e92 (2019).

11. S. Santos, L. Maitre, C. Warembourg, L. Agier, L. Richiardi, X. Basagaña, M. Vrijheid, Applying the exposome concept in birth cohort research: A review of statistical approaches. *Eur. J. Epidemiol.* **35**, 193–204 (2020).

12. C. Warembourg, A. Anguita-Ruiz, V. Siroux, R. Slama, M. Vrijheid, L. Richiardi, X. Basagaña, Statistical approaches to study exposome-health associations in the context of repeated exposure data: A simulation study. *Environ. Sci. Technol.* **57**, 16232–16243 (2023).

13. T. J. VanderWeele, M. B. Mathur, Y. Chen, Outcome-wide longitudinal designs for causal inference: A new template for empirical studies. *Statist. Sci.* **35**, 437–466 (2020).

14. T. J. Vanderweele, Outcome-wide Epidemiology. *Epidemiology* **28**, 399–402 (2017).

15. Z. Cao, C. Xu, P. Zhang, Y. Wang, Associations of sedentary time and physical activity with adverse health conditions: Outcome-wide analyses using isotemporal substitution model. *EClinicalMedicine* **48**, 101424 (2022).

16. I. Amine, A. Guillien, C. Philippat, A. Anguita-Ruiz, M. Casas, M. de Castro, A. Dedele, J. Garcia-Aymerich, B. Granum, R. Grazuleviciene, B. Heude, L. S. Haug, J. Julvez, M. López-Vicente, L. Maitre, R. McEachan, M. Nieuwenhuijsen, N. Stratakis, M. Vafeiadi, J. Wright, T. Yang, W. L. Yuan, X. Basagaña, R. Slama, M. Vrijheid, V. Siroux, Environmental exposures in early-life and general health in childhood. Research Square [Preprint] (2023). https://doi.org/10.21203/rs.3.rs-2640215/v1.

17. A. Anguita-Ruiz, I. Amine, N. Stratakis, L. Maitre, J. Julvez, J. Urquiza, C. Luo, M. Nieuwenhuijsen, C. Thomsen, R. Grazuleviciene, B. Heude, R. McEachan, M. Vafeiadi, L. Chatzi, J. Wright, T. C. Yang, R. Slama, V. Siroux, M. Vrijheid, X. Basagaña, Beyond the single-outcome approach: A comparison of outcome-wide analysis methods for exposome research. *Environ. Int.* **182**, 108344 (2023).

18. C. Luo, J. Liang, G. Li, F. Wang, C. Zhang, D. K. Dey, K. Chen, Leveraging mixed and incomplete outcomes via reduced-rank modeling. *J. Multivar. Anal.* **167**, 378–394 (2018).

19. L. Maitre, J. De Bont, M. Casas, O. Robinson, G. M. Aasvang, L. Agier, S. Andrušaitytė, F. Ballester, X. Basagaña, E. Borràs, C. Brochot, M. Bustamante, A. Carracedo, M. De Castro, A. Dedele, D. Donaire-Gonzalez, X. Estivill, J. Evandt, S. Fossati, L. Giorgis-Allemand, J. R. Gonzalez, B. Granum, R. Grazuleviciene, K. B. Gützkow, L. S. Haug, C. Hernandez-Ferrer, B. Heude, J. Ibarluzea, J. Julvez, M. Karachaliou, H. C. Keun, N. H. Krog, C. H. E. Lau, V. Leventakou, S. Lyon-Caen, C. Manzano, D. Mason, R. McEachan, H. M. Meltzer, I. Petraviciene, J. Quentin, T. Roumeliotaki, E. Sabido, P. J. Saulnier, A. P. Siskos, V. Siroux, J. Sunyer, I. Tamayo, J. Urquiza, M. Vafeiadi, D. Van Gent, M. Vives-Usano, D. Waiblinger, C. Warembourg, L. Chatzi, M. Coen, P. Van Den Hazel, M. J. Nieuwenhuijsen, R. Slama, C. Thomsen, J. Wright, M. Vrijheid, Human Early Life Exposome (HELIX) study: A European population-based exposome cohort. *BMJ Open* **8**, e021311 (2018).

20. M. Vrijheid, S. Fossati, L. Maitre, S. Márquez, T. Roumeliotaki, L. Agier, S. Andrusaityte, S. Cadiou, M. Casas, M. de Castro, A. Dedele, D. Donaire-Gonzalez, R. Grazuleviciene, L. S. Haug, R. McEachan, H. M. Meltzer, E. Papadopouplou, O. Robinson, A. K. Sakhi, V. Siroux, J. Sunyer, P. E. Schwarze, I. Tamayo-Uria, J. Urquiza, M. Vafeiadi, A. Valentin, C. Warembourg, J. Wright, M. J. Nieuwenhuijsen, C. Thomsen, X. Basagaña, R. Slama, L. Chatzi, Early-life environmental exposures and childhood obesity: An exposome-wide approach. *Environ. Health Perspect.* **128**, 67009 (2020).

21. J. B. Guimbaud, A. P. Siskos, A. K. Sakhi, B. Heude, E. Sabidó, E. Borràs, H. Keun, J. Wright, J. Julvez, J. Urquiza, K. B. Gützkow, L. Chatzi, M. Casas, M. Bustamante, M. Nieuwenhuijsen, M. Vrijheid, M. López-Vicente, M. de Castro Pascual, N. Stratakis, O. Robinson, R. Grazuleviciene, R. Slama, S. Alemany, X. Basagaña, M. Plantevit, R. Cazabet,

L. Maitre, Machine learning-based health environmental-clinical risk scores in European children. *Commun. Med.* **4**, 98 (2024).

22. C. Warembourg, L. Maitre, I. Tamayo-Uria, S. Fossati, T. Roumeliotaki, G. M. Aasvang, S. Andrusaityte, M. Casas, E. Cequier, L. Chatzi, A. Dedele, J. R. Gonzalez, R. Gražulevičienė, L. S. Haug, C. Hernandez-Ferrer, B. Heude, M. Karachaliou, N. H. Krog, R. McEachan, M. Nieuwenhuijsen, I. Petraviciene, J. Quentin, O. Robinson, A. K. Sakhi, R. Slama, C. Thomsen, J. Urquiza, M. Vafeiadi, J. West, J. Wright, M. Vrijheid, X. Basagaña, Early-life environmental exposures and blood pressure in children. *J. Am. Coll. Cardiol.* **74**, 1317–1328 (2019).

23. B. Granum, B. Oftedal, L. Agier, V. Siroux, P. Bird, M. Casas, C. Warembourg, J. Wright, L. Chatzi, M. de Castro, D. Donaire, R. Grazuleviciene, L. Småstuen Haug, L. Maitre, O. Robinson, I. Tamayo-Uria, J. Urquiza, M. Nieuwenhuijsen, R. Slama, C. Thomsen, M. Vrijheid, Multiple environmental exposures in early-life and allergy-related outcomes in childhood. *Environ. Int.* **144**, 106038 (2020).

24. L. Maitre, J. Julvez, M. López-Vicente, C. Warembourg, I. Tamayo-Uria, C. Philippat, K. B. Gützkow, M. Guxens, S. Andrusaityte, X. Basagaña, M. Casas, M. de Castro, L. Chatzi, J. Evandt, J. R. Gonzalez, R. Gražulevičienė, L. Smastuen Haug, B. Heude, C. Hernandez-Ferrer, M. Kampouri, D. Manson, S. Marquez, R. McEachan, M. Nieuwenhuijsen, O. Robinson, R. Slama, C. Thomsen, J. Urquiza, M. Vafeidi, J. Wright, M. Vrijheid, Early-life environmental exposure determinants of child behavior in Europe: A longitudinal, population-based study. *Environ. Int.* **153**, 106523 (2021).

25. J. Julvez, M. López-Vicente, C. Warembourg, L. Maitre, C. Philippat, K. B. Gützkow, M. Guxens, J. Evandt, S. Andrusaityte, M. Burgaleta, M. Casas, L. Chatzi, M. de Castro, D. Donaire-González, R. Gražulevičienė, C. Hernandez-Ferrer, B. Heude, R. Mceachan, M. Mon-Williams, M. Nieuwenhuijsen, O. Robinson, A. K. Sakhi, N. Sebastian-Galles, R. Slama, J. Sunyer, I. Tamayo-Uria, C. Thomsen, J. Urquiza, M. Vafeiadi, J. Wright, X. Basagaña, M. Vrijheid, Early life multiple exposures and child cognitive function: A multi-centric birth cohort study in six European countries. *Environ. Pollut.* **284**, 117404 (2021).

26. N. Stratakis, A. Anguita-Ruiz, L. Fabbri, L. Maitre, J. R. González, S. Andrusaityte, X. Basagaña, E. Borràs, H. C. Keun, L. Chatzi, D. V. Conti, J. Goodrich, R. Grazuleviciene, L. S. Haug, B. Heude, W. L. Yuan, R. McEachan, M. Nieuwenhuijsen, E. Sabidó, R. Slama, C. Thomsen, J. Urquiza, T. Roumeliotaki, M. Vafeiadi, J. Wright, M. Bustamante, M. Vrijheid, Multi-omics architecture of childhood obesity and metabolic dysfunction uncovers biological pathways and prenatal determinants. *Nat. Commun.* **16**, 654 (2025).

27. M. Vrijheid, X. Basagaña, J. R. Gonzalez, V. W. V. Jaddoe, G. Jensen, H. C. Keun, R. R. C. McEachan, J. Porcel, V. Siroux, M. A. Swertz, C. Thomsen, G. M. Aasvang, S. Andrušaitytė, K. Angeli, D. Avraam, F. Ballester, P. Burton, M. Bustamante, M. Casas, L. Chatzi, C. Chevrier, N. Cingotti, D. Conti, A. Crépet, P. Dadvand, L. Duijts, E. van Enckevort, A. Esplugues, S. Fossati, R. Garlantezec, M. D. G. Roig, R. Grazuleviciene, K. B. Gützkow, M. Guxens, S. Haakma, E. V. S. Hessel, L. Hoyles, E. Hyde, J. Klanova, J. D. van Klaveren, A. Kortenkamp, L. Le Brusquet, I. Leenen, A. Lertxundi, N. Lertxundi, C. Lionis, S. Llop, M. J. Lopez-Espinosa, S. Lyon-Caen, L. Maitre, D. Mason, S. Mathy, E. Mazarico, T. Nawrot, M. Nieuwenhuijsen, R. Ortiz, M. Pedersen, J. Perelló, M. Pérez-Cruz, C. Philippat, P. Piler, C. Pizzi, J. Quentin, L. Richiardi, A. Rodriguez, T. Roumeliotaki, J. M. S. Capote, L. Santiago, S. Santos, A. P. Siskos, K. Strandberg-Larsen, N. Stratakis, J. Sunyer, A. Tenenhaus, M. Vafeiadi, R. C. Wilson, J. Wright, T. Yang, R. Slama, Advancing tools for human early lifecourse exposome research and translation (ATHLETE): Project overview. *Environ. Epidemiol.* **5**, e166 (2021).

28. P. Magnus, C. Birke, K. Vejrup, A. Haugan, E. Alsaker, A. K. Daltveit, M. Handal, M. Haugen, G. Høiseth, G. P. Knudsen, L. Paltiel, P. Schreuder, K. Tambs, L. Vold, C. Stoltenberg, Cohort profile update: The Norwegian mother and child cohort study (MoBa). *Int. J. Epidemiol.* **45**, 382–388 (2016).

29. M. A. Gomez-Llorente, A. Martínez-Cañavate, N. Chueca, M. A. de la Cruz Rico, R. Romero, A. Anguita-Ruiz, C. M. Aguilera, M. Gil-Campos, M. D. Mesa, B. Khakimov, J. A. Morillo, Á. Gil, J. Camacho, C. Gomez-Llorente, A multi-omics approach reveals new signatures in obese allergic asthmatic children. *Biomedicine* **8**, 359 (2020).

30. M. Behl, D. Rao, K. Aagaard, T. L. Davidson, E. D. Levin, T. A. Slotkin, S. Srinivasan, D. Wallinga, M. F. White, V. R. Walker, K. A. Thayer, A. C. Holloway, Evaluation of the association between maternal smoking, childhood obesity, and metabolic disorders: A national toxicology program workshop review. *Environ. Health Perspect.* **121**, 170–180 (2013).

31. E. Oken, E. B. Levitan, M. W. Gillman, Maternal smoking during pregnancy and child overweight: Systematic review and meta-analysis. *Int. J. Obes.* **32**, 201–210 (2007).

32. F. Rice, K. Langley, C. Woodford, G. Davey Smith, A. Thapar, Identifying the contribution of prenatal risk factors to offspring development and psychopathology: What designs to use and a critique of literature on maternal smoking and stress in pregnancy. *Dev. Psychopathol.* **30**, 1107–1128 (2018).

33. N. Baïz, J. Just, J. Chastang, A. Forhan, B. De Lauzon-Guillain, A. M. Magnier, I. Annesi-Maesano, Maternal diet before and during pregnancy and risk of asthma and allergic rhinitis in children. *Allergy Asthma Clin. Immunol.* **15**, (2019).

34. L. Chatzi, R. Garcia, T. Roumeliotaki, M. Basterrechea, H. Begiristain, C. Iñiguez, J. Vioque, M. Kogevinas, J. Sunyer, INMA studay group, RHEA study group, Mediterranean diet adherence during pregnancy and risk of wheeze and eczema in the first year of life: INMA (Spain) and RHEA (Greece) mother–child cohort studies. *Br. J. Nutr.* **110**, 2058–2068 (2013).

35. M. Erkkola, B. I. Nwaru, M. Kaila, C. Kronberg-Kippilä, J. Ilonen, O. Simell, R. Veijola, M. Knip, S. M. Virtanen, Risk of asthma and allergic outcomes in the offspring in relation to maternal food consumption during pregnancy: A Finnish birth cohort study. *Pediatr. Allergy Immunol.* **23**, 186–194 (2012).

36. K. N. Chong, E. H. Chean, R. A. Zaki, S. H. M. Taib, H. A. Majid, A. K. Ng, K. P. Eg, S. Y. Hng, C. S. Ju, N. Razali, A. M. Nathan, J. A. de Bruyne, Maternal diet during pregnancy and infant respiratory morbidity: A prospective study. *Asia Pac. J. Clin. Nutr.* **30**, 632–642 (2021).

37. N. Alvarez Zallo, I. Aguinaga-Ontoso, I. Alvarez-Alvarez, B. Marin-Fernandez, F. Guillén-Grima, C. Azcona-San Julián, Influence of the Mediterranean diet during pregnancy in the

development of wheezing and eczema in infants in Pamplona, Spain. *Allergol. Immunopathol.* **46**, 9–14 (2018).

38. L. Chatzi, M. Torrent, I. Romieu, R. Garcia-Esteban, C. Ferrer, J. Vioque, M. Kogevinas, J. Sunyer, Mediterranean diet in pregnancy is protective for wheeze and atopy in childhood. *Thorax* **63**, 507–513 (2008).

39. J. A. Castro-Rodriguez, M. Ramirez-Hernandez, O. Padilla, R. M. Pacheco-Gonzalez, V. Pérez-Fernández, L. Garcia-Marcos, Effect of foods and Mediterranean diet during pregnancy and first years of life on wheezing, rhinitis and dermatitis in preschoolers. *Allergol. Immunopathol.* **44**, 400–409 (2016).

40. S. L. Prescott, C. Macaubas, B. J. Holt, T. B. Smallacombe, R. Loh, P. D. Sly, P. G. Holt, Transplacental priming of the human immune system to environmental allergens: Universal skewing of initial T cell responses toward the Th2 cytokine profile. *J. Immunol.* **160**, 4730–4737 (1998).

41. A. O'Neil, C. Itsiopoulos, H. Skouteris, R. S. Opie, S. McPhie, B. Hill, F. N. Jacka, Preventing mental health problems in offspring by targeting dietary intake of pregnant women. *BMC Med.* **12**, 208 (2014).

42. N. Güil-Oumrait, N. Stratakis, L. Maitre, A. Anguita-Ruiz, J. Urquiza, L. Fabbri, X. Basagaña, B. Heude, L. S. Haug, A. K. Sakhi, N. Iszatt, H. C. Keun, J. Wright, L. Chatzi, M. Vafeiadi, M. Bustamante, R. Grazuleviciene, S. Andrušaitytė, R. Slama, R. McEachan, M. Casas, M. Vrijheid, Prenatal exposure to chemical mixtures and metabolic syndrome risk in children. *JAMA Netw. Open* **7**, e2412040 (2024).

43. N. Stratakis, S. Rock, M. A. La Merrill, M. Saez, O. Robinson, D. Fecht, M. Vrijheid, D. Valvi, D. V. Conti, R. McConnell, V. L. Chatzi, Prenatal exposure to persistent organic pollutants and childhood obesity: A systematic review and meta-analysis of human studies. *Obes. Rev.* **23**, e13383 (2022).

44. M. Jerrett, R. McConnell, J. Wolch, R. Chang, C. Lam, G. Dunton, F. Gilliland, F. Lurmann, T. Islam, K. Berhane, Traffic-related air pollution and obesity formation in children: A longitudinal, multilevel analysis. *Environ. Health* **13**, 49 (2014).

45. R. McConnell, E. Shen, F. D. Gilliland, M. Jerrett, J. Wolch, C. C. Chang, F. Lurmann, K. Berhane, A longitudinal cohort study of body mass index and childhood exposure to secondhand tobacco smoke and air pollution: The Southern California Children's Health Study. *Environ. Health Perspect.* **123**, 360–366 (2015).

46. J. S. Kim, T. L. Alderete, Z. Chen, F. Lurmann, E. Rappaport, R. Habre, K. Berhane, F. D. Gilliland, Longitudinal associations of in utero and early life near-roadway air pollution with trajectories of childhood body mass index. *Environ. Health* **17**, 64 (2018).

47. J. L. Bolton, S. H. Smith, N. C. Huff, M. I. Gilmour, W. M. Foster, R. L. Auten, S. D. Bilbo, Prenatal air pollution exposure induces neuroinflammation and predisposes offspring to weight gain in adulthood in a sex-specific manner. *FASEB J.* **26**, 4743–4754 (2012).

48. X. Xu, Z. Yavar, M. Verdin, Z. Ying, G. Mihai, T. Kampfrath, A. Wang, M. Zhong, M. Lippmann, L. C. Chen, S. Rajagopalan, Q. Sun, Effect of early particulate air pollution exposure on obesity in mice: Role of p47phox. *Arterioscler. Thromb. Vasc. Biol.* **30**, 2518–2527 (2010).

49. Z. Xu, W. Wang, Q. Liu, Z. Li, L. Lei, L. Ren, F. Deng, X. Guo, S. Wu, Association between gaseous air pollutants and biomarkers of systemic inflammation: A systematic review and meta-analysis. *Environ. Pollut.* **292**, 118336 (2022).

50. C. R. Davis, J. M. Hodgson, R. Woodman, J. Bryan, C. Wilson, K. J. Murphy, A Mediterranean diet lowers blood pressure and improves endothelial function: Results from the MedLey randomized intervention trial. *Am. J. Clin. Nutr.* **105**, 1305–1313 (2017).

51. A. A. Bjerregaard, T. I. Halldorsson, I. Tetens, S. F. Olsen, Mother's dietary quality during pregnancy and offspring's dietary quality in adolescence: Follow-up from a national birth cohort study of 19,582 mother-offspring pairs. *PLOS Med.* **16**, e1002911 (2019).

52. M. La Merrill, C. Emond, M. J. Kim, J. P. Antignac, B. Le Bizec, K. Clément, L. S. Birnbaum, R. Barouki, Toxicological function of adipose tissue: Focus on persistent organic pollutants. *Environ. Health Perspect.* **121**, 162–169 (2013).

53. Z. Fewell, G. Davey Smith, J. A. C. Sterne, The impact of residual and unmeasured confounding in epidemiologic studies: A simulation study. *Am. J. Epidemiol.* **166**, 646–655 (2007).

54. C. J. Patel, J. P. A. Ioannidis, A. K. Manrai, An atlas of exposome–phenome associations in health and disease risk. *Nat. Med.* **32**, 1501–1510 (2026).

55. F. Perrier, L. Giorgis-Allemand, R. Slama, C. Philippat, Within-subject pooling of biological samples to reduce exposure misclassification in biomarker-based studies. *Epidemiology* **27**, 378–388 (2016).

56. M. Casas, X. Basagaña, A. K. Sakhi, L. S. Haug, C. Philippat, B. Granum, C. B. Manzano-Salgado, C. Brochot, F. Zeman, J. de Bont, S. Andrusaityte, L. Chatzi, D. Donaire-Gonzalez, L. Giorgis-Allemand, J. R. Gonzalez, E. Gracia-Lavedan, R. Grazuleviciene, M. Kampouri, S. Lyon-Caen, P. Pañella, I. Petraviciene, O. Robinson, J. Urquiza, M. Vafeiadi, C. Vernet, D. Waiblinger, J. Wright, C. Thomsen, R. Slama, M. Vrijheid, Variability of urinary concentrations of non-persistent chemicals in pregnant women and school-aged children. *Environ. Int.* **121**, 561–573 (2018).

57. L. S. Haug, A. K. Sakhi, E. Cequier, M. Casas, L. Maitre, X. Basagana, S. Andrusaityte, G. Chalkiadaki, L. Chatzi, M. Coen, J. de Bont, A. Dedele, J. Ferrand, R. Grazuleviciene, J. R. Gonzalez, K. B. Gutzkow, H. Keun, R. McEachan, H. M. Meltzer, I. Petraviciene, O. Robinson, P. J. Saulnier, R. Slama, J. Sunyer, J. Urquiza, M. Vafeiadi, J. Wright, M. Vrijheid, C. Thomsen, In-utero and childhood chemical exposome in six European mother-child cohorts. *Environ. Int.* **121**, 751–763 (2018).

58. W. Boyce, T. Torsheim, C. Currie, A. Zambon, The family affluence scale as a measure of national wealth: Validation of an adolescent self-report measure. *Soc. Indic. Res.* **78**, 473–487 (2006).

59. J. T. Flynn, D. C. Kaelber, C. M. Baker-Smith, D. Blowey, A. E. Carroll, S. R. Daniels, S. D. De Ferranti, J. M. Dionne, B. Falkner, S. K. Flinn, S. S. Gidding, C. Goodwin, M. G. Leu, M. E. Powers, C. Rea, J. Samuels, M. Simase, V. V. Thaker, E. M. Urbina, Subcommittee on Screening and Management of High Blood Pressure in Children, Clinical practice guideline for screening and management of high blood pressure in children and adolescents. *Pediatrics* **140**, e20171904 (2017).

60. I. R. White, P. Royston, A. M. Wood, Multiple imputation using chained equations: Issues and guidance for practice. *Stat. Med.* **30**, 377–399 (2011).

61. I. Tamayo-Uria, L. Maitre, C. Thomsen, M. J. Nieuwenhuijsen, L. Chatzi, V. Siroux, G. M. Aasvang, L. Agier, S. Andrusaityte, M. Casas, M. de Castro, A. Dedele, L. S. Haug, B. Heude, R. Grazuleviciene, K. B. Gutzkow, N. H. Krog, D. Mason, R. R. C. McEachan, H. M. Meltzer, I. Petraviciene, O. Robinson, T. Roumeliotaki, A. K. Sakhi, J. Urquiza, M. Vafeiadi, D. Waiblinger, C. Warembourg, J. Wright, R. Slama, M. Vrijheid, X. Basagaña, The early-life exposome: Description and patterns in six European countries. *Environ. Int.* **123**, 189–200 (2019).

62. J. Barrera-Gómez, L. Agier, L. Portengen, M. Chadeau-Hyam, L. Giorgis-Allemand, V. Siroux, O. Robinson, J. Vlaanderen, J. R. González, M. Nieuwenhuijsen, P. Vineis, M. Vrijheid, R. Vermeulen, R. Slama, X. Basagaña, A systematic comparison of statistical methods to detect interactions in exposome-health associations. *Environ. Health* **16**, 74 (2017).

63. L. Agier, L. Portengen, M. Chadeau-Hyam, X. Basagaña, L. Giorgis-Allemand, V. Siroux, O. Robinson, J. Vlaanderen, J. R. González, M. J. Nieuwenhuijsen, P. Vineis, M. Vrijheid, R. Slama, R. Vermeulen, A systematic comparison of linear regression–based statistical methods to assess exposome-health associations. *Environ. Health Perspect.* **124**, 1848–1856 (2016).

64. R. Beelen, G. Hoek, E. Pebesma, D. Vienneau, K. de Hoogh, D. J. Briggs, Mapping of background air pollution at a fine spatial scale across the European Union. *Sci. Total Environ.* **407**, 1852–1867 (2009).

65. J. Cyrys, M. Eeftens, J. Heinrich, C. Ampe, A. Armengaud, R. Beelen, T. Bellander, T. Beregszaszi, M. Birk, G. Cesaroni, M. Cirach, K. de Hoogh, A. De Nazelle, F. de Vocht, C. Declercq, A. Dedele, K. Dimakopoulou, K. Eriksen, C. Galassi, R. Graulevičiene, G. Grivas, O. Gruzieva, A. H. Gustafsson, B. Hoffmann, M. Iakovides, A. Ineichen, U. Krämer, T. Lanki, P. Lozano, C. Madsen, K. Meliefste, L. Modig, A. Mölter, G. Mosler, M. Nieuwenhuijsen, M. Nonnemacher, M. Oldenwening, A. Peters, S. Pontet, N. Probst-Hensch, U. Quass, O. Raaschou-Nielsen, A. Ranzi, D. Sugiri, E. G. Stephanou, P. Taimisto, M. Y. Tsai, É. Vaskövi, S. Villani, M. Wang, B. Brunekreef, G. Hoek, Variation of NO_2 and NO_x concentrations between and within 36 European study areas: Results from the ESCAPE study. *Atmos. Environ.* **62**, 374–390 (2012).

66. M. Eeftens, R. Beelen, K. De Hoogh, T. Bellander, G. Cesaroni, M. Cirach, C. Declercq, A. Dedele, E. Dons, A. De Nazelle, K. Dimakopoulou, K. Eriksen, G. Falq, P. Fischer, C. Galassi, R. Gražulevičiene, J. Heinrich, B. Hoffmann, M. Jerrett, D. Keidel, M. Korek, T. Lanki, S. Lindley, C. Madsen, A. Mölter, G. Nádor, M. Nieuwenhuijsen, M. Nonnemacher, X. Pedeli, O. Raaschou-Nielsen, E. Patelarou, U. Quass, A. Ranzi, C. Schindler, M. Stempfelet, E. Stephanou, D. Sugiri, M. Y. Tsai, T. Yli-Tuomi, M. J. Varró, D. Vienneau, S. Von Klot, K. Wolf, B. Brunekreef, G. Hoek, Development of land use regression models for $PM_{2.5}$, $PM_{2.5}$ absorbance, PM_{10} and PM_{coarse} in 20 European study areas; Results of the ESCAPE Project. *Environ. Sci. Technol.* **46**, 11195–11205 (2012).

67. M. Eeftens, M. Y. Tsai, C. Ampe, B. Anwander, R. Beelen, T. Bellander, G. Cesaroni, M. Cirach, J. Cyrys, K. de Hoogh, A. De Nazelle, F. de Vocht, C. Declercq, A. Dedele, K. Eriksen, C. Galassi, R. Gražulevičiene, G. Grivas, J. Heinrich, B. Hoffmann, M. Iakovides, A. Ineichen, K. Katsouyanni, M. Korek, U. Krämer, T. Kuhlbusch, T. Lanki, C. Madsen, K. Meliefste, A. Mölter, G. Mosler, M. Nieuwenhuijsen, M. Oldenwening, A. Pennanen, N. Probst-Hensch, U. Quass, O. Raaschou-Nielsen, A. Ranzi, E. Stephanou, D. Sugiri, O. Udvardy, É. Vaskövi, G. Weinmayr, B. Brunekreef, G. Hoek, Spatial variation of $PM_{2.5}$, PM_{10}, $PM_{2.5}$ absorbance and PM_{coarse} concentrations between and within 20 European study areas and the relationship with NO_2—Results of the ESCAPE project. *Atmos. Environ.* **62**, 303–317 (2012).

68. R. Beelen, G. Hoek, D. Vienneau, M. Eeftens, K. Dimakopoulou, X. Pedeli, M. Y. Tsai, N. Künzli, T. Schikowski, A. Marcon, K. T. Eriksen, O. Raaschou-Nielsen, E. Stephanou, E. Patelarou, T. Lanki, T. Yli-Tuomi, C. Declercq, G. Falq, M. Stempfelet, M. Birk, J. Cyrys, S. von Klot, G. Nádor, M. J. Varró, A. Dedele, R. Gražulevičiene, A. Mölter, S. Lindley, C. Madsen, G. Cesaroni, A. Ranzi, C. Badaloni, B. Hoffmann, M. Nonnemacher, U. Krämer, T. Kuhlbusch, M. Cirach, A. de Nazelle, M. Nieuwenhuijsen, T. Bellander, M. Korek, D. Olsson, M. Strömgren, E. Dons, M. Jerrett, P. Fischer, M. Wang, B. Brunekreef, K. de Hoogh, Development of NO_2 and NO_x land use regression models for estimating air pollution exposure in 36 study areas in Europe—The ESCAPE project. *Atmos. Environ.* **72**, 10–23 (2013).

69. A. Schembari, K. De Hoogh, M. Pedersen, P. Dadvand, D. Martinez, G. Hoek, E. S. Petherick, J. Wright, M. J. Nieuwenhuijsen, Ambient air pollution and newborn size and adiposity at birth: Differences by maternal ethnicity (the Born in Bradford study cohort). *Environ. Health Perspect.* **123**, 1208–1215 (2015).

70. M. Wang, R. Beelen, T. Bellander, M. Birk, G. Cesaroni, M. Cirach, J. Cyrys, K. de Hoogh, C. Declercq, K. Dimakopoulou, M. Eeftens, K. T. Eriksen, F. Forastiere, C. Galassi, G. Grivas, J. Heinrich, B. Hoffmann, A. Ineichen, M. Korek, T. Lanki, S. Lindley, L. Modig, A. Mölter, P. Nafstad, M. J. Nieuwenhuijsen, W. Nystad, D. Olsson, O. Raaschou-Nielsen, M. Ragettli, A. Ranzi, M. Stempfelet, D. Sugiri, M. Y. Tsai, O. Udvardy, M. J. Varró, D. Vienneau, G. Weinmayr, K. Wolf, T. Yli-Tuomi, G. Hoek, B. Brunekreef, Performance of multi-city land use regression models for nitrogen dioxide and fine particles. *Environ. Health Perspect.* **122**, 843–849 (2014).

71. A. Rahmalia, L. Giorgis-Allemand, J. Lepeule, C. Philippat, J. Galineau, A. Hulin, M. A. Charles, R. Slama, EDEN Mother-Child Cohort Study group, Pregnancy exposure to atmospheric pollutants and placental weight: An approach relying on a dispersion model. *Environ. Int.* **48**, 47–55 (2012).

72. M. J. Nieuwenhuijsen, H. Kruize, C. Gidlow, S. Andrusaityte, J. M. Antó, X. Basagaña, M. Cirach, P. Dadvand, A. Danileviciute, D. Donaire-Gonzalez, J. Garcia, M. Jerrett, M. Jones, J. Julvez, E. Van Kempen, I. Van Kamp, J. Maas, E. Seto, G. Smith, M. Triguero, W. Wendel-

Vos, J. Wright, J. Zufferey, P. J. Van Den Hazel, R. Lawrence, R. Grazuleviciene, Positive health effects of the natural outdoor environment in typical populations in different regions in Europe (PHENOTYPE): A study programme protocol. *BMJ Open* **4**, e004951 (2014).

73. J. Herring, Measuring Vegetation (NDVI & EVI): Feature Articles (NASA Earth Observatory, 2000).

74. A. Smargiassi, M. S. Goldberg, C. Plante, M. Fournier, Y. Baudouin, T. Kosatsky, Variation of daily warm season mortality as a function of micro-urban heat islands. *J. Epidemiol. Community Health* **63**, 659–664 (2009).

75. D. T. Duncan, J. Aldstadt, J. Whalen, S. J. Melly, S. L. Gortmaker, Validation of walk score for estimating neighborhood walkability: An analysis of four US metropolitan areas. *Int. J. Environ. Res. Public Health* **8**, 4160–4179 (2011).

76. L. D. Frank, J. F. Sallis, T. L. Conway, J. E. Chapman, B. E. Saelens, W. Bachman, Many pathways from land use to health: Associations between neighborhood walkability and active transportation, body mass index, and air quality. *J. Am. Plann. Assoc.* **72**, 75–87 (2006).

77. E. Van Nunen, R. Vermeulen, M. Y. Tsai, N. Probst-Hensch, A. Ineichen, M. Davey, M. Imboden, R. Ducret-Stich, A. Naccarati, D. Raffaele, A. Ranzi, C. Ivaldi, C. Galassi, M. Nieuwenhuijsen, A. Curto, D. Donaire-Gonzalez, M. Cirach, L. Chatzi, M. Kampouri, J. Vlaanderen, K. Meliefste, D. Buijtenhuijs, B. Brunekreef, D. Morley, P. Vineis, J. Gulliver, G. Hoek, Land use regression models for ultrafine particles in six European areas. *Environ. Sci. Technol.* **51**, 3336–3345 (2017).

78. C. H. Jeong, E. D. Wagner, V. R. Siebert, S. Anduri, S. D. Richardson, E. J. Daiber, A. B. McKague, M. Kogevinas, C. M. Villanueva, E. H. Goslan, W. Luo, L. M. Isabelle, J. F. Pankow, R. Grazuleviciene, S. Cordier, S. C. Edwards, E. Righi, M. J. Nieuwenhuijsen, M. J. Plewa, Occurrence and toxicity of disinfection byproducts in European drinking waters in relation with the HIWATE epidemiology study. *Environ. Sci. Technol.* **46**, 12120–12128 (2012).

79. R. B. Smith, S. C. Edwards, N. Best, J. Wright, M. J. Nieuwenhuijsen, M. B. Toledano, Birth weight, ethnicity, and exposure to trihalomethanes and haloacetic acids in drinking water during pregnancy in the Born in Bradford cohort. *Environ. Health Perspect.* **124**, 681–689 (2016).

80. C. M. Villanueva, E. Gracia-Lavedán, J. Ibarluzea, L. S. Marina, F. Ballester, S. Llop, A. Tardón, M. F. Fernández, C. Freire, F. Goñi, X. Basagaña, M. Kogevinas, J. O. Grimalt, J. Sunyer, INMA (Infancia y Medio Ambiente) Project, Exposure to trihalomethanes through different water uses and birth weight, small for gestational age, and preterm delivery in Spain. *Environ. Health Perspect.* **119**, 1824–1830 (2011).

81. A. Danileviciute, R. Grazuleviciene, J. Vencloviene, A. Paulauskas, M. J. Nieuwenhuijsen, Exposure to drinking water trihalomethanes and their association with low birth weight and small for gestational age in genetically susceptible women. *Int. J. Environ. Res. Public Health* **9**, 4470–4485 (2012).

82. I. H. Caspersen, H. E. Kvalem, M. Haugen, A. L. Brantsæter, H. M. Meltzer, J. Alexander, C. Thomsen, M. Frøshaug, N. M. B. Bremnes, S. L. Broadwell, B. Granum, M. Kogevinas, H. K. Knutsen, Determinants of plasma PCB, brominated flame retardants, and organochlorine pesticides in pregnant women and 3 year old children in The Norwegian Mother and Child Cohort Study. *Environ. Res.* **146**, 136–144 (2016).

83. F. Goñi, R. López, A. Etxeandia, E. Millán, P. Amiano, High throughput method for the determination of organochlorine pesticides and polychlorinated biphenyls in human serum. *J. Chromatogr. B Anal. Technol. Biomed. Life Sci.* **852**, 15–21 (2007).

84. C. B. Manzano-Salgado, M. Casas, M. J. Lopez-Espinosa, F. Ballester, M. Basterrechea, J. O. Grimalt, A. M. Jiménez, T. Kraus, T. Schettgen, J. Sunyer, M. Vrijheid, Transfer of perfluoroalkyl substances from mother to fetus in a Spanish birth cohort. *Environ. Res.* **142**, 471–478 (2015).

85. S. Poothong, C. Thomsen, J. A. Padilla-Sanchez, E. Papadopoulou, L. S. Haug, Distribution of novel and well-known poly- and perfluoroalkyl substances (PFASs) in human serum, plasma, and whole blood. *Environ. Sci. Technol.* **51**, 13388–13396 (2017).

86. I. Rodushkin, M. D. Axelsson, Application of double focusing sector field ICP-MS for multielemental characterization of human hair and nails. Part I. Analytical methodology. *Sci. Total Environ.* **250**, 83–100 (2000).

87. R. Ramon, M. Murcia, X. Aguinagalde, A. Amurrio, S. Llop, J. Ibarluzea, A. Lertxundi, M. Alvarez-Pedrerol, M. Casas, J. Vioque, J. Sunyer, A. Tardon, B. Martinez-Arguelles, F. Ballester, Prenatal mercury exposure in a multicenter cohort study in Spain. *Environ. Int.* **37**, 597–604 (2011).

88. A. H. Stern, A. E. Smith, An assessment of the cord blood:maternal blood methylmercury ratio: Implications for risk assessment. *Environ. Health Perspect.* **111**, 1465–1470 (2003).

89. M. A. Padilla, M. Elobeid, D. M. Ruden, D. B. Allison, An examination of the association of selected toxic metals with total and central obesity indices: NHANES 99-02. *Int. J. Environ. Res. Public Health* **7**, 3332–3347 (2010).

90. A. Sabaredzovic, A. K. Sakhi, A. L. Brantsæter, C. Thomsen, Determination of 12 urinary phthalate metabolites in Norwegian pregnant women by core–shell high performance liquid chromatography with on-line solid-phase extraction, column switching and tandem mass spectrometry. *J. Chromatogr. B* **1002**, 343–352 (2015).

91. D. Valvi, N. Monfort, R. Ventura, M. Casas, L. Casas, J. Sunyer, M. Vrijheid, Variability and predictors of urinary phthalate metabolites in Spanish pregnant women. *Int. J. Hyg. Environ. Health* **218**, 220–231 (2015).

92. C. Philippat, M. Mortamais, C. Chevrier, C. Petit, A. M. Calafat, X. Ye, M. J. Silva, C. Brambilla, I. Pin, M. A. Charles, S. Cordier, R. Slama, Exposure to phthalates and phenols during pregnancy and offspring size at birth. *Environ. Health Perspect.* **120**, 464–470 (2012).

93. E. Cequier, A. K. Sakhi, L. S. Haug, C. Thomsen, Development of an ion-pair liquid chromatography–high resolution mass spectrometry method for determination of organophosphate pesticide metabolites in large-scale biomonitoring studies. *J. Chromatogr. A* **1454**, 32–41 (2016).

94. J. J. Aurrekoetxea, M. Murcia, M. Rebagliato, M. J. López, A. M. Castilla, L. Santa-Marina, M. Guxens, A. Fernández-Somoano, M. Espada, A. Lertxundi, A. Tardón, F. Ballester, Determinants of self-reported smoking and misclassification during pregnancy, and analysis of optimal cut-off points for urinary cotinine: A cross-sectional study. *BMJ Open* **3**, e002034 (2013).

95. J. Sunyer, R. Garcia-Esteban, A. M. Castilla, J. J. Aurrekoetxea, C. Iñiguez, A. Tardón, M. Espada, A. Lertxundi, L. Chatzi, M. Rebagliato, M. Kogevinas, INMA project, Exposure to second-hand smoke and reproductive outcomes depending on maternal asthma. *Eur. Respir. J.* **40**, 371–376 (2012).

96. A. Covaci, S. Voorspoels, C. Thomsen, B. van Bavel, H. Neels, Evaluation of total lipids using enzymatic methods for the normalization of persistent organic pollutant levels in serum. *Sci. Total Environ.* **366**, 361–366 (2006).

97. E. Grimvall, L. Rylander, P. Nilsson-Ehle, U. Nilsson, U. Strömberg, L. Hagmar, C. Östman, Monitoring of polychlorinated biphenyls in human blood plasma: Methodological developments and influence of age, lactation, and fish consumption. *Arch. Environ. Contam. Toxicol.* **32**, 329–336 (1997).

98. Sedentary Behaviour Research Network, Letter to the editor: Standardized use of the terms "sedentary" and "sedentary behaviours". *Appl. Physiol. Nutr. Metab.* **37**, 540–542 (2012).

99. Y. Liu, M. Wang, J. Villberg, T. Torsheim, J. Tynjälä, Y. Lv, L. Kannas, Reliability and validity of Family Affluence Scale (FAS II) among adolescents in Beijing, China. *Child Indic. Res.* **5**, 235–251 (2011).

100. Y. Jin, M. J. Hein, J. A. Deddens, C. J. Hines, Analysis of lognormally distributed exposure data with repeated measures and values below the limit of detection using SAS. *Ann. Occup. Hyg.* **55**, 97–112 (2011).

101. E. A. Stuart, M. Azur, C. Frangakis, P. Leaf, Multiple imputation with large data sets: A case study of the Children's Mental Health Initiative. *Am. J. Epidemiol.* **169**, 1133–1139 (2009).

102. S. van Buuren, K. Groothuis-Oudshoorn, mice: Multivariate Imputation by Chained Equations in R. *J. Stat. Softw.* **45**, 1–67 (2011).

103. L. Chen, J. Z. Huang, Sparse reduced-rank regression for simultaneous dimension reduction and variable selection. *J. Am. Stat. Assoc.* **107**, 1533–1545 (2012).

104. B. Bodinier, S. Filippi, T. H. Nøst, J. Chiquet, M. Chadeau-Hyam, Automated calibration for stability selection in penalised regression and graphical models. *J. R. Stat. Soc. Ser. C Appl. Stat.* **72**, 1375–1393 (2023).

105. B. Bodinier, S. Rodrigues, M. Karimi, S. Filippi, J. Chiquet, M. Chadeau-Hyam, Stability selection and consensus clustering in R: The R package sharp. *J. Stat. Softw.* **112**, 1–27 (2025).

106. J. Koponen, P. Rantakokko, R. Airaksinen, H. Kiviranta, Determination of selected perfluorinated alkyl acids and persistent organic pollutants from a small volume human serum sample relevant for epidemiological studies. *J. Chromatogr. A* **1309**, 48–55 (2013).

107. L. S. Haug, C. Thomsen, G. Becher, Time trends and the influence of age and gender on serum concentrations of perfluorinated compounds in archived human samples. *Environ. Sci. Technol.* **43**, 2131–2136 (2009).

108. I. Garali, I. Adanyeguh, F. Ichou, V. Perlbarg, A. Seyer, B. Colsch, I. Moszer, V. Guillemot, A. Durr, F. Mochel, A. Tenenhaus, A strategy for multimodal data integration: Application to biomarkers identification in spinocerebellar ataxia. *Brief. Bioinform.* **19**, 1356–1369 (2018).
